# Supplementary material for: Transient caspase-mediated activation of caspase-activated DNase causes DNA damage required for phagocytic macrophage differentiation
Source: Cell Rep. Author manuscript; Available in PMC 2025 Jan 9. (PMC7617294; doi:10.1016/j.celrep.2024.114251)
Supplement: Supplementary Information [file EMS202103-supplement-Supplementary_Information.zip › 1-s2.0-S2211124724005795-mmc1.pdf]

Cell Reports, Volume *43*

Supplemental information

Transient caspase-mediated activation of caspase-activated DNase causes DNA damage required for phagocytic macrophage differentiation

Deepak Maurya, Gayatri Rai, Debleena Mandal, and Bama Charan Mondal

Figure S1

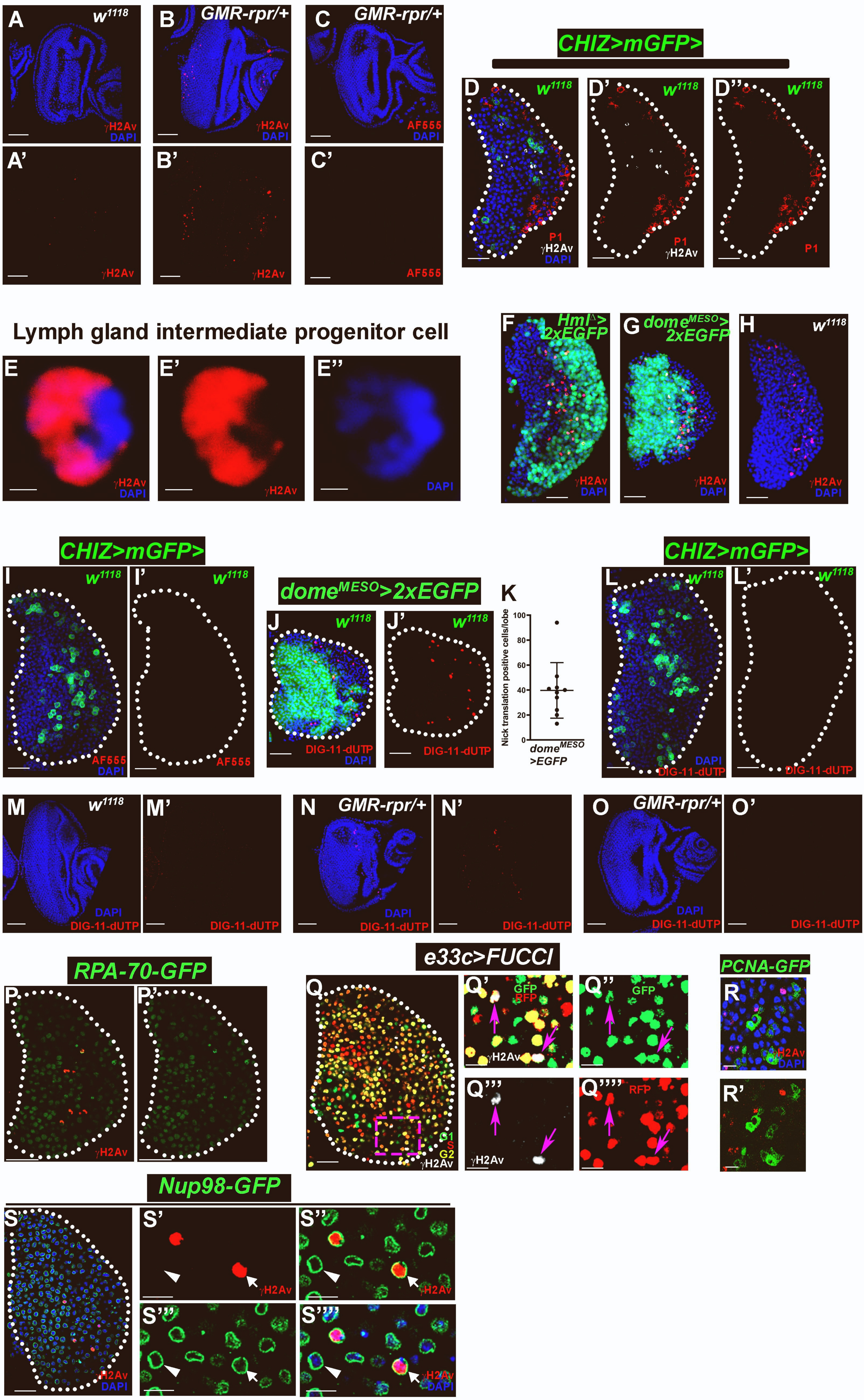

A w1118
B GMR-rpr/+
C GMR-rpr/+
γH2Av DAPI
AF555 DAPI
A'
B'
C'
γH2Av
AF555
CHIZ>mGFP>
D w1118
D' w1118
D'' w1118
P1 γH2Av DAPI
P1 γH2Av
P1
Lymph gland intermediate progenitor cell
E
E'
E''
γH2Av DAPI
γH2Av
DAPI
F HmlΔ> 2xEGFP
G domeMESO> 2xEGFP
H w1118
CHIZ>mGFP>
I w1118
I' w1118
AF555 DAPI
AF555
domeMESO>2xEGFP
J w1118
J' w1118
DIG-11-dUTP DAPI
DIG-11-dUTP
K
Nick translation positive cells/lobe
100
80
60
40
20
0
domeMESO >EGFP
CHIZ>mGFP>
L w1118
L' w1118
DAPI DIG-11-dUTP
M w1118
M'
N GMR-rpr/+
N'
O GMR-rpr/+
O'
RPA-70-GFP
P
P'
e33c>FUCCI
Q
Q' GFP RFP
Q'' GFP
Q'''
Q'''' RFP
G1 S G2 γH2Av
PCNA-GFP
R
R'
H2Av DAPI
Nup98-GFP
S
S'
S''
S'''
S''''

Figure S1. DNA damage occurs during the differentiation of lymph gland

(A-C') The γH2Av staining (red) increased in the eye disc expressing rpr (*GMR-rpr/+*) (B-B') in comparison to the control eye disc (w^{1118}) (A-A'). Also, negative control (GMR-rpr/+), which is not incubated with primary antibody (mouse-anti-γH2Av, Cat# UNC93-5.2.1) and only incubated with secondary antibody (AF555, Cat# A31570), shows no staining (C-C').

(D-D'') The co-immunostaining of γH2Av (grey) and mature macrophage marker P1 (red) show no colocalization.

(E-E'') The γH2Av staining (red) covers the entire nuclear region, except the DAPI (blue)-bright heterochromatin region in the lymph gland intermediate progenitor.

(F-H) *Hml^{Δ}-Gal4, UAS-2xEGFP/+* (green) (F), *$dome^{MESO}$-Gal4, UAS-2xEGFP/+* (green) (G) and w^{1118} (H) genetic backgrounds show a similar number of γH2Av positive cells (red) in the differentiating zone.

(I-I') Negative control immunostaining (red) for lymph glands that are incubated without primary antibody (mouse-anti-γH2Av) shows no staining.

(J-J') Nick translation (red) shows the incorporation of DIG-11dUTP in the cells of the differentiating zone (low GFP) of the control lymph gland, indicating DNA strand breaks *$dome^{MESO}$-Gal4, UAS-2xEGFP/+* (green) (n=10).

(K) Quantification of nick translation positive cell number represented in (J-J').

(L-L') The negative control for nick translation (red) for the lymph gland not incubated with the enzyme (DNA polymerase I) shows no incorporation of DIG-11dUTP in the cells.

(M-O') The nick translation (red) increased in the eye disc expressing *rpr* (*GMR-rpr/+*) (N-N') in comparison to the control eye disc (w^{1118}) (M-M'). Also, the negative control (GMR-rpr/+), which is incubated without enzyme (DNA polymerase I), shows no incorporation of DIG-11dUTP in the cells (O-O').

(P-P') γH2Av staining (red) colocalized with *RPA70-GFP* (green) in the lymph gland.

(Q-Q'''') Expression of *e33c-Gal4, UAS-FUCCI* shows the cell cycle status of lymph gland G2 (yellow), G1 (green), and S (red) phases (Q) and the colocalization of G2 cells with γH2Av (grey). The insets show the colocalization of γH2Av-positive cells with GFP and RFP, indicated by the arrows in the G2 phase (Q'-Q'''').

(R-R') γH2Av staining (red) in *PCNA-GFP* (green) cells as S phase markers do not colocalize in the lymph gland, nuclei stained with DAPI (blue).

(S-S'''') γH2Av staining (red) in *Nup98-GFP* (green) with DAPI (blue) (S) and high magnification image of γH2Av in *Nup-98-GFP* background only γH2Av (S'), γH2Av with *Nup98-GFP* shows intact nuclear membrane (S''), only *Nup98-GFP* (S''') and γH2Av, *Nup98-GFP* with DAPI (S''''). Arrow marks the γH2Av positive nuclei and intact nuclear pore complexes compared with γH2Av negative (marked arrowhead) nuclei in the lymph gland.

All images are from the wandering third instar lymph gland except images A-C' and M-O' from the wandering third instar eye disc. Scale bars: 25µm in all images except 50 µm in (A-C' and M-O'), 10µm in (Q'-R' and S'-S''''), 1µm in (E-E''). All images are single optical sections except images (F-J' and L-L'), which are the maximum intensity projections of the middle third optical sections. Nuclei stained with DAPI (blue). The lymph glands boundary is demarcated by a white dotted line for clarity. Error bars, mean ± SD. All images are representative of 3 or more independent biological experiments, and 'n' represents a number of lymph gland lobes.

Figure S2

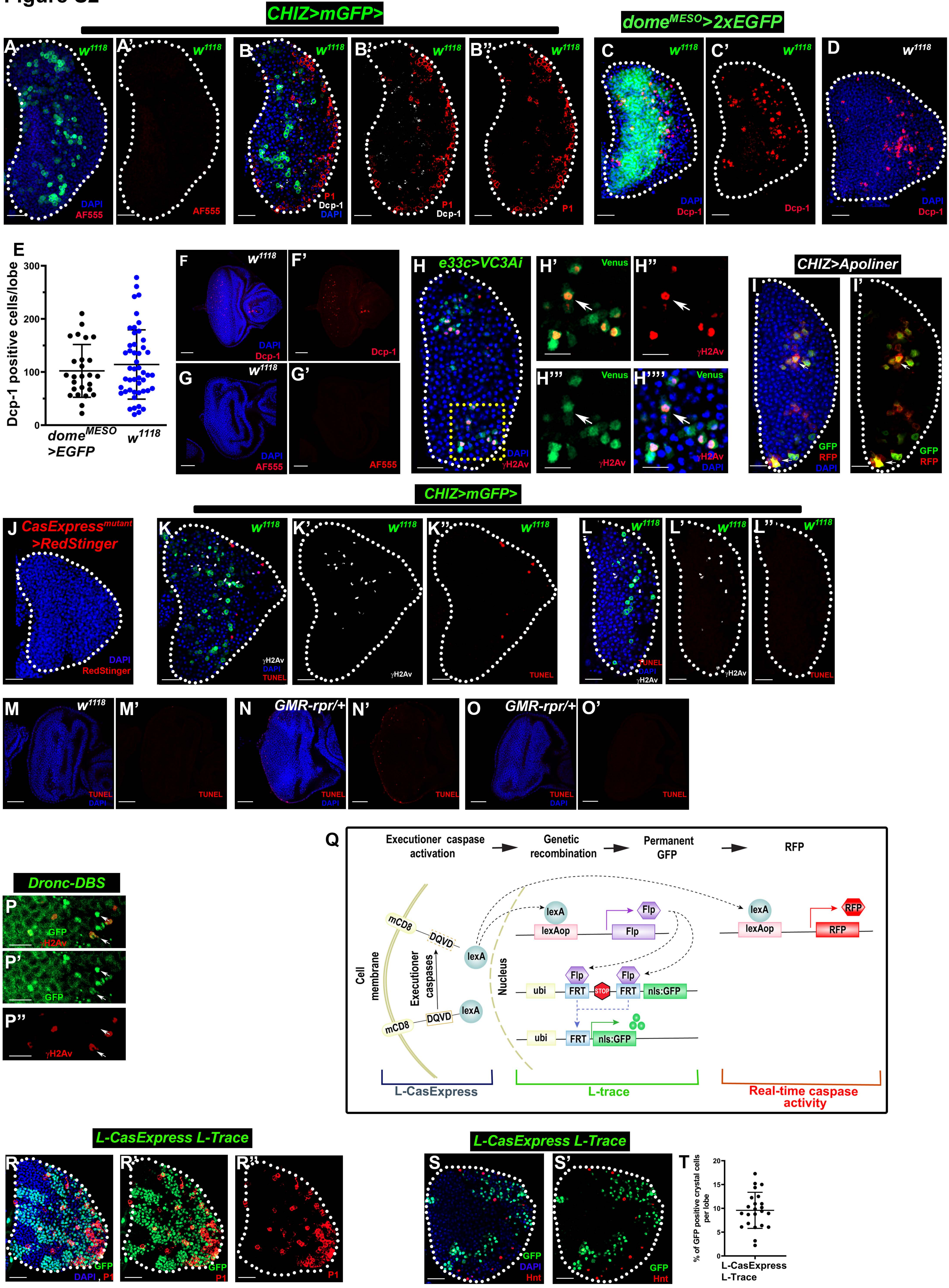

CHIZ>mGFP>
dome^MESO>2xEGFP
Dcp-1 positive cells/lobe
dome^MESO >EGFP
w1118
e33c>VC3Ai
CHIZ>Apoliner
CasExpress^mutant >RedStinger
GMR-rpr/+
Dronc-DBS
Executioner caspase activation
Genetic recombination
Permanent GFP
RFP
Cell membrane
Executioner caspases
Nucleus
L-CasExpress
L-trace
Real-time caspase activity
L-CasExpress L-Trace
% of GFP positive crystal cells per lobe

Figure S2. DNA breaks and active caspase in differentiating progenitor blood cells

(A-A') Negative-control immunostaining (red) shows no staining for lymph glands incubated without primary antibody (anti-Dcp1, CST Cat# 9578S) and only with secondary antibody (AF555, Cat# A31572).

(B-B'') The co-immunostaining of Dcp-1 (grey) and mature macrophage marker P1 (red) show no colocalization.

(C-D) Dcp-1 staining (red) is shown in *dome*^{MESO}*-Gal4, UAS-2xEGFP/*+ progenitors mark green (C-C') (n=26) and *w*^{1118} (D) (n=48) genetic background of the lymph gland in the intermediate zone.

(E) Quantification of Dcp-1 positive cell number in (C-D).

(F-G') The Dcp-1 staining (red) found in wild-type control eye disc (*w*^{1118}) (F-F'); however, negative control, which is incubated without primary antibody (cleaved-Dcp-1) and only incubated with secondary antibody (AF555), shows no staining (G-G').

(H-H'''') Fluorescent reporter (green) of executioner caspase (*e33c-Gal4, UAS-VC3Ai*) are shown with γH2Av staining (red) (H). Insets show venus (green) colocalization with γH2Av (arrow).

(I-I') Expression of *CHIZ-Gal4, UAS-Apoliner* show membrane RFP, membrane GFP, and nuclear GFP (marks caspase-active cell); arrow indicates caspase-active cell.

(J) Expression of *CasExpress*^{Mutant}*-Gal4, UAS- RedStinger* does not show executioner activity in the lymph gland, which is used as a control for *CasExpress-Gal4* (Figure 2J).

(K-K'') The lymph glands γH2Av-positive cells (grey) are not high-intensity TUNEL-positive cells (red).

(L-L'') Negative control for TUNEL staining (red) for lymph gland which is not incubated with enzyme (terminal deoxynucleotidyl transferase) shows no TUNEL positive cells but γH2Av-positive cells present (grey).

(M-O') The TUNEL staining (red) increased in the eye disc expressing rpr (*GMR-rpr/*+) (N-N') in comparison to the control eye disc (*w*^{1118}) (M-M'). Also, negative control (GMR-rpr/+), which is not incubated with enzyme (terminal deoxynucleotidyl transferase), shows no TUNEL-positive cells (O-O').

(P-P'') High-magnification image of γH2Av (red) staining in *Drice-Based-Sensor-GFP* (*DBS-GFP*; green) (P), only γH2Av (P'') and only GFP (P') shows γH2Av colocalization with low DBS-GFP cells.

(Q) A model showing the mechanistic principle underlying how the *L-CasExpress L-Trace* works (adapted from Sun G *et al.* 2021).

(R-R'') Mature macrophage marker P1 staining (red) in the *L-CasExpress L-Trace* (*lex-Aop-Flp::Ubi-FRT-STOP-FRT-GFP/+; L-caspase/*+) show colocalization with lineage trace GFP suggesting cascade active cells survive and become mature macrophage and only P1 (R'').

(S-S') Crystal cell marker Hnt staining (red) in the *L-CasExpress L-Trace* (*lex-Aop-Flp::Ubi-FRT-STOP-FRT-GFP/+; L-caspase/*+) show significantly less colocalization with lineage trace GFP (S) and only Hnt (S').

(T) Quantification of the percentage of crystal cells in *L-CasExpress L-Trace* (*lex-Aop-Flp::Ubi-FRT-STOP-FRT-GFP/+; L-caspase/*+) colocalized with lineage trace GFP(green) and DAPI (blue) (n=23).

All images are from the wandering third instar lymph gland except images F-G' and M-O' from the wandering third instar eye disc. Scale bars: 25µm in all images except 50 µm in (F-

G’ and M-O’) and 10µm in (H’-H’’’’ and P-P’’). All images are maximum intensity projections of the middle third optical sections except images (B-B’’, F-I’, K-P’’ and S-S’), which are single optical sections. Nuclei stained with DAPI (blue). The lymph glands boundary is demarcated by a white dotted line for clarity. Error bars, mean ± SD. All images are representative of 3 or more independent biological experiments, and ‘n’ represents the - number of lymph gland lobes.

Figure S3

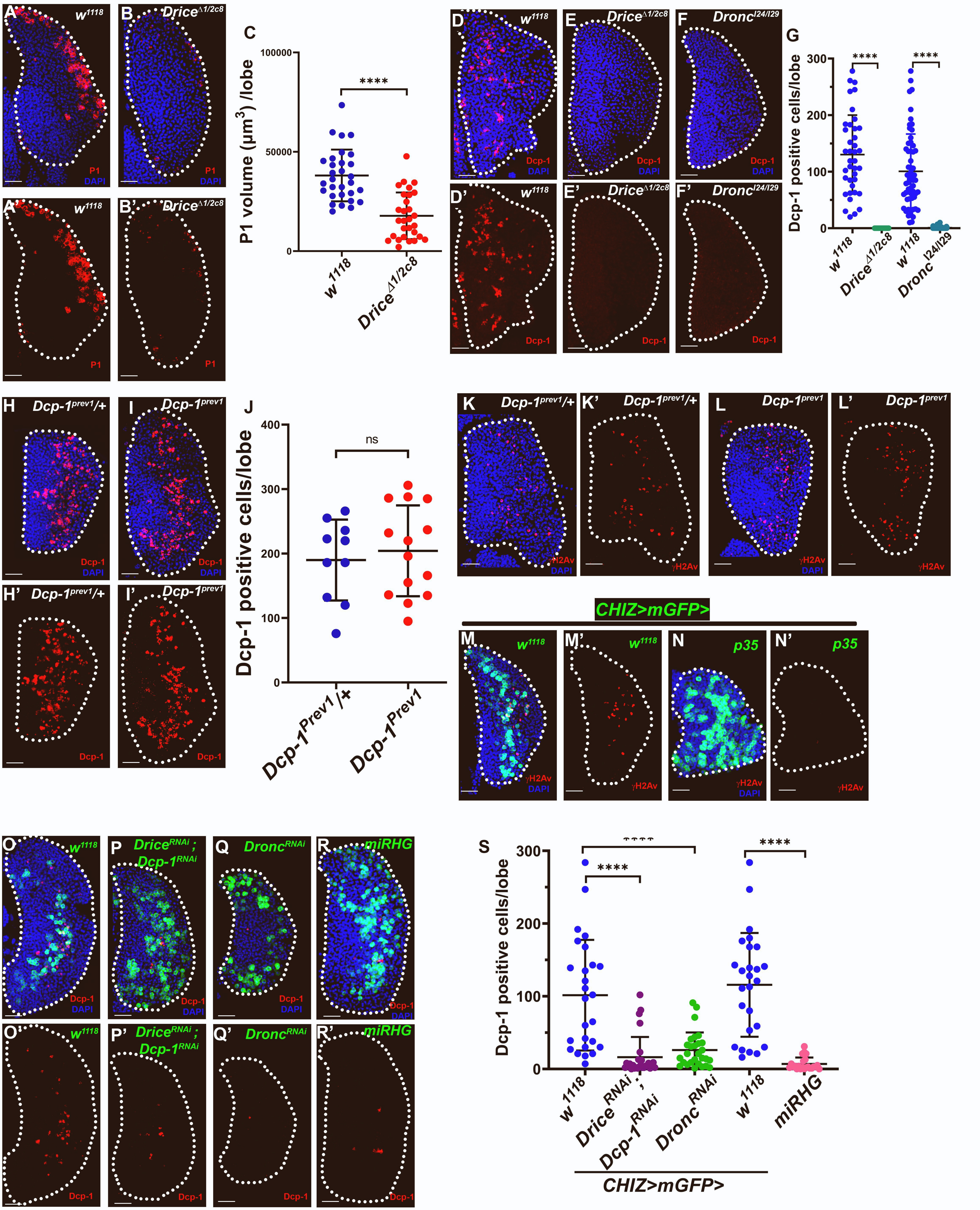

A w1118
B Drice∆1/2c8
A' w1118
B' Drice∆1/2c8
P1
DAPI
C
P1 volume (µm3) /lobe
100000
50000
0
****
w1118
Drice∆1/2c8
D w1118
E Drice∆1/2c8
F DroncI24/I29
D' w1118
E' Drice∆1/2c8
F' DroncI24/I29
Dcp-1
DAPI
G
Dcp-1 positive cells/lobe
300
200
100
0
****
****
w1118
Drice∆1/2c8
w1118
DroncI24/I29
H Dcp-1prev1/+
I Dcp-1prev1
H' Dcp-1prev1/+
I' Dcp-1prev1
J
Dcp-1 positive cells/lobe
400
300
200
100
0
ns
Dcp-1Prev1/+
Dcp-1Prev1
K Dcp-1prev1/+
K' Dcp-1prev1/+
L Dcp-1prev1
L' Dcp-1prev1
γH2Av
CHIZ>mGFP>
M w1118
M' w1118
N p35
N' p35
O w1118
P DriceRNAi ; Dcp-1RNAi
Q DroncRNAi
R miRHG
O' w1118
P' DriceRNAi ; Dcp-1RNAi
Q' DroncRNAi
R' miRHG
S
Dcp-1 positive cells/lobe
300
200
100
0
****
****
****
w1118
DriceRNAi ; Dcp-1RNAi
DroncRNAi
w1118
miRHG
CHIZ>mGFP>

Figure S3. Caspase-mediated DNA breaks needs for macrophage differentiation

(A-B') Mature macrophage marker P1 staining (red) in control *w*^{1118} (A-A') (n=30), which is significantly reduced in *Drice*$^{\Delta 1}$*/Drice*^{2c8} (B-B') (n=29).

(C) Quantification of P1 volume in (A-B').

(D-F') Dcp-1-positive cells (red) in comparison to control (*w*^{1118}) sets (D-D') (n=39 and 59), absent in *Drice*$^{\Delta 1}$*/Drice*^{2c8} (E-E') (n=41) and *Dronc*^{l29}*/Dronc*^{l24} (F-F') (n=58) mutants.

(G) Quantification of Dcp-1-positive cell number in (D-F').

(H-I') Dcp-1-positive cells (red) in Dcp-1 heterozygous mutant (*Dcp-1*^{Prev1}/+) (H-H') (n=10) and homozygous mutant (*Dcp-1*^{Prev1}) (I-I') (n=14) remain unchanged.

(J) Quantification of Dcp-1-positive cell number in (H-I')

(K-L') γH2Av -positive cells (red) in Dcp-1 heterozygous mutant (*Dcp-1*^{Prev1}/+) (K-K') and homozygous mutant (*Dcp-1*^{Prev1}) (L-L') remain unchanged.

(M-N') The *CHIZ> mGFP*-driven *UAS-p35* background (N-N') shows very few γH2Av-positive cells (red) compared to the control (M-M').

(O-R') γH2Av-positive cells(red) in comparison to control sets [*CHIZ>mGFP/+* (O-O') (n=26 and n=26), *CHIZ>mGFP* driven *RNAi, UAS-Drice*^{RNAi}*; UAS-Dcp-1*^{RNAi} (P-P') (n=28), *UAS-Dronc*^{RNAi} (Q-Q') (n=28), *UAS-miRHG* (R-R') (n=23) show significantly less γH2Av-positive cells.

(S) Quantification of γH2Av-positive cell number in (O-R').

All images show a 25µm scale bar, with maximum intensity projections of the middle third optical section of the wandering third instar larval lymph gland lobe. Nuclei stained with DAPI (blue). The lymph glands boundary is demarcated by a white dotted line for clarity. ****P < 0.0001 ns- not significant. Error bars, mean ± SD. Control groups are different for their respective experimental sets because experiments are performed on different days. All images are representative of 3 or more independent biological experiments, and 'n' represents a number of lymph gland lobes.

Figure S4

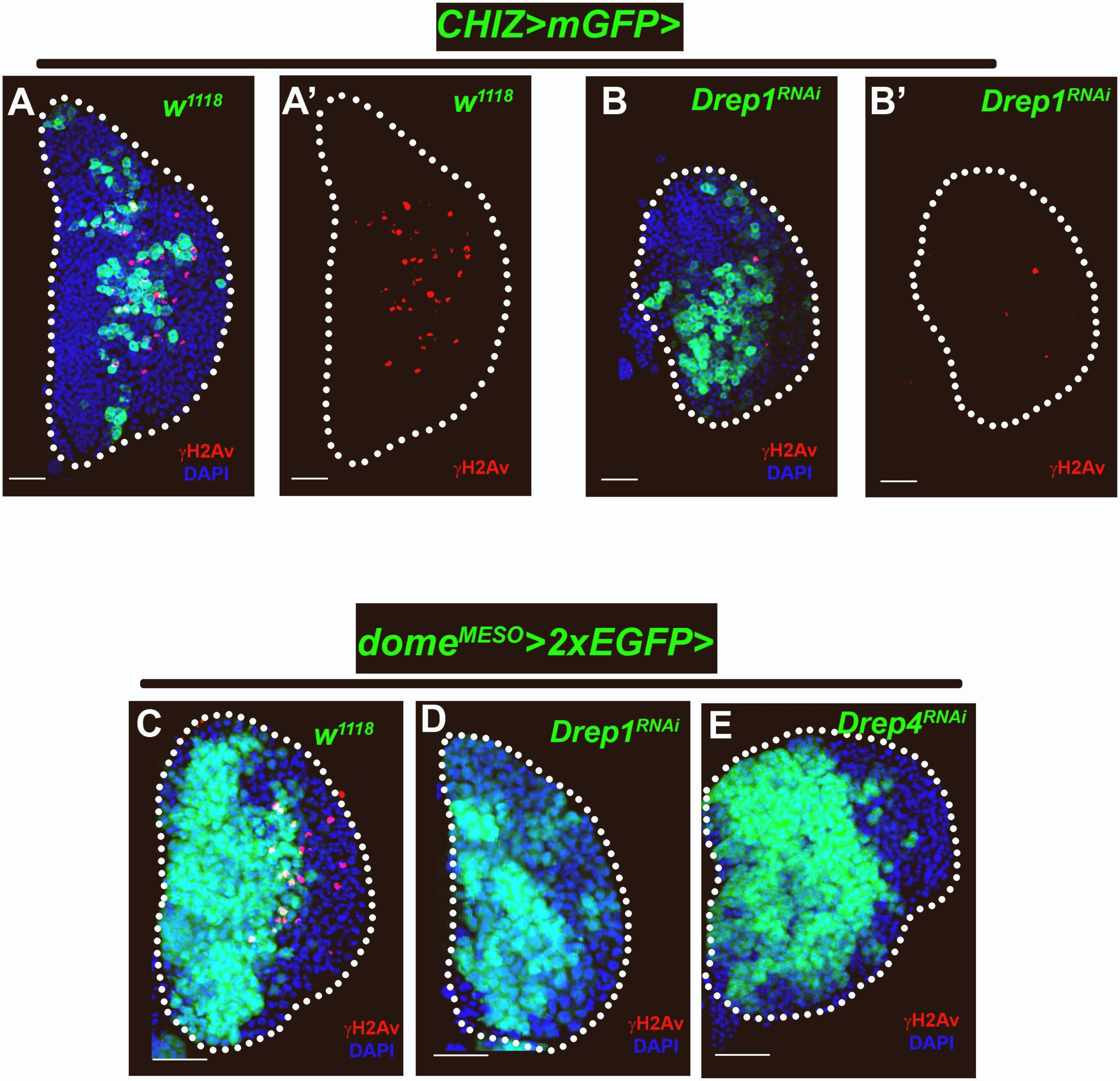

CHIZ>mGFP>
A
w^{1118}
γH2Av
DAPI
A'
w^{1118}
γH2Av
B
$Drep1^{RNAi}$
γH2Av
DAPI
B'
$Drep1^{RNAi}$
γH2Av
$dome^{MESO}$>2xEGFP>
C
w^{1118}
γH2Av
DAPI
D
$Drep1^{RNAi}$
γH2Av
DAPI
E
$Drep4^{RNAi}$
γH2Av
DAPI

Figure S4. Caspase Activated DNase induces DNA breaks required for macrophage differentiation

(A-B') Depletion of *Drosophila* ICAD [(*CHIZ>mGFP; UAS-Drep1*^{RNAi} (BL65944 as 2nd RNAi line) (B-B') in the intermediate progenitors causes less γH2Av positive cells (red), GFP cell marks intermediate progenitors (green) compared to control *CHIZ>mGFP/*+ (A-A').

(C-E) Depletion of *Drosophila* ICAD (*dome*^{MESO}*-Gal4, UAS-2xEGFP; UAS-Drep1*^{RNAi} *VDRC8357*) (D) and CAD (*dome*^{MESO}*-Gal4, UAS-2xEGFP*; *UAS-Drep4*^{RNAi}) (E) in the progenitors leads to significantly less γH2Av positive cells (red) in the lymph glands compared to control, *dome*^{MESO}*-Gal4, UAS-2xEGFP*/+ (C). GFP cell marks progenitors (green).

All images are from wandering third instar lymph gland lobes and show a 25µm scale bar and maximum intensity projections of the middle third optical section. Nuclei stained with DAPI (blue). The lymph glands boundary is demarcated by a white dotted line for clarity.

Figure S5

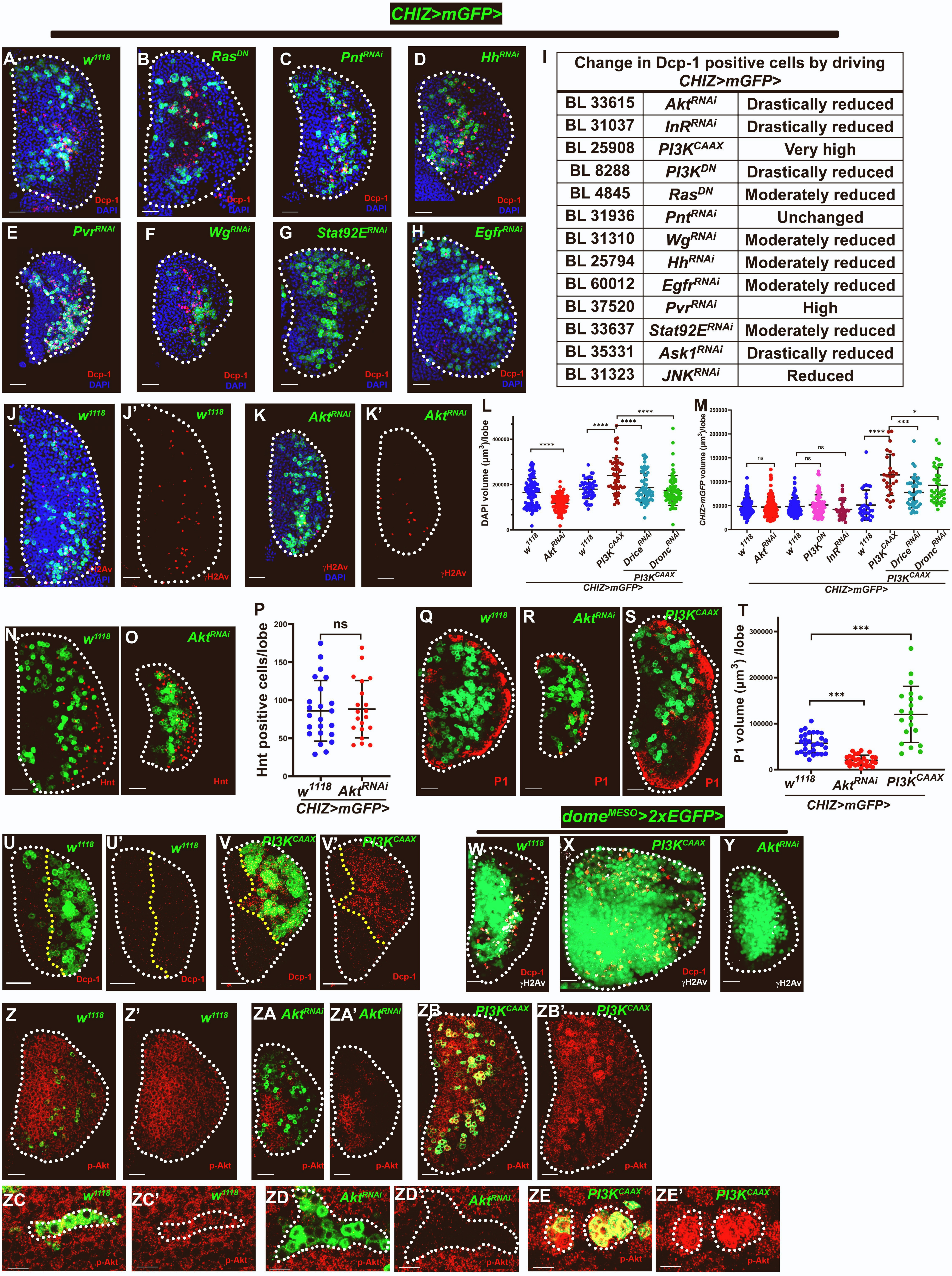

Change in Dcp-1 positive cells by driving *CHIZ>mGFP>*		
BL 33615	*Akt*^{RNAi}	Drastically reduced
BL 31037	*InR*^{RNAi}	Drastically reduced
BL 25908	*PI3K*^{CAAX}	Very high
BL 8288	*PI3K*^{DN}	Drastically reduced
BL 4845	*Ras*^{DN}	Moderately reduced
BL 31936	*Pnt*^{RNAi}	Unchanged
BL 31310	*Wg*^{RNAi}	Moderately reduced
BL 25794	*Hh*^{RNAi}	Moderately reduced
BL 60012	*Egfr*^{RNAi}	Moderately reduced
BL 37520	*Pvr*^{RNAi}	High
BL 33637	*Stat92E*^{RNAi}	Moderately reduced
BL 35331	*Ask1*^{RNAi}	Drastically reduced
BL 31323	*JNK*^{RNAi}	Reduced

Figure S5. InR/PI3K/Akt signaling regulates caspase activity and DNA breaks in macrophage differentiation

(A-I) Screening of several signaling pathways to check the involvement of caspase regulation in the lymph gland: knockdown of genes in the differentiating progenitors using *CHIZ>mGFP* (green) driver, *UAS-Ras*^{DN} (B), *UAS-Pnt*^{RNAi} (C), *UAS-Hh*^{RNAi} (D)*, UAS-Pvr*^{RNAi} (E)*, UAS-Wg*^{RNAi} (F)*, UAS-stat92E*^{RNAi} (G) *and UAS-Egfr*^{RNAi} (H) show change in the Dcp-1 positive cells mention in (I) as compared to control (*CHIZ>mGF*P/+) (A).

(J-K') *CHIZ>mGFP* (green) driven *Akt*^{RNAi} (BL 31701 as 2nd RNAi line) (K-K') has fewer H2Av-positive cells (red) than the control (J-J').

(L) Quantification of lymph gland size by measuring the DAPI volume in control sets, *CHIZ>mGFP/+* (96) and significantly reduced in *Akt*^{RNAi} (n=102), control *CHIZ>mGFP/+* (n=50) significantly high in *PI3K*^{CAAX} (n=58), and rescued in *PI3K*^{CAAX}*; Drice*^{RNAi} (n=63) and *PI3K*^{CAAX}; *Dronc*^{RNAi} (n=69).

(M) Quantification of the GFP positive cells volume in several control sets, *CHIZ>mGFP/+* (122) and *CHIZ>mGFP* driven *Akt*^{RNAi} (n=119), control *CHIZ>mGFP/+* (n=83) and *PI3K*^{DN} (n=78), *InR*^{RNAi} (n=34) show no change in GFP+ cells volume. However, *CHIZ>mGFP* driven *PI3K*^{CAAX} (n=28) show high GFP+ cells' volume and partially rescued in *PI3K*^{CAAX}*; Drice*^{RNAi} (n=41) and *PI3K*^{CAAX}; *Dronc*^{RNAi} (n=38) compared to control *CHIZ>mGFP/+* (n=30) group.

(N-O) *CHIZ>mGFP* driven *Akt*^{RNAi} (n=20) (O) do not show the difference in crystal cells (Hnt staining in red) number compared to in control, *CHIZ>mGFP/+* (n=23) (N).

(P) Quantification of the Hnt positive cell number in (N-O).

(Q-S) *CHIZ>mGFP*-driven *Akt*^{RNAi} (n=28) (R) shows less P1 (red), a macrophage marker, and *CHIZ>mGFP* driven *PI3K*^{CAAX} (n=20) (S) shows high P1 as compared to control, *CHIZ>mGFP/+* (n=22) (Q).

(T) Quantification of the P1 volume in (Q-S).

(U-V') Dcp-1 staining (red) in control, *CHIZ>mGFP/+* (U-U'), and CHIZ>mGFP driven PI3KCAAX (V-V') shows high Dcp-1 staining in early third-instar larvae (48 hours after hatching at 29°C) in CHIZ+ cells.

(W-Y) γH2Av (grey) and Dcp-1 (red) co-staining in control *dome*^{MESO}*-Gal4, UAS-2xEGFP/+* (W) and *dome*^{MESO}*-Gal4, UAS-2xEGFP/+* driven *PI3K*^{CAAX} (W) where high number of γH2Av and Dcp-1 positive cells found only in low GFP cells, whereas *dome*^{MESO}*-Gal4, UAS-2xEGFP/+* driven *Akt*^{RNAi} (Y) show less γH2Av positive cells.

(Z-ZB') Lymph gland exhibits active Akt signaling shown by p-Akt staining (red) in control, *CHIZ>mGFP/+* (Z-Z') (green), but *CHIZ>mGFP* driven *Akt*^{RNAi} (ZA-ZA'), show less p-Akt staining, and *PI3K*^{CAAX} (ZB-ZB') show high p-Akt staining.

(ZC-ZE') High magnification images of p-AKT staining (red) in control, *CHIZ>mGFP/+* (ZC-ZC'), and *CHIZ>mGFP* driven *Akt*^{RNAi} (ZD-ZD') shows less staining in CHIZ^{+} cells, and *PI3K*^{CAAX} (ZE-ZE') shows high staining in CHIZ^{+} cells.

All images are from wandering third instar lymph gland lobes except image U-V', the early third instar lymph gland lobe. Scale bars: 25µm in all images except 10µm in (ZC-ZE') with maximum intensity projections of the middle third optical sections except image Z-ZE', which are single optical sections of the lymph glands. Control groups are different for their respective experimental sets because experiments are performed on different days. Nuclei stained with DAPI (blue). The lymph glands boundary is demarcated by a white dotted line for clarity. *P < 0.05 ***P < 0.001, ****P < 0.0001, ns- not significant. Error bars, mean ± SD. All images are representative of 3 or more independent biological experiments, and 'n' represents the number of lymph gland lobes.

Figure S6

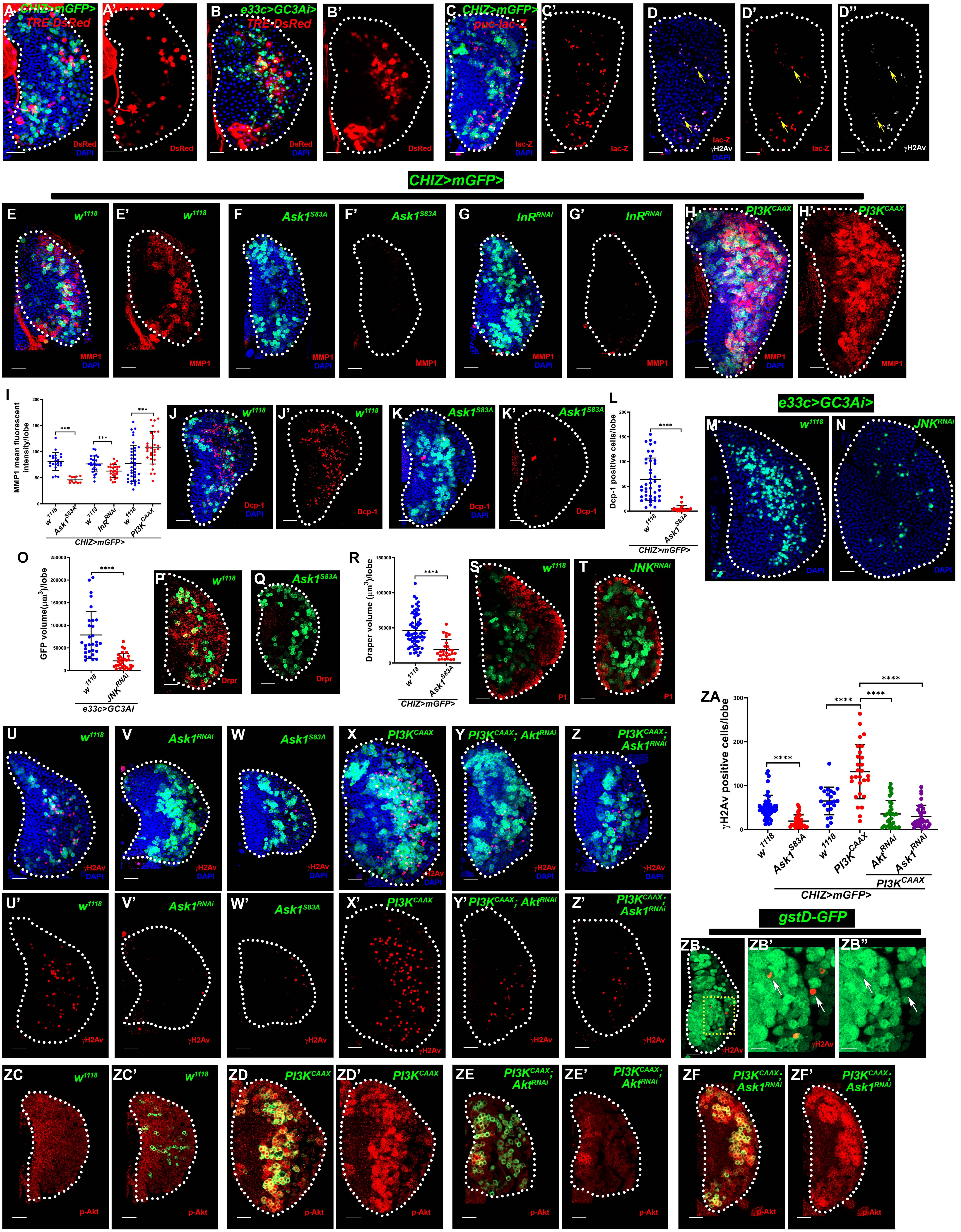
CHIZ>mGFP>
TRE-DsRed
e33c>GC3Ai>
puc-lac-Z
DsRed
DAPI
lac-Z
γH2Av
MMP1
w1118
Ask1S83A
InRRNAi
PI3KCAAX
MMP1 mean fluorescent intensity/lobe
Dcp-1
Dcp-1 positive cells/lobe
JNKRNAi
GFP volume(μm3)/lobe
Drpr
Draper volume (μm3)/lobe
P1
Ask1RNAi
PI3KCAAX; AktRNAi
PI3KCAAX; Ask1RNAi
γH2Av positive cells/lobe
gstD-GFP
p-Akt

Figure S6. InR/PI3K/Akt signaling via Ask1/JNK axis regulate caspase activity

(A-A') JNK signaling activity reporter *TRE-DsRed*-positive (red) cells found in the differentiating progenitors (green) (*CHIZ>mGFP/+*; *TRE-DsRed/+*).

(B-B') *TRE-DsRed*-positive cells colocalized with the caspase active cells (GC3Ai) (green) in the intermediate zone of the lymph gland (*UAS-GC3Ai/+; e33c-Gal4/ TRE-DsRed*).

(C-D'') The JNK signaling activity reporter puc-lacZ (pucE69) shows JNK signaling activity (red) in the differentiating progenitors (green) (CHIZ>mGFP/+; pucE69/+), which is colocalized with γH2Av-positive cells(D-D').

(E-H') JNK signaling activity using MMP1 staining (red) in *CHIZ>mGFP* (green) driven *Ask1*^{S83A} (n=12) (F-F') and *InR*^{RNAi} (n=27) (G-G') were severely reduced and drastically high in *PI3K*^{CAAX} (n=30) (H-H') as compared to their respective control sets, *CHIZ>mGFP/+* (n=23, n=26, and n=38) (E-E').

(I) Quantification of mean fluorescent intensity of MMP1 in (E-H').

(J-K') *CHIZ>mGFP* driven *Ask1*^{S83A} (n=38) (K-K') shows Dcp-1-positive cells (red) significantly decrease compared to the control, *CHIZ>mGFP/+* (n=42) (J-J').

(L) Quantification of Dcp-1-positive cells in (J-K').

(M-N) Loss of JNK in the lymph gland (*UAS-GC3Ai/+; e33c-Gal4/UAS-JNK*^{RNAi}) (n=30) (N) shows severely reduced caspase-active (GC3Ai) cells compared to control, (*UAS-GC3Ai/+; e33c-Gal4/+*) (n=28) (M).

(O) Quantification of Caspase-positive cells GC3Ai volume in (M-N).

(P-Q) Draper staining (red) in *CHIZ>mGFP* driven *Ask1*^{S83A} (n=41) (Q) significantly decreased compared to control, *CHIZ>mGFP/+* (n=64) (P).

(R) Quantification of Draper volume in (P-Q).

(S-T) P1 staining (red) in *CHIZ>mGFP* driven *JNK*^{RNAi} (T) significantly decrease compared to control, *CHIZ>mGFP/+* (S).

(U-Z') *CHIZ>mGFP* driven *Ask1*^{RNAi} (BL32646 as 2nd RNAi line) (V-V') and *JNK1*^{RNAi} (n=21) (W-W') show reduced γH2Av-positive cells (red) as compared to respective control, *CHIZ>mGFP/+* (n=47) (U-U'). Depletion of Akt [*CHIZ>mGFP; UAS-PI3K*^{CAAX}*; UAS- Akt*^{RNAi} (n=35)] (Y-Y') and Ask1 [*CHIZ>mGFP; UAS-PI3K*^{CAAX}*; UAS- Ask1*^{RNAi} (n=28) (Z-Z')] in intermediate progenitors [control *CHIZ>mGFP/+* (n=38) (U-U')] rescue the high γH2Av-positive cells (red) phenotype of *PI3K*^{CAAX} overexpression [*CHIZ>mGFP; UAS-PI3K*^{CAAX} (n=35)] (X-X').

(ZB-ZB'') γH2Av (red) positive cells in the lymph gland display low *gstD-GFP/+* (green) (ZB), and high magnification images (ZB'-ZB'') show γH2Av positive cells in low gstD-GFP (ROS marker) cells indicated by an arrow.

(ZC-ZF') Depletion of Akt (*CHIZ>mGFP; UAS-PI3K*^{CAAX}*; UAS- Akt*^{RNAi}) (ZE-ZE') in intermediate progenitors (control *CHIZ>mGFP/+*) (ZC-ZC') reduces the high p-Akt staining (red) phenotype of *PI3K*^{CAAX} overexpression (*CHIZ>mGFP; UAS-PI3K*^{CAAX}) (ZD-ZD') however the depletion of Ask1 (*CHIZ>mGFP; UAS-PI3K*^{CAAX}*; UAS-Ask1*^{RNAi}) (ZF-ZF') fail to reduce the high p-Akt staining (red) phenotype of *PI3K*^{CAAX} overexpression and remain same as *PI3K*^{CAAX} overexpression [*CHIZ>mGFP; UAS-PI3K*^{CAAX}] (ZD-ZD').

All images from the wandering third instar lymph gland lobe show a 25µm scale bar except image (ZB'-ZB''), where the scale bar is 10µm and maximum-intensity projections of the middle third optical sections except images (B-B', D-D'' and ZB-ZF'), which are single optical

sections of the lymph glands. Nuclei stained with DAPI (blue). Control groups are different for their respective experimental sets because experiments are performed on different days. Nuclei stained with DAPI (blue). The lymph glands boundary is demarcated by a white dotted line for clarity. ***P < 0.001, ****P < 0.0001, ns- not significant. Error bars, mean ± SD. All images are representative of 3 or more independent biological experiments, and ‘n’ represents the number of lymph gland lobes.

Figure S7

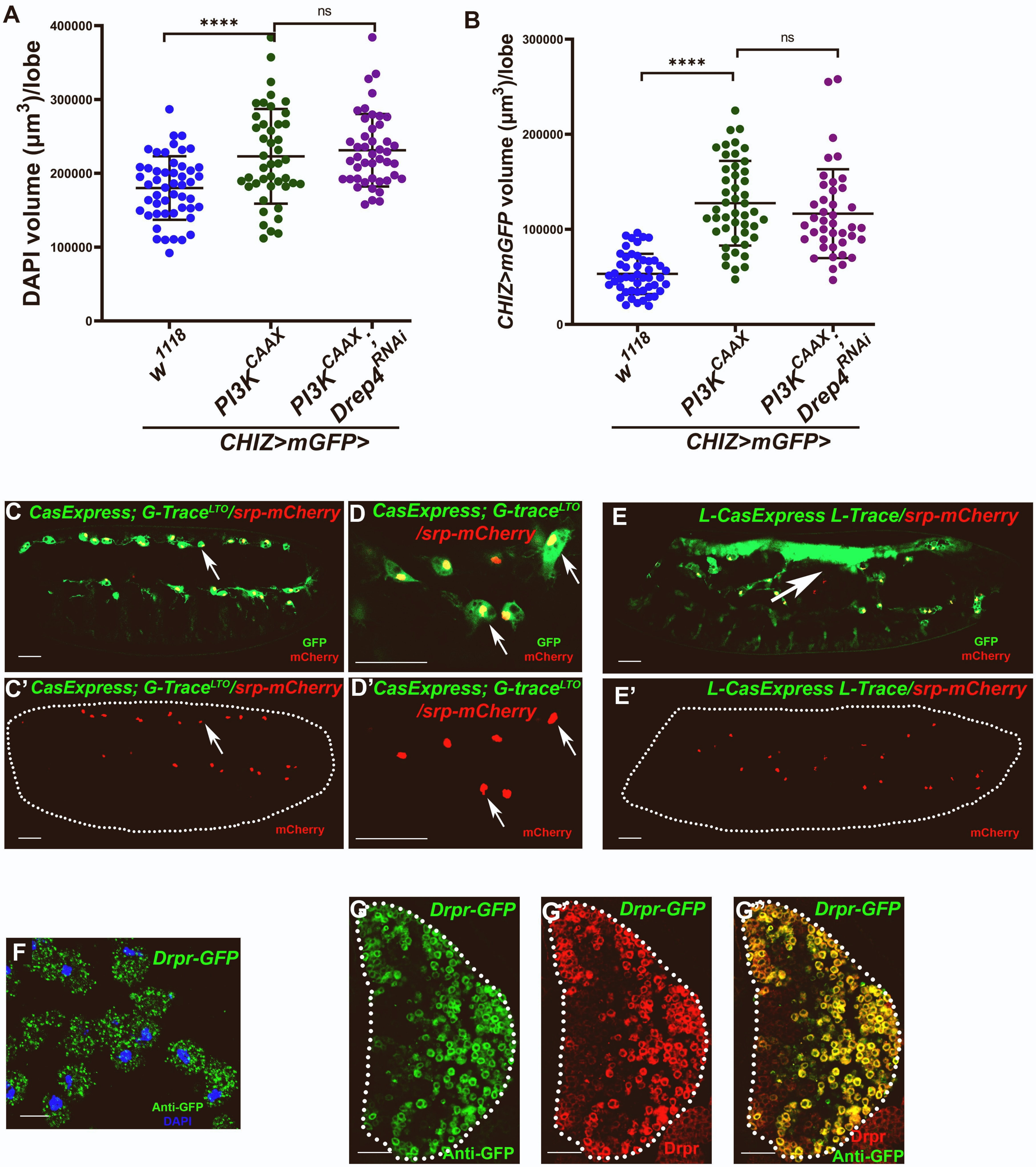
A
DAPI volume (µm3)/lobe
400000
300000
200000
100000
0
****
ns
w1118
PI3KCAAX
PI3KCAAX ;
Drep4RNAi
CHIZ>mGFP>
B
CHIZ>mGFP volume (µm3)/lobe
300000
200000
100000
0
****
ns
w1118
PI3KCAAX
PI3KCAAX ;
Drep4RNAi
CHIZ>mGFP>
C CasExpress; G-TraceLTO/srp-mCherry
GFP
mCherry
D CasExpress; G-traceLTO
/srp-mCherry
GFP
mCherry
E L-CasExpress L-Trace/srp-mCherry
GFP
mCherry
C' CasExpress; G-TraceLTO/srp-mCherry
mCherry
D' CasExpress; G-traceLTO
/srp-mCherry
mCherry
E' L-CasExpress L-Trace/srp-mCherry
mCherry
F Drpr-GFP
Anti-GFP
DAPI
G Drpr-GFP
Anti-GFP
G' Drpr-GFP
Drpr
G'' Drpr-GFP
Drpr
Anti-GFP

Figure S7. Developmental PI3K/Akt signaling regulates caspase/CAD activation for phagocytic macrophage differentiation.

(A) Quantification of DAPI volume in control, *CHIZ>mGFP/+* (n=50), and *CHIZ>mGFP* driven *UAS-PI3K*^{CAAX} (n=44) have significantly high GFP volumes, which remain high in rescue *UAS-PI3K*^{CAAX}*; UAS-Drep4*^{RNAi} (n=45).

(B) Quantification of GFP volume in control, *CHIZ>mGFP/+* (n=49) and *CHIZ>mGFP* driven *UAS-PI3K*^{CAAX} (n=47) have significantly high GFP volume, which remains high in rescue *UAS-PI3K*^{CAAX}*; UAS-Drep4*^{RNAi} (n=41).

(C-D') Embryonic macrophages (stage 13) marked by *srp-mCherry* show experience of caspase lineage activity (*CasExpress; G-Trace*^{LTO}) (C-C') also shows in high magnification (D-D'), marked by arrows.

(E-E') Embryonic macrophages (stage 13) marked by *srp-mCherry* experience caspase lineage *L-CasExpress L-Trace* (*lex-Aop-Flp::Ubi-FRT-STOP-FRT-GFP/+; L-caspase/+*) GFP (green) dorsal closure (marked by arrow) and macrophages.

(F) Circulating blood cells of third instar *Draper-GFP* larvae show Draper (green) punctate staining.

(G-G'') *Draper-GFP* expression using anti-GFP (green) (G) and staining of anti-Draper (red) antibody (G') and merged image of both (G'') of 3rd instar larval lymph gland.

All images show a 25µm scale bar except image F, where the scale bar is 10µm. All images are single optical sections. Nuclei stained with DAPI (blue). For clarity, the lymph gland and embryo boundary are demarcated by a white dotted line. All images are representative of 3 or more independent biological experiments, and 'n' represents the number of lymph gland lobes. ****P < 0.0001, ns- not significant. Error bars, mean ± SD

Also see Movies S1, S2, and S3.
