# Supplementary material for: Transient caspase-mediated activation of caspase-activated DNase causes DNA damage required for phagocytic macrophage differentiation
Source: Cell Rep. Author manuscript; Available in PMC 2025 Jan 9. (PMC7617294; doi:10.1016/j.celrep.2024.114251)
Supplement: Supplementary Information [file EMS202103-supplement-Supplementary_Information.zip › 1-s2.0-S2211124724005795-mmc6.pdf]

Cell Reports

Article

Transient caspase-mediated activation of caspase-activated DNase causes DNA damage required for phagocytic macrophage differentiation

Graphical abstract

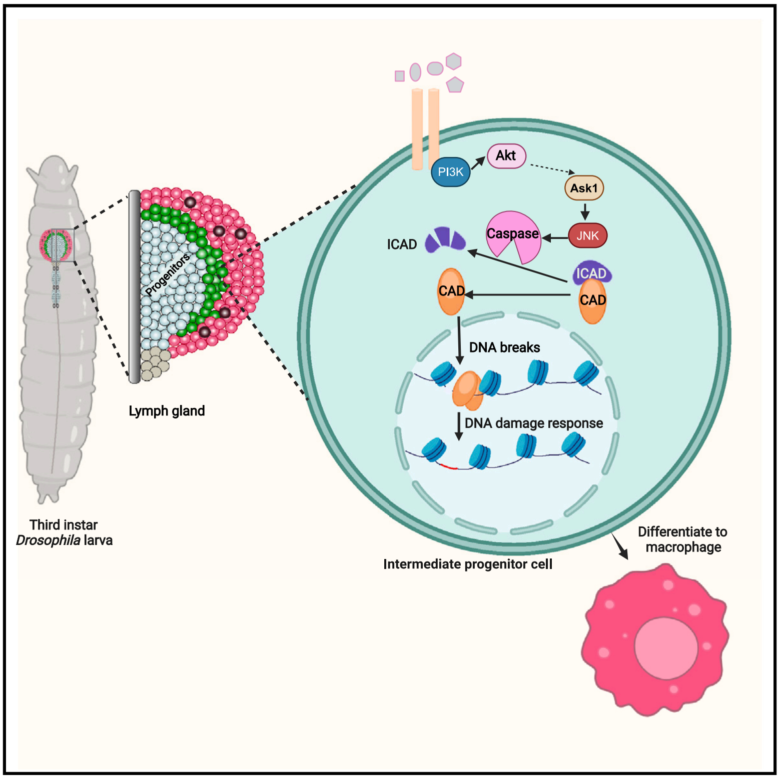

Highlights

- DNA strand breaks during macrophage differentiation in the *Drosophila* lymph gland
- Caspase signaling cascade activation is what causes these DNA breaks
- Caspase-activated DNase (CAD) is responsible for inducing the DNA breaks
- Akt signaling via JNK regulates sublethal caspase activity during macrophage differentiation

Authors

Deepak Maurya, Gayatri Rai,
Debleena Mandal, Bama Charan Mondal

Correspondence

bamacharan@bhu.ac.in

In brief

Mechanisms of versatile and self-renewing phagocytic macrophage differentiation remain unclear. Maurya et al. show that the PI3K/Akt signaling through the ROS/Ask1/JNK axis regulates the sublethal level of caspases and caspase-activated DNase (CAD) activation, causing DNA strand breaks to be required for *Drosophila* macrophage differentiation.

Maurya et al., 2024, Cell Reports *43*, 114251

May 28, 2024 © 2024 The Authors. Published by Elsevier Inc.
https://doi.org/10.1016/j.celrep.2024.114251

CellPress

Cell Reports

CellPress
OPEN ACCESS

Article

Transient caspase-mediated activation of caspase-activated DNase causes DNA damage required for phagocytic macrophage differentiation

Deepak Maurya,[1] Gayatri Rai,[1] Debleena Mandal,[1] and Bama Charan Mondal[1,2,*]
[1]Cytogenetics Laboratory, Department of Zoology, Institute of Science, Banaras Hindu University, Varanasi 221005, India
[2]Lead contact
*Correspondence: bamacharan@bhu.ac.in
https://doi.org/10.1016/j.celrep.2024.114251

SUMMARY

Phagocytic macrophages are crucial for innate immunity and tissue homeostasis. Most tissue-resident macrophages develop from embryonic precursors that populate every organ before birth to lifelong self-renew. However, the mechanisms for versatile macrophage differentiation remain unknown. Here, we use *in vivo* genetic and cell biological analysis of the *Drosophila* larval hematopoietic organ, the lymph gland that produces macrophages. We show that the developmentally regulated transient activation of caspase-activated DNase (CAD)-mediated DNA strand breaks in intermediate progenitors is essential for macrophage differentiation. Insulin receptor-mediated PI3K/Akt signaling regulates the apoptosis signal-regulating kinase 1 (Ask1)/c-Jun kinase (JNK) axis to control sublethal levels of caspase activation, causing DNA strand breaks during macrophage development. Furthermore, caspase activity is also required for embryonic-origin macrophage development and efficient phagocytosis. Our study provides insights into developmental signaling and CAD-mediated DNA strand breaks associated with multifunctional and heterogeneous macrophage differentiation.

INTRODUCTION

Macrophages are evolutionarily conserved phagocytic cells with crucial roles in innate immunity, development, tissue-specific function, and monitoring of aberrant cells like cancer cells.[1–3] Fate-mapping, single-cell transcriptomics, and epigenetic studies showed that these heterogeneous tissue-resident macrophages arise from early embryonic (yolk sac and fetal liver) erythro-myeloid progenitors and reside lifelong with limited self-renewal.[4–7] However, in some tissues like the intestine, bone marrow-derived circulating monocytes differentiate into tissue-specific macrophages when needed.[8] Myeloid progenitor differentiation requires precise control of gene expression, which is regulated by transcription factors, chromatin landscape, cellular metabolism, autophagy, apoptotic factors, and systemic cues during development and disease.[9–14]

Apoptotic signaling activates protease caspases that target hundreds of proteins during cell death.[15] However, studies have shown that active caspases are also required during various types of cell differentiation across species,[16–18] including mammalian myeloid cell development, such as the development of erythrocytes, platelets, and monocyte-to-macrophage differentiation. In *ex vivo* culture, colony-stimulating factor-1 (CSF-1)-mediated monocyte-to-macrophage differentiation is associated with caspase-3 activation.[13] Caspase-8 deletion in mouse bone marrow cells also inhibits monocyte differentiation into macrophages.[13] However, the precise mechanism responsible for macrophage differentiation remains unknown.

To investigate the mechanisms underlying macrophage heterogeneity and versatility, we used *in vivo* genetic analysis of the *Drosophila* hematopoietic system, which only has myeloid-type blood cells. *Drosophila* hematopoiesis uses evolutionarily conserved transcription factors (e.g., GATA factor Serpent, Runx factor Lozenge) and signaling pathways (e.g., Notch, JAK/STAT, Toll signaling) in development and innate immunity.[19,20] As in mammals, *Drosophila* hematopoiesis also occurs in two waves. The first-wave blood cells (hemocytes) develop in the early embryo's head mesoderm, contributing to embryonic, larval, and adult stages. The second wave in the cardiogenic region at the late embryonic stage generates the larval hematopoietic organ, the lymph gland, which includes a niche, multipotent progenitors, intermediate progenitors or differentiating cells, and differentiated cells (Figure 1A).[19–21] The lymph gland's blood progenitors in the inner core proliferate during the early larval stages. At mid-second instar, they stop dividing and differentiate into plasmatocytes and crystal cells at the lymph gland's outer boundary's distal margins, which disperse during pupation and make adult blood cells. Most *Drosophila* blood cells are macrophage-like cells called plasmatocytes (hereafter referred to as macrophages). Like mammalian macrophages, *Drosophila* macrophages phagocytose pathogens and apoptotic cells, produce anti-microbial peptides and inflammatory mediators, help to repair and regenerate tissue, maintain metabolic homeostasis, and transdifferentiate into other hemocytes.[22,23] Recent studies using enhancer analysis[24] and single-cell transcriptomics suggest vertebrate-like heterogeneous tissue-specific macrophages in *Drosophila* larvae and adults.[22,25]

This is an open access article under the CC BY license (http://creativecommons.org/licenses/by/4.0/).

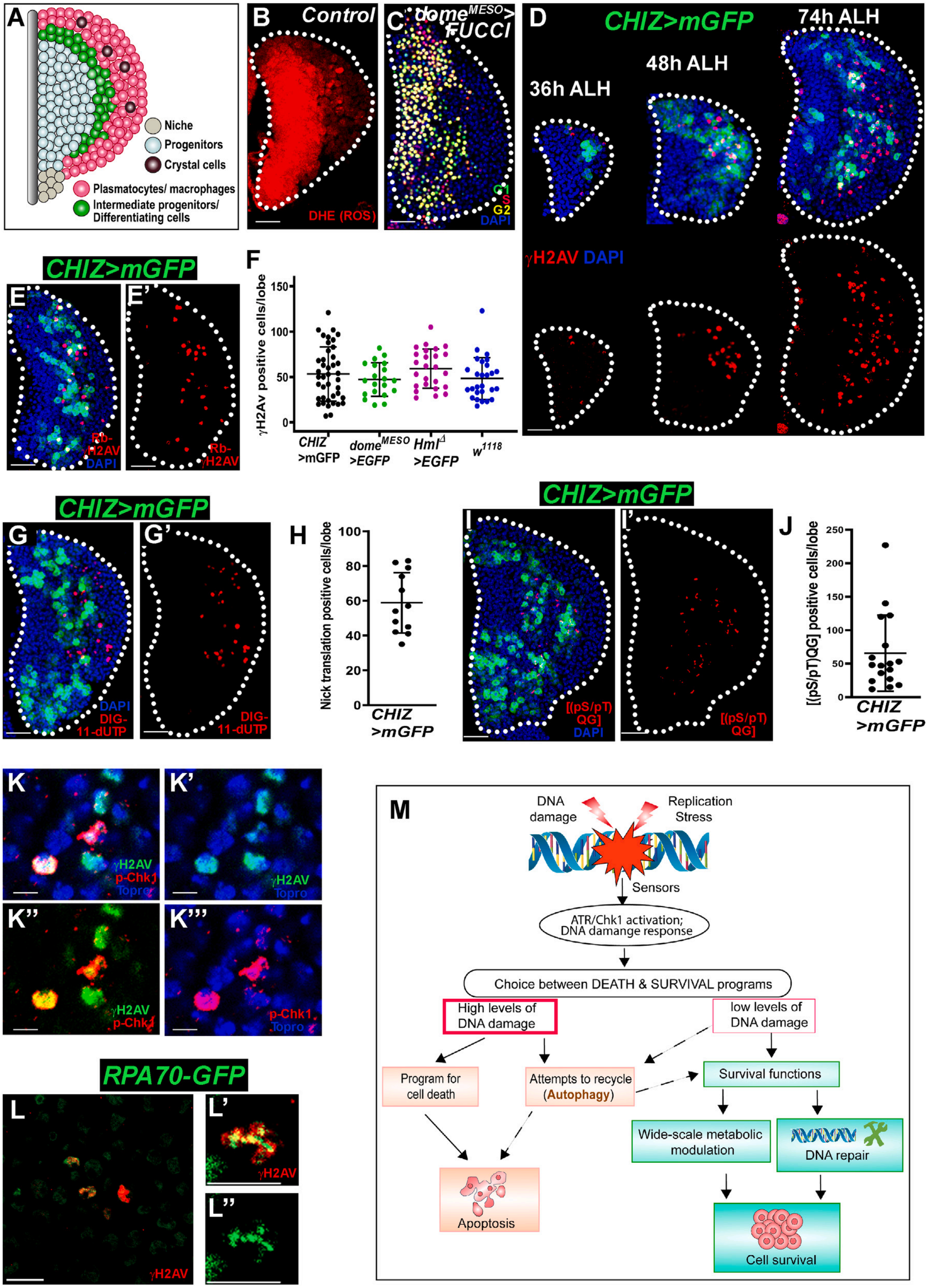

(legend on next page)

Local microenvironmental signals,[26] cell-autonomous factors downstream of platelet-derived growth factor (PDGF)/vascular endothelial growth factor (VEGF) receptor (Pvr), Wnt6, EGFR, and JAK/STAT signalings,[27–31] or systemic signals (e.g., insulin receptor [InR], GABA-R)[32–34] regulate lymph gland progenitor maintenance. Besides, the third-instar lymph gland progenitors show a high level of reactive oxygen species (ROS)[35] (Figure 1B), like mammalian common myeloid progenitors, but have a lengthy G2 cell cycle phase (Figure 1C). Interestingly, stress-mediated DNA breakage triggers the DDR, resulting in G2 arrest until the damage is adequately repaired or apoptosis occurs.[36]

Here, we show that sublethal apoptotic caspases activate caspase-activated DNase (CAD), triggering DNA damage in *Drosophila* lymph gland intermediate progenitors during the normal development of macrophages. We find that insulin-receptor-mediated PI3K/Akt signaling in differentiating macrophages induces sublethal caspase activation potentially through the ROS/apoptosis signal-regulating kinase 1 (Ask1)/c-Jun kinase (JNK) axis. Furthermore, caspase activity is required during embryonic-origin macrophage development for efficient phagocytosis. This study thus reveals that developmental signaling and caspase-activated DNA breaks are involved in macrophage differentiation.

RESULTS

DNA breaks occur during myeloid-type progenitor cell differentiation in lymph glands

We first investigated whether developmentally controlled G2 arrest in the third-instar lymph gland progenitors (Figure 1C)[30] is due to DNA damage. We monitored the status of DNA damage response (DDR) marker-positive cells using a mouse anti-γH2Av (γH2AX homolog) antibody[37] during larval development in the lymph gland cells with appropriate negative and positive controls (Figures S1A–S1C′, S1I, and S1I′).[38,39] Interestingly, we found that γH2Av-positive cells appear in the periphery of the lymph gland at the mid-second instar (36 h after larval hatching [ALH]), which coincides with the onset of differentiation[28] (Figure 1D). The γH2Av-positive cell numbers increase in the differentiating zone during the early third instar (48 h ALH), and this number further increases in the wandering third-instar-stage lymph gland (74 h ALH) (Figure 1D).

Differentiating cells or intermediate progenitors co-express the progenitor marker *dome*^{MESO} and the earliest differentiating cell marker *Hml*.[40] Using the *split-Gal4* strategy, a driver, *CHIZ-Gal4 UAS-mCD8::GFP* (hereafter *CHIZ>mGFP* genotype or CHIZ^{+} cells), was made[40] (green cells in Figures 1A and 1D) that could mark most of the differentiating or intermediate progenitor zone. Similar to Figure 1D, γH2Av-positive cells were also found by using another widely used rabbit anti-γH2Av antibody[41] in the differentiating zone (CHIZ^{+}) (Figures 1E and 1E′). γH2Av-positive cells were negative for mature macrophage marker P1 (also called NimC1) (Figures S1D–S1D″). Notably, γH2Av staining covered the entire nuclear region, except the DAPI-bright heterochromatin region (Figures S1E–S1E″).

This important finding of the connection between differentiating cells and DDR was confirmed in multiple genotypes using several methods. We assessed γH2Av-positive cell numbers in fly lines used to study *Drosophila* hematopoiesis, such as *w*^{1118}; *CHIZ>mGFP*; *Hml*$^{\Delta}$*-Gal4*, *UAS-2xEGFP*; and *dome*^{MESO}*-Gal4*, *UAS-2xEGFP*, to rule out the genetic background effect. Indeed, the third-instar lymph glands across genotypes had similar γH2Av-positive cell numbers in the differentiating zone (Figures 1F and S1F–S1H). We evaluated DNA breakage with an *in vivo* nick translation assay with proper controls (Figures S1L–S1O′) and found a similar number of digoxigenin (DIG)-labeled dUTP-incorporated nuclei, indicating DNA repair synthesis,[42] in comparison to γH2Av-labeled nuclei in the intermediate zone (Figures 1G, 1H, S1J–S1K, and 1F). DNA damage activates ATR/ATM kinases, phosphorylating H2Av, Chk1, and other DDR proteins.[36] Immunostaining of lymph glands for the phospho-ATM/ATR substrate motif ((pS/pT)QG)[41] showed a pattern similar to the γH2Av-positive cells (Figures 1I and 1J). Phospho-Chk1, a well-known DDR marker,[43] co-localized with γH2Av-positive cells

Figure 1. DNA damage occurs during the differentiation of the lymph gland

(A) Schematic showing different cell type locations in the third-instar primary lymph gland lobe.

(B) Dihydroethidium staining (red) in lymph gland progenitors *dome*^{MESO}*-Gal4, UAS-2xEGFP/+* (without GFP channel) displays high ROS.

(C) Lymph gland progenitors (*dome*^{MESO}*-Gal4*, *UAS-FUCCI*) mostly arrested in the G2 cell cycle phase (yellow) .

(D) Control lymph glands (*CHIZ-Gal4 UAS-mGFP/+*) at 36, 48, and 74 h after larval hatching (ALH) show that the DDR marker mouse anti-γH2Av-positive cell (red) number increased with larval age in the intermediate zone marked *CHIZ>mGFP* (green).

(E and E′) Rabbit anti-γH2Av immunostaining (red) in *CHIZ>mGFP* (green) (E) and only γH2Av (E′) matches with (D), 74 h ALH.

(F) γH2Av-positive cell quantification in different genotypes: *CHIZ-Gal4, UAS-mGFP/+* (n = 41); *dome*^{MESO}*-Gal4, UAS-2xEGFP/+* (n = 20); *Hml*$^{\Delta}$*-Gal4, UAS-2xEGFP/+* (n = 25); and *w*^{1118} (n = 27) per lymph gland lobe (shown in Figure S1).

(G and G′) Nick translation (red) shows incorporation of DIG-11-dUTP in control lymph gland's intermediate progenitor zone *CHIZ>mGFP/+* (n = 12), indicating DNA strand breaks.

(H) Quantification of nick translation-positive cells in (G) and (G′).

(I and I′) In control lymph gland intermediate zone (*CHIZ>mGFP/+*, n = 17), mark anti-phospho-ATM/ATR substrate motif ([pS/pT]QG) (red), indicating ATM/ATR activity.

(J) Quantification of ATM/ATR substrate motif-positive cells in (I) and (I′).

(K–K‴) Magnified image from lymph gland showing DDR marker p-Chk1 (red) co-localizes with γH2Av (green) and Topro3 nuclei (blue).

(L–L″) *RPA70-GFP* (high intensity) co-localizes with γH2Av-positive cells (red).

(M) A schematic showing the choice between cell death and survival upon DNA damage.

Except for image (D), which shows 36, 48, and 74 h ALH stage lymph glands, all images are from the wandering third-instar lymph gland. All scale bars represent 25 μm except (L) 10 μm and (K–K‴ and L′–L″) 5 μm, with maximum intensity projections of the middle third optical sections except (B), (C), and (K)–(L″), which are single optical sections of the lymph glands. DAPI (blue)-stained nuclei. Error bars, mean ± standard deviation (SD). All images represent 3 or more independent biological experiments, and n represents lymph gland lobe numbers.

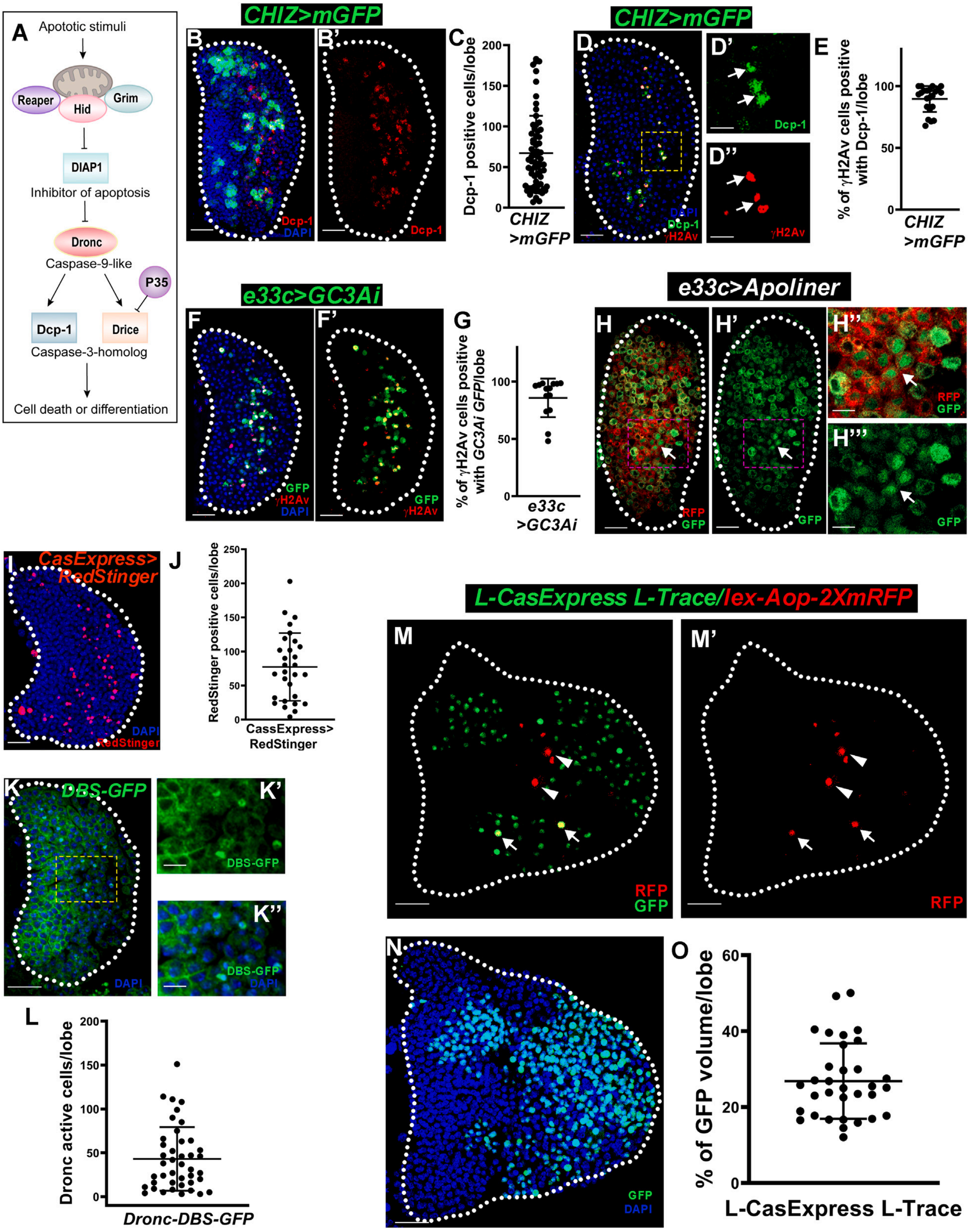

(legend on next page)

in the lymph gland (Figures 1K–1K‴). RPA1 homolog RPA70 is involved in DDR.[44] *RPA70-GFP*[45] and γH2Av immunostaining revealed high-intensity RPA70-GFP puncta co-localized with γH2Av in the lymph gland (Figures 1L–1L″, S1P, and S1P′). These findings establish that DNA damage repair foci were present in a subset of intermediate progenitors in the lymph gland.

The fluorescence ubiquitin cell cycle indicator (FUCCI) system[46] was used to evaluate the cell cycle status of DNA-damaged cells in the lymph gland. We used the *e33c-Gal4* driver to identify G1, S, G2, and M phases in the entire lymph gland and found that γH2Av-positive cells were in the G2 phase (Figures S1Q–S1Q⁗), which is further confirmed by its non-localization with PCNA-GFP[47]-positive cells, an S phase marker (Figures S1R and S1R′). However, this DNA damage is not lethal, as Nup98-GFP-marked nuclear pore complexes remained intact (Figures S1S–S1S⁗).

These findings suggest that differentiating myeloid-type blood cells have developmental DNA damage in the intermediate progenitors. Therefore, the next question that we addressed was to identify the developmental cues that cause DNA damage during myeloid-type cell differentiation.

Caspase activation and DNA breaks in differentiating myeloid-type blood cells

Cells with damaged nuclear DNA activate damage sensors, DDR, cell cycle checkpoints, and DNA repair proteins for cell survival. The strength of damage signals determines whether the cell dies (apoptosis) or survives (Figure 1M).[36] We first tested whether apoptosis pathways are activated in the third-instar lymph gland. The cleaved *Drosophila* Dcp-1 (Asp215) antibody[48] (CST, USA, cat. #9578S) detected active forms of both executioner caspases, Drice and Dcp-1 (Figure 2A).[17,49] We immunostained the third-instar lymph gland (Figures S2A–S2A″ and S2F–S2G′) with appropriate negative and positive controls. Remarkably, the lymph gland showed cleaved Dcp-1 (hereafter, Dcp-1)-positive cells in the intermediate zone (*CHIZ>mGFP/+*) (Figures 2B and 2C). However, these cells were negative for mature macrophage marker P1 (Figures S2B–S2B″). Over 90% of γH2Av-positive cells were also Dcp-1-positive cells (Figures 2D and 2E). The intermediate zones in *dome*^{MESO}*-Gal4*, *UAS-2xEGFP* and *w*^{1118} genotypes have similar caspase activity (Figures 2C and S2C–S2E), ruling out a genetic background effect.

Multiple methods confirmed the executioner caspase activity in the lymph gland. First, using *e33c-GAL4*-driven[26] *UAS-GC3Ai* and *UAS-VC3Ai*, a fluorescent executioner caspase sensor,[50] we found high caspase activity only in the differentiating zone, which co-localized with γH2Av-positive cells (Figures 2F, 2G, and S2H–S2H⁗). The Apoliner caspase reporter *UAS-Apoliner* was expressed using both *e33c-Gal4* and *CHIZ-Gal4* drivers where mRFP and GFP are initially membrane bound, but upon caspase activation, GFP translocates to the nucleus.[51] Nuclear GFP was found in the differentiating region (Figures 2H–2H‴, S2I, and S2I′). We also used *CasExpress-Gal4* (BL65420)[48] and *UAS-RedStinger* (BL8546) reporters that showed executioner caspase-positive cells in the intermediate zone (Figures 2I and 2J) but not in the mutant form *CasExpress*^{mutant}*-Gal4* (BL65419)[48] (Figure S2J). Published literature suggests that high levels of TUNEL-positive cells go to cell death.[52] We performed TUNEL staining with proper negative and positive controls (Figures S2L–S2O′) to determine if γH2Av-positive lymph gland cells were high-intensity TUNEL positive. Notably, γH2Av-positive cells lacked high-intensity TUNEL activity (Figures S2K–S2K″), but some high-intensity TUNEL-positive cells were present in the lymph gland differentiated zone. This indicates that γH2Av-positive cells are not dying. Collectively, these results suggest that executioner caspase activity is sublethal in differentiating cells.

Drosophila's active initiator caspase Dronc cleaves executioner caspases Drice and Dcp-1. We used a Drice-based sensor (DBS) line to monitor Dronc activity.[53] Interestingly, third-instar lymph glands showed nuclear-localized histone-GFP (DBS) in the intermediate zone and co-localized with γH2Av staining (Figures 2K and 2L). However, γH2Av-positive cells showed lower DBS intensity than only DBS cells (Figures S2P–S2P″). Thus, this result hints that initiator caspase Dronc is activated

Figure 2. DNA breaks and active caspase in differentiating progenitor blood cells
(A) *Drosophila* apoptotic pathway schematic.
(B and B′) Lymph gland intermediate progenitor zone (*CHIZ>mGFP/+* [green], *n* = 66) exhibits cleaved Dcp-1 (red) immunostaining.
(C) Quantification of Dcp-1-positive cells/lymph gland lobe in *CHIZ>mGFP/+* (*n* = 66) genotype.
(D–D″) In *CHIZ>mGFP/+* lymph gland (without *mGFP*), γH2Av (red) cells are also Dcp-1 positive (green) (D); inset shows Dcp-1 (D′) and γH2Av (D″).
(E) Quantification of (D)–(D″) (*n* = 22) reveals >90% γH2Av-positive cells co-localizing with Dcp-1.
(F and F′) GFP (green) fluorescent reporter of executioner caspase activity (*e33c-Gal4, UAS-GC3Ai*) co-localizes with γH2Av-positive cells (red) (*n* = 14).
(G) Quantification of (F) and (F′) showing percentage of co-localization/lymph gland lobe.
(H–H‴) Lymph gland expressing Apoliner (*e33c-Gal4*, *UAS-Apoliner*), where RFP (red) and GFP (green) colocalize at membrane, but caspase activity (arrow) causes GFP to relocalize in nuclei (H and H′) and high magnification (H″ and H‴).
(I) *CasExpress-Gal4*, *UAS-RedStinger* (*n* = 29) expression shows executioner caspase activity (red) in the lymph gland.
(J) Quantification of caspase active cells in (I).
(K–K″) Initiator caspase Dronc activity shown by nuclear *Drice-based-sensor-GFP* (*DBS-GFP*) (*n* = 42) in the lymph gland intermediate zone (K) and magnified images (K′ and K″).
(L) Quantification of *DBS-GFP* cells in (K).
(M and N) The *L-CasExpress L-Trace* (*lex-Aop-Flp::Ubi-FRT-STOP-FRT-GFP/lex-Aop-2XmRFP; L-caspase/+*) shows real-time executioner caspase activity in RFP (red) cells (arrowheads in M and M′), with caspase lineage trace cells with GFP (green) (M), co-localized cells (arrows in M and M′), and lymph gland middle third section lineage trace GFP (N).
(O) Quantification of the ratio of caspase lineage cells and DAPI volumes in (M) and (N).
All images are single optical sections except (B), (B′), (I), and (N), which are maximum intensity projections of the middle third optical section of the wandering third-instar lymph gland. All scale bars represent 25 μm except (H″ and H‴) 5 μm and (K′ and K″) 10 μm. DAPI (blue)-stained nuclei. Error bars, mean ± SD. All images represent 3 or more independent biological experiments, and *n* represents lymph gland lobe numbers.

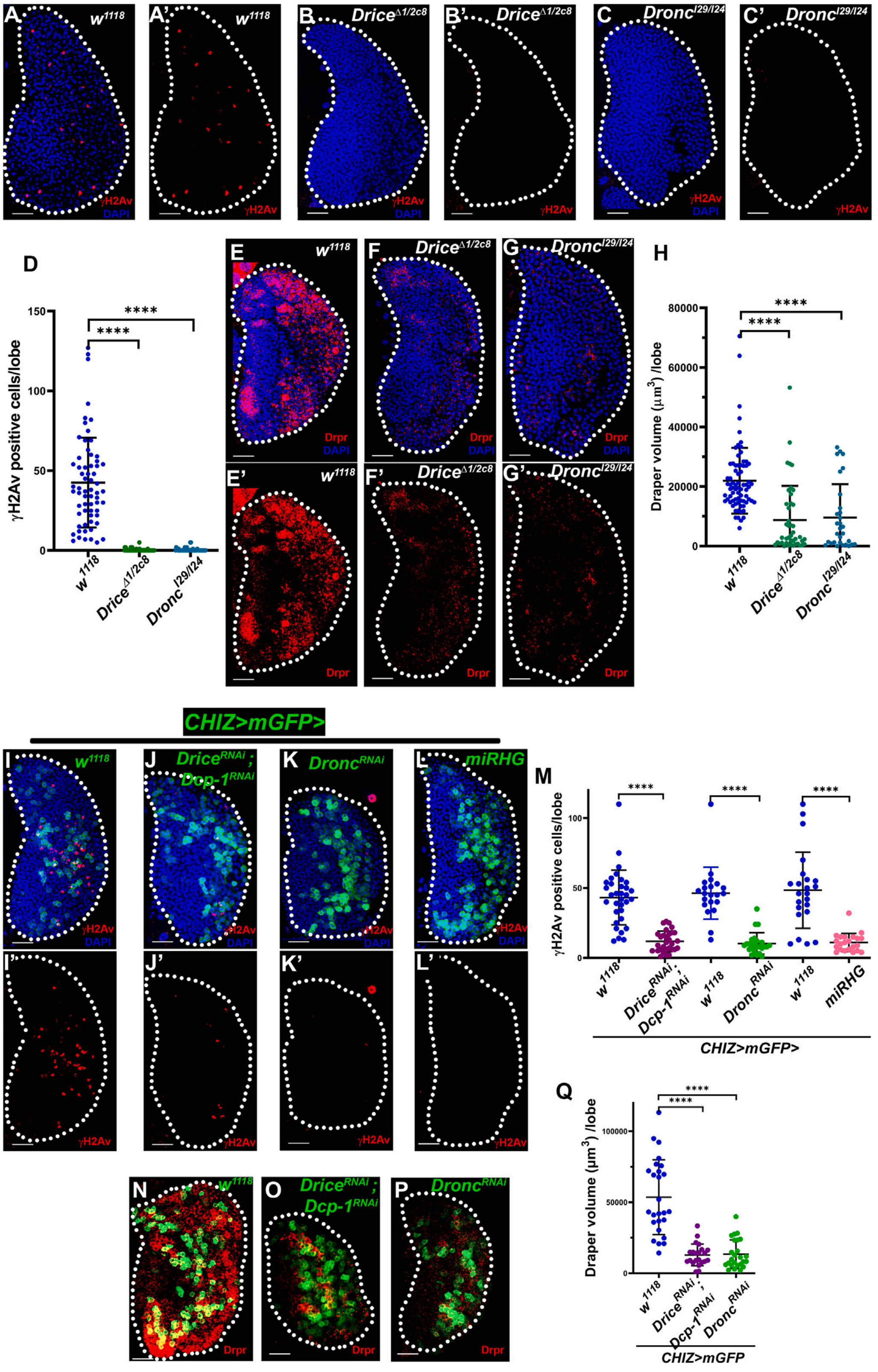

(legend on next page)

along with DDR only in a subset of differentiating cells in a temporal manner.

Finally, we used a caspase lineage trace marker line, L-CasExpress L-Trace,[54] to trace the lymph gland executioner caspase-activated cells. Briefly, a membrane-bound LexA is cleaved upon executioner caspase activation and transported to the nucleus to bind lexAOP regulators. Nuclear RFP marked the caspase active cells in real time, while flippase expression caused somatic recombination in the same cells to permanently mark progeny cells with nuclear GFP (Figure S2Q).[54] Remarkably, the third-instar lymph gland showed RFP-positive cells in the intermediate zone, while the lineage trace GFP-positive cells were distributed throughout the differentiated zone (Figures 2M and 2N). Caspase lineage cells (GFP+) comprised 27% of lymph gland cells (Figures 2N–2O), similar to hemolectin-positive cells in the differentiated zone shown by Spratford et al.[40] The majority of mature macrophages were P1 positive[19,55] (Figures S2R–S2R″), but only 9% of the crystal cells[19] were caspase lineage trace positive (Figures S2S and S2T). Of note, crystal cells involved in melanization and blood clotting may also arise from immature macrophages, depending on active Notch singling.[56] This suggests that the crystal cell differentiation is independent of caspase activation. These results show that lymph-gland-differentiating cells with transiently activated caspases survive and populate the differentiated zone with macrophages.

Caspase-mediated DNA damage is required for macrophage differentiation

The *Drosophila* apoptotic pathway (Figure 2A) was then examined in macrophage differentiation. In executioner caspase mutants (*Drice*^{2c8}/*Drice*$^{\Delta 1}$)[57,58] and initiator caspases (*Dronc*^{I24}/*Dronc*^{I29}),[59] we found severely low numbers of γH2Av-positive cells (Figures 3A–3D) and macrophages marked by the phagocytic receptor Draper[60] (Figures 3E–3H)[61] and P1 (Figures S3A–S3C). In *Drice* and *Dronc* mutants, we observed that the active Dcp-1-positive cells were absent (Figures S3D–S3G). However, another executioner caspase Dcp-1 null mutant (*Dcp-1*^{Prev1})[62] showed Dcp-1- and γH2Av-positive cells similar to the control group (Figures S3H–S3L′). These results suggest that Dronc and Drice caspases regulate lymph gland progenitor differentiation.

To exclude caspase mutant phenotypes caused by systemic signals that maintain lymph gland progenitors,[32,33] we downregulated the apoptotic pathway in the intermediate progenitors by expressing microRNA against reaper, hid, and grim (RHG) transcripts (*UAS-miRHG*).[63] This resulted in significantly fewer γH2Av-positive cells (Figures 3I, 3I′, 3L, 3L′, and 3M) and the loss of caspase active cells (Figures S3O, S3O′, S3R, and S3S). Inhibiting executioner caspase by expression of baculovirus protein P35[63] also caused a similar phenotype (Figures S3M–S3N′). Depletion of both executioner caspases using RNA interference (RNAi) for *Drice* and *Dcp-1*[64] in the intermediate progenitors resulted in significantly fewer DDR cells (Figures 3I–3J′ and 3M), with substantially lower phagocytic marker Draper-positive macrophages (Figures 3N–3O and 3Q). After knocking down Dronc in the intermediate progenitor with *Dronc*^{RNAi}, γH2Av-positive cells (Figures 3I, 3K, and 3M) and Draper staining significantly decreased (Figures 3N, 3P, and 3Q). Dcp-1-positive cell numbers were reduced in both Drice and Dronc depletion backgrounds (Figures S3O–S3Q′ and S3S). These findings demonstrate that DNA breaks and phagocytic macrophage differentiation require the caspase signaling cascade.

CAD induces DNA breaks required for macrophage differentiation

Among the diverse roles of caspases in myeloid-type progenitors, we explored the caspase-activated proteins that cause DNA breaks. The CAD causes DNA breaks after the caspase cleaves its inhibitor ICAD (inhibitor CAD). ICAD binding supports CAD's folding and keeps it inhibited. The freed CAD dimerizes and functions as DNase, which causes DNA fragmentation during apoptosis.[65,66] The *Drosophila* DNA fragmentation factor-related protein 1 (Drep1) is the ICAD homolog, and Drep4 is the CAD homolog.[67–69] We examined if *Drosophila* CAD/ICAD causes lymph gland DNA breaks since we found caspase and DDR activity in the same cells (Figure 2D). We used lymph gland intermediate progenitor driver *CHIZ>mGFP* and progenitor driver *dome*^{MESO}*>2xEGFP* to knock down *Drosophila* ICAD (*Drep1*^{RNAi}) and CAD (*Drep4*^{RNAi}) using multiple RNAi lines. The knockdown of ICAD and CAD in intermediate progenitors caused significantly fewer γH2Av-positive cells in the lymph gland (Figures 4A–4C′, S4A–S4B′, and 4G). Concomitantly, the macrophage differentiation marked by Draper (Figures 4D–4F

Figure 3. Caspase-mediated DNA breaks needed for macrophage differentiation

(A–C′) Control *w*^{1118} (n = 74, A and A′) lymph gland shows γH2Av staining (red), but executioner caspase *Drice*$^{\Delta 1}$/*Drice*^{2c8} (n = 43, B and B′) and initiator caspase *Dronc*^{I29}/*Dronc*^{I24} (n = 30, C and C′) mutants do not show γH2Av-positive cells.

(D) Quantification of γH2Av-positive cells in (A)–(C′).

(E–G′) Phagocytic receptor Draper staining (red) as macrophage marker in control *w*^{1118} (n = 64, E and E′) but with severely less staining in mutants *Drice*$^{\Delta 1}$/*Drice*^{2c8} (n = 71, F and F′) and *Dronc*^{I29}/*Dronc*^{I24} (n = 51, G and G′).

(H) Quantification of Draper staining volume in (E)–(G′).

(I–L′) Compared with the control sets, *CHIZ>mGFP/+* (n = 33, n = 21, and n = 23, I and I′), *CHIZ>mGFP*-driven *UAS-Drice*^{RNAi}*; UAS-Dcp-1*^{RNAi} (n = 30, J and J′), *UAS-Dronc*^{RNAi} (n = 24, K and K′), and *UAS-miRHG* (L and L′) lymph glands show fewer γH2Av-positive cells (red).

(M) Quantification of γH2Av-positive cells in (I)–(L′).

(N–P) Control lymph gland, *CHIZ>mGFP/+* (n = 26, N), *CHIZ>mGFP*-driven *UAS-Drice*^{RNAi}*; Dcp-1*^{RNAi} (n = 22, O), and *UAS-Dronc*^{RNAi} (n = 25, P) show drastically lower Draper staining (red) than the control.

(Q) Quantification of Draper volume in (N) and (P).

Scale bars: 25 μm, maximum-intensity projections of the middle third optical section of the wandering third-instar larval lymph gland lobe. DAPI (blue)-stained nuclei. ****$p < 0.0001$ Error bars, mean ± SD. Control groups are different for their respective experimental sets because experiments were performed on different days. All images represent 3 or more independent biological experiments, and n represents lymph gland lobe numbers.

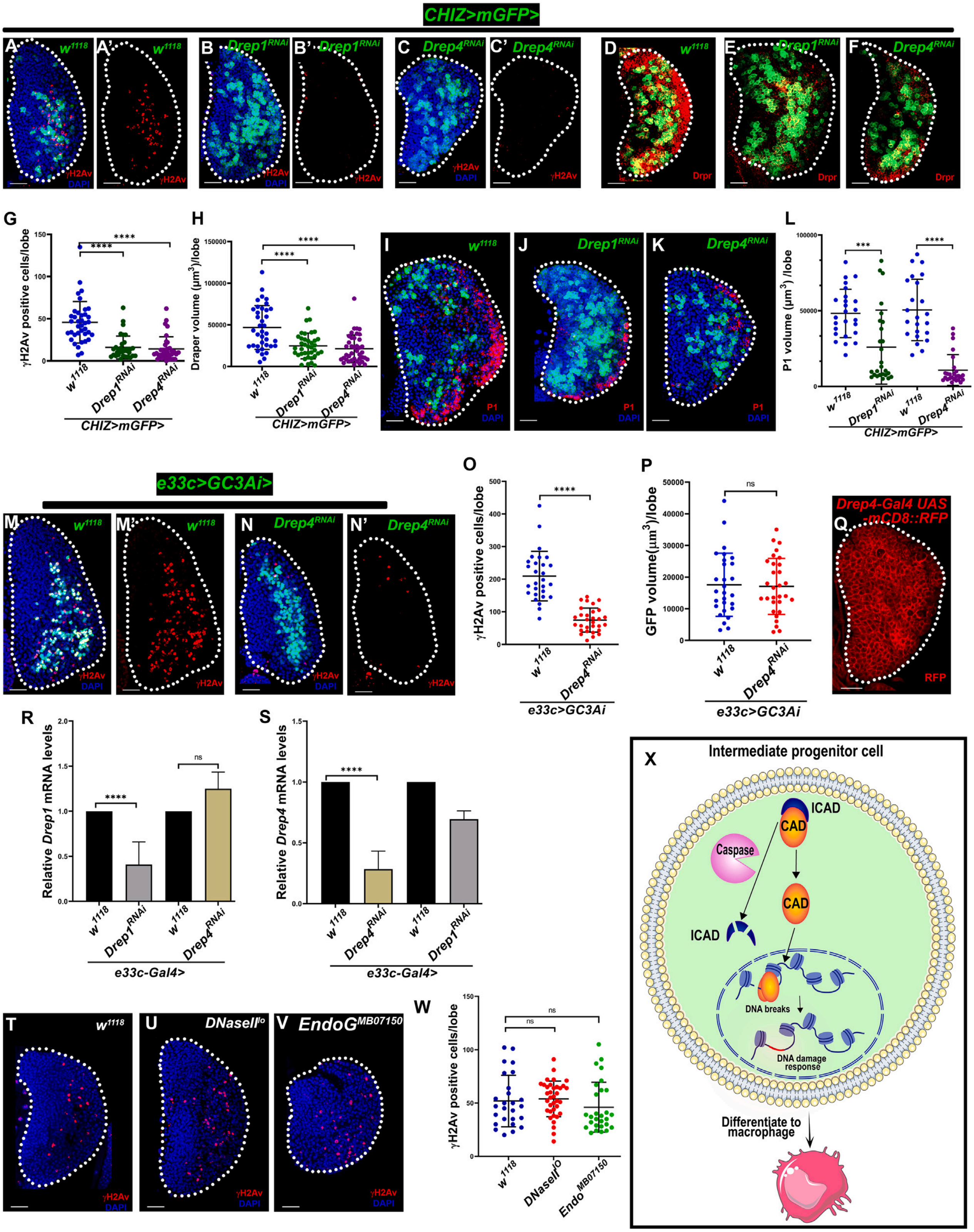

(legend on next page)

and 4H) and P1 (Figures 4I–4L) was also significantly reduced. ICAD and CAD depletion in progenitors (*dome*^{MESO}*>Drep1*^{RNAi} or *Drep4*^{RNAi}) also reduced γH2Av-positive cells (Figures S4C–S4E). Depleting CAD in the whole lymph glands (*e33c>GC3Ai>-Drep4*^{RNAi}) did not affect the caspase activity but significantly reduced γH2Av-positive cells (Figures 4M–4P), indicating that CAD causes DNA breaks in the lymph gland during macrophage development. Since antibodies against *Drosophila* CAD/ICAD are unavailable, we used a *Drep4 T2A-Gal4* line[70] to drive *UAS-mRFP* to recapitulate *Drep4* gene expression patterns. Most lymph gland cells were *Drep4>mRFP*+ (Figure 4Q). We used quantitative RT-PCR to assess the transcript levels of *Drep1*/*Drep4* (*ICAD*/*CAD*) in the lymph gland and the efficiency of the used RNAi lines. Drep1 and Drep4 genes expressed in lymph glands and RNAi lines effectively reduced their transcript levels. The Drep4 transcript did not change substantially in Drep1-depleted lymph glands, though the Drep1 transcript slightly increased in Drep4-depleted lymph glands (Figures 4R and 4S). These results suggest that Drep1 (ICAD) depletion causes a phenotype similar to that of Drep4 (CAD) because Drep4 (CAD) might not properly fold and degrade, resulting in the similar phenotypes observed in Drep1 and Drep4 knockdown backgrounds. This is consistent with previous *in vitro* studies on CAD/ICAD.[65–69]

Besides the CAD, DNaseII and endonuclease G (Endo G) also contribute to DNA breaks via alternative apoptotic signaling.[71,72] To assess the role of DNaseII and Endo G in blood progenitor differentiation, we examined γH2Av immunostaining in the homozygous *DNaseII*^{lo}, a hypomorphic allele, and in an Endo G mutant (*EndoG*^{MB07150}).[72] The γH2Av-positive cell numbers were unaffected in both homozygous mutants (Figures 4T–4W). Thus, DNaseII and Endo G were not involved in DNA damage in the lymph gland. Together, our results show that *Drosophila* caspase signaling-dependent, CAD-mediated DNA breaks are required in developing macrophages (Figure 4X).

InR/PI3K/Akt signaling regulates caspase activity and DDR in macrophage differentiation

Mechanisms that trigger apoptotic signaling and potentially involve DNA damage during macrophage development were investigated to determine the physiological relevance of the above results. Previous studies showed that several signaling pathways in the lymph gland can influence the behavior of blood progenitors.[27,28,32,73] We screened candidate genes involved in several signaling pathways in the lymph gland intermediate progenitors (Figures 5A–5E′ and S5A–S5I). Among these, we found Akt to be crucial for caspase activation, as intermediate progenitors with Akt knockdown (*CHIZ>mGFP>Akt*^{RNAi}) had significantly fewer Dcp-1-positive cells (Figures 5A–5B′ and 5V). We investigated whether InR/PI3K/Akt-mediated signaling is involved. The expression of the PI3K dominant-negative form (*UAS-PI3K92E*^{DN}) and InR depletion in the intermediate progenitors (*CHIZ>mGFP>InR*^{RNAi}) significantly reduced Dcp-1-positive cells (Figures 5A, 5A′, 5C–5D′, and 5V). A recent study showed that PI3K/Akt signaling activation induces autonomous apoptotic stress.[54] In agreement, we found a significant decrease in γH2Av-positive cells (Figures 5H–5K′ and 5W) following the expression of *InR*^{RNAi}, *Akt*^{RNAi}, and *PI3K*^{DN} in intermediate progenitors. Draper staining levels in lymph glands were also significantly reduced in these backgrounds (Figures 5O–5R and 5X), similar to InR/Akt-regulated glia.[60,74] The lymph gland volume in these genetic backgrounds was significantly reduced (Figure S5L). However, the number of CHIZ^{+} cells remained unchanged (Figures S5M), suggesting that blocking InR/PI3K/Akt signaling stops lymph gland differentiation. Interestingly, Akt depleted in the intermediate progenitors did not affect crystal cell number (Figures S5N–S5P), but the P1 volume decreased significantly (Figures S5Q, S5R, and S5T). These results show that InR/PI3K/Akt signaling inhibition reduces macrophage differentiation and caspase-mediated DNA damage.

We then investigated whether PI3K/Akt signaling overactivation increases caspase activity and macrophage differentiation. In the

Figure 4. Caspase-activated DNase induces DNA breaks required for macrophage differentiation

(A–C′) Depletion of *Drosophila* ICAD (*CHIZ>mGFP; UAS-Drep1*^{RNAi}, *n* = 34, B and B′) and CAD (*CHIZ>mGFP*; *UAS-Drep4*^{RNAi}, *n* = 35, C and C′) in the intermediate progenitors (green) leads to significantly reduced γH2Av-positive lymph glands cells (red) compared to control, *CHIZ>mGFP/+* (*n* = 38, A and A′).

(D–F) Depletion of ICAD (*CHIZ>mGFP UAS-Drep1*^{RNAi}, *n* = 38, E) and CAD (*CHIZ>mGFP; UAS-Drep4*^{RNAi}, *n* = 40, F) causes significantly less Draper staining (red) in lymph gland compared to control, *CHIZ>mGFP/+* (*n* = 37, D).

(G) Quantification of γH2Av-positive cells in (A)–(C′).

(H) Quantification of Draper volume in (D)–(F).

(I–K) Depletion of ICAD (*CHIZ>mGFP UAS-Drep1*^{RNAi}, *n* = 26, J) and CAD (*CHIZ>mGFP; UAS-Drep4*^{RNAi}, *n* = 26, K) causes significantly reduce P1 staining (red) in lymph gland compared to their control sets, *CHIZ>mGFP/+* (*n* = 25 and 23, I).

(L) Quantification of P1 volume in (I)–(K).

(M–N′) Loss of CAD in the lymph gland (*UAS-GC3Ai/UAS-Drep4*^{RNAi}*; e33c-Gal4/+*, *n* = 30) shows fewer γH2Av-positive cells (red) but unchanged caspase-activated (*GC3Ai*) cells compared to control (*UAS-GC3Ai/+; e33c-Gal4/+*, *n* = 28, M and M′).

(O) Quantification of γH2Av-positive cells in (M)–(N′).

(P) Quantification of GFP volume of GC3Ai reporter in (M)–(N′).

(Q) *Drosophila* CAD (Drep4) expressed (*Drep4-Gal4, UAS-mCD8::RFP*) in third-instar larval lymph gland.

(R and S) Quantitative RT-PCR shows that lymph gland (*e33c-Gal4*) expressed Drep1/ICAD (R) and Drep4/CAD (S); *UAS-Drep1*^{RNAi} significantly reduced Drep1 transcript but not Drep4 transcript (R); and *UAS-Drep4*^{RNAi} significantly reduced Drep4 transcript but moderately changed Drep1 transcript (S).

(T–V) *DNaseII*^{lo} (U) and *EndoG*^{MB07150} (V) mutants have γH2Av-positive cells (red) similar to control w^{1118} (T) lymph glands.

(W) Quantification of γH2Av-positive cells in (T)–(V).

(X) Model showing active caspase causing CAD-mediated DNA breaks at the open chromatin regions during macrophage differentiation.

Images are from the wandering third-instar larval lymph glands. Scale bar: 25 μm. Images are maximum intensity projections of the middle third optical section of lymph glands except (M) is a single optical section. DAPI (blue)-stained nuclei. ***$p < 0.001$ and ****$p < 0.0001$; ns, not significant. Error bars, mean ± SD. All images represent 3 or more independent biological experiments, and *n* represents lymph gland lobe numbers.

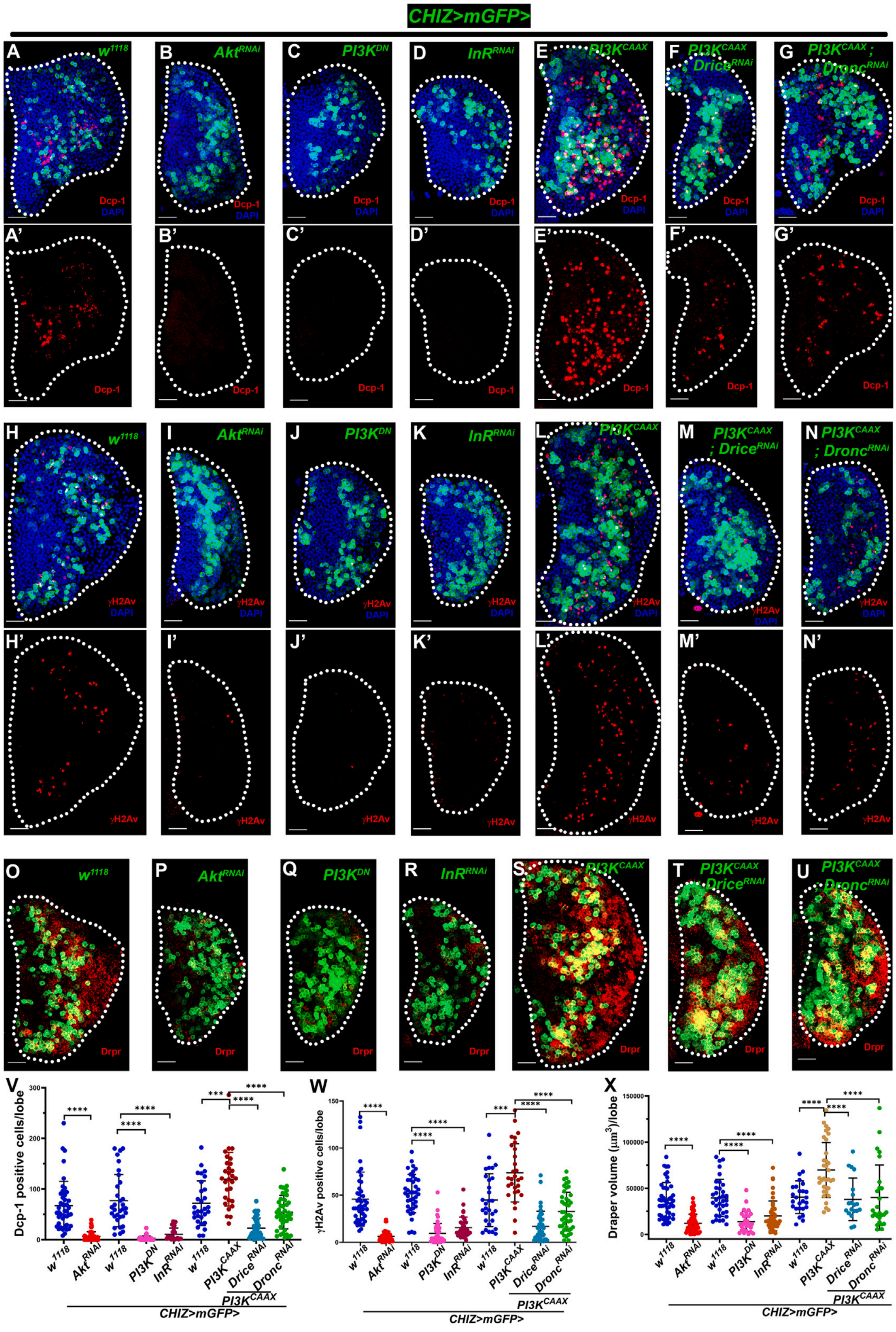

(legend on next page)

early third-instar lymph gland, all CHIZ⁺ cells showed high caspase activity upon expressing a constitutively activated PI3K (*UAS-PI3K*^{CAAX})[75] in intermediate progenitors (Figures S5U–S5V′). The Dcp-1-positive (Figures 5A–5A′, 5E–5E′ and 5V) and γH2Av-positive cell numbers also increased significantly (Figures 5H, 5H′, 5L, 5L′, and 5W) in the wandering third-instar lymph glands. Further, CHIZ⁺ cell numbers and lymph gland size also increased significantly (Figures S5L and S5M). To test if the increase in caspase activity and DDR is a cell-type-specific role of activated PI3K/Akt in intermediate progenitors, we expressed *PI3K*^{CAAX} and *Akt*^{RNAi} in progenitor cells using the *dome*^{MESO}*-Gal4* driver. Remarkably, in the *PI3K*^{CAAX} background, most lymph glands fell apart at the wandering third-instar stage, and the Dcp-1- and γH2Av-positive cells were present in high numbers only in the differentiating zone instead of in the core progenitor zone (Figures S5W and S5X), while in the *Akt*^{RNAi} background, the lymph glands were smaller, with fewer γH2Av-positive cells (Figures S5W and S5Y). We performed Draper and P1 staining to determine if *PI3K*^{CAAX} overexpression increased caspase activity in the intermediate progenitors and affected macrophage differentiation. We observed significantly high numbers of macrophages (Figures 5O, 5S, 5X, S5Q, S5S, and S5T).

The control *CHIZ>mGFP* genotype showed positive immunostaining for phosphorylated Akt (p-Akt) throughout the third-instar lymph gland, with less intense staining in the differentiated zone (Figures S5Z, 5Z′, S5ZC, and S5ZC′). Akt-depleted intermediate progenitors (*CHIZ>mGFP>Akt*^{RNAi}) resulted in decreased p-Akt (Figures S5ZA, S5ZA′, S5ZD, and S5ZD′). However, p-Akt staining in *PI3K*^{CAAX} overexpression using *CHIZ-GAL4* resulted in dramatically high p-Akt in CHIZ⁺ cells (Figures S5ZB, 5ZB′, S5ZE, and S5ZE′).

Furthermore, *CHIZ-GAL4*-driven *Drice*^{RNAi} or *Dronc*^{RNAi} in *PI3K*^{CAAX} overexpression background lymph glands significantly reduced the number of Dcp-1 (Figures 5A, 5A′, 5E–5G′, and 5V), γH2Av-positive cells (Figures 5H, 5H′, 5L–5N′, and 5W), Draper staining (Figures 5O, 5S–5U, and 5X), lymph gland size, and CHIZ⁺ cells (Figures S5L and S5M). These findings indicate that intermediate progenitors have active Akt signaling and that it differentiates macrophages. However, the possibility of other signaling pathways playing a partially redundant role cannot be ruled out.

Caspase-activated DNA breaks regulated by PI3K/Akt-mediated Ask1/JNK signaling

It is reported that InR/PI3K/Akt signaling phosphorylates active p-Thr Ask1 on the N-terminal Ser83 residue attenuates Ask1 activity, resulting in a low level of JNK activity.[76] Also, high ROS-mediated Ask1/JNK signaling activation is associated with apoptosis.[76–78] Interestingly, lymph gland cell differentiation is linked with ROS-mediated JNK activity.[35] The intermediate progenitor zone showed the known JNK reporters like extracellular protein matrix metalloprotease 1 (MMP1)[79] (Figures 6A and 6A′), TRE-dsRed[80] (Figures S6A and S6A′), and puc-lacZ[35] (Figures S6C and S6C′). We also found TRE-dsRed-positive cells to co-localize with caspase active cells (GC3Ai positive) (Figures S6B and S6B′) and the puc-lacZ-positive cells to be also γH2Av positive (Figures S6D–S6D″), suggesting that JNK signaling potentially activates the caspase-dependent DNA breaks. Ask1 and JNK knockdown in intermediate progenitors (*CHIZ>mGFP>Ask1*^{RNAi} or *JNK*^{RNAi}) severely reduced immunostaining for MMP1- (Figures 6A–6C′ and 6F), Dcp-1- (Figures 6G–6J), and γH2Av-positive cells (Figures 6K–6N and S6U–S6V′), along with drastically decreasing macrophage differentiation (Figures 6O–6Q, S6S, and S6T). JNK depleted in the whole lymph gland (*e33c>GC3Ai>JNK*^{RNAi}) significantly reduced active caspase cells (Figures S6M–S6O). This supports previous findings that active JNK signaling contributes to progenitor differentiation.[35,81] Next, we determined whether PI3K/Akt interacts with Ask1/JNK signaling in caspase-mediated DNA damage by depleting Akt and Ask1 in the *PI3K*^{CAAX} overexpression background in intermediate progenitors. Dcp-1- and γH2Av-positive cells were significantly reduced in *PI3K*^{CAAX}*; Akt*^{RNAi} and *PI3K*^{CAAX}*; Ask1*^{RNAi} backgrounds (Figures 6R–6V, S6U, and S6X–S6ZA). However, p-Akt immunostaining remained high as *PI3K*^{CAAX} overexpression in the *PI3K*^{CAAX}*; Ask1*^{RNAi}, though it was severely reduced in *PI3K*^{CAAX}*; Akt*^{RNAi} (Figures S6ZC–S6ZF′). Further, we expressed a serine-to-alanine mutated Ask1 (*UAS-Ask1*^{S83A}),[76] which phenocopied the Ask1 knockdown phenotype of MMP1 staining (Figures S6E–S6F′ and S6I) and significantly reduced the number of Dcp-1- (Figures S6J–S6L) and γH2Av-positive cells (Figures S6U, S6U′, S6W, S6W′, and S6ZA) and Draper staining (Figures S6P–S6R). This supports previous findings that Ser83 p-Ask1 maintains a sublethal level of JNK/caspase activity.[76]

Figure 5. InR/PI3K/Akt signaling regulates caspase activity and DNA breaks in macrophage differentiation

(A–G′) InR/PI3K/Akt-mediated executioner caspase regulation: Dcp-1 staining (red) in three different control sets and *CHIZ>mGFP/+* (*n* = 43, *n* = 32, and *n* = 30, A and A′, green) and *CHIZ>mGFP*-driven experimental sets (*Akt*^{RNAi}, B and B′, *n* = 50; *PI3K*^{DN}, C and C′, *n* = 37; and *InR*^{RNAi}, D and D′, v992, *n* = 22) show fewer Dcp-1-positive cells. *CHIZ>mGFP*-driven *PI3K*^{CAAX} (E and E′, *n* = 29) have high Dcp-1-positive cells and are rescued in *PI3K*^{CAAX}*; Drice*^{RNAi} (F and F′, *n* = 42) and *PI3K*^{CAAX}; *Dronc*^{RNAi} (G and G′, *n* = 43).

(H–N′) *CHIZ>mGFP* (green)-driven experimental sets (*Akt*^{RNAi}, I and I′, *n* = 47; *PI3K*^{DN}, J and J′, *n* = 48; and *InR*^{RNAi}, K and K′, v992, *n* = 36) show fewer γH2Av-positive cells (red) compared to control sets, *CHIZ>mGFP/+* (H and H′, *n* = 43, *n* = 40, and *n* = 30); *CHIZ>mGFP*-driven *PI3K*^{CAAX} (L and L′, *n* = 29) show high γH2Av-positive cells and are rescued in *PI3K*^{CAAX}*; Drice*^{RNAi} (M and M′, *n* = 42) and *PI3K*^{CAAX}; *Dronc*^{RNAi} (N and N′, *n* = 43).

(O–U) *CHIZ>mGFP* (green)-driven experimental sets (*Akt*^{RNAi}, P, *n* = 52; *PI3K*^{DN}, Q, *n* = 30; and *InR*^{RNAi}, R, BL31037, *n* = 45) show less Draper (red) compared to control sets, *CHIZ>mGFP/+* (O, *n* = 46, *n* = 35, and *n* = 26); *CHIZ>mGFP*-driven *PI3K*^{CAAX} (S, *n* = 34) have significantly high Draper and are rescued in *PI3K*^{CAAX}*; Drice*^{RNAi} (T, *n* = 19) and *PI3K*^{CAAX}; *Dronc*^{RNAi} (U, *n* = 26).

(V) Quantification of Dcp-1-positive cells in (A)–(G′).

(W) Quantification of γH2Av-positive cells in (H)–(N′).

(X) Quantification of Draper volume in (O)–(U).

All images show maximum intensity projections of the middle third optical section of wandering third-instar lymph gland lobes. Scale bars: 25 μm. DAPI (blue)-stained nuclei. $^{**}p < 0.01$, $^{***}p < 0.001$, and $^{****}p < 0.0001$. Error bars, mean ± SD. All images represent 3 or more independent biological experiments, and *n* represents lymph gland lobe numbers.

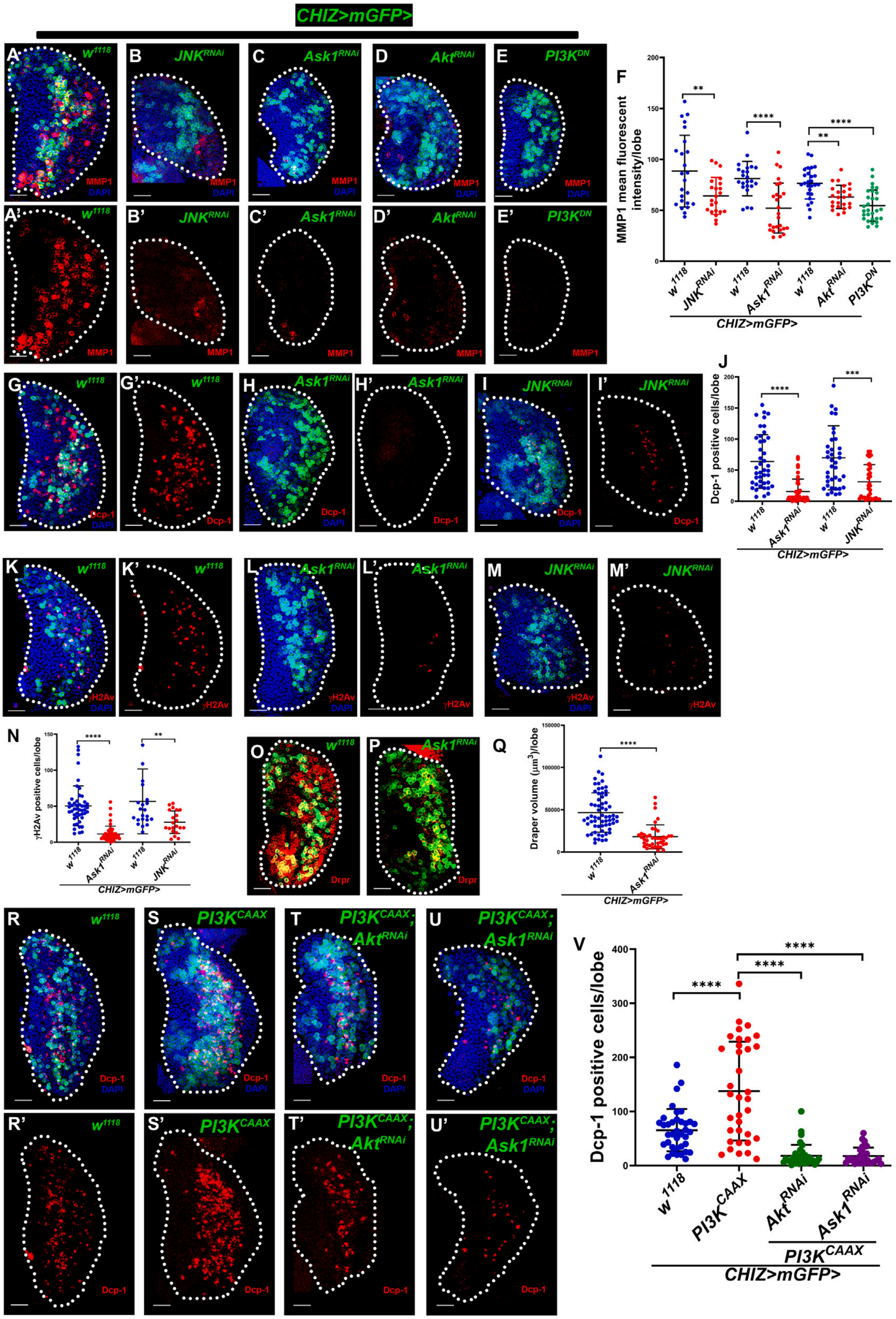

(legend on next page)

CellPress
OPEN ACCESS

Expression of Akt^{RNAi}, $PI3K^{DN}$, and InR^{RNAi} in the intermediate progenitors (*CHIZ-Gal4*) showed a dramatically reduced MMP1 (Figures 6A, 6A′, 6D–6F, S6E, S6E′, S6G, S6G′, and S6I), whereas overexpression of $PI3K^{CAAX}$ significantly increased MMP1 (Figures S6E, S6E′, S6H, and S6I). These data suggest that PI3K/Akt signaling regulates Ask1/JNK activity for caspase-mediated DNA damage in the lymph gland. Since ROS also regulates JNK activity in the lymph gland,[35] we tested glutathione S-transferase D (gstD) activity using *gstD-GFP* as a ROS reporter[82] and found that γH2Av-positive cells have low gstD-GFP (Figures S6ZB–S6ZB″) compared to progenitors. Overall, our results indicate that PI3K/Akt signaling regulates the ROS/Ask1/JNK axis to maintain sublethal caspase activity for macrophage differentiation.

Macrophage differentiation requires PI3K/Akt signaling to regulate caspase-CAD-mediated DNA breaks

To confirm genetic interaction between PI3K/Akt signaling and CAD (Drep4), we used the *CHIZ-GAL4* driver to knock down CAD (Drep4) in a $PI3K^{CAAX}$ overexpression background to determine if it rescues the high DNA damage and macrophage differentiation. This intervention severely reduced the number of γH2Av-positive cells (Figures 7A–7C′ and 7D) and macrophage differentiation (Figures 7F–7I) compared to $PI3K^{CAAX}$ overexpression backgrounds. However, Dcp-1-positive cells remained high (Figures 7A–7C″ and 7E), and lymph gland size (DAPI-stained cell volume) and $CHIZ^{+}$ cells were (Figures S7A and S7B) similar to the $PI3K^{CAAX}$ overexpression background. These findings show that the differentiation of macrophages relies on CAD/ICAD-mediated DNA damage induced by InR/PI3K/Akt signaling in the lymph gland. Based on these genetic interaction findings, we propose a model for myeloid-type progenitor differentition into macrophages via developmental signaling-induced caspase-activated DNA breaks (Figure 7J).

Embryonic-origin macrophages require caspase activation for efficient phagocytosis

Like vertebrates, early embryonic *Drosophila* hematopoiesis produces macrophages dispersed throughout the embryo, which later populate the larval sessile and circulating blood cells.[83] We examined embryonic-origin circulating blood cells from third-instar larvae to see whether they are experiencing caspase activity during their development by using a caspase lineage reporter (*L-CasExpress L-Trace*) that can mark caspase-activated lineage cells with GFP.[54] Remarkably, 60% of circulating cells are caspase lineage positive (Figures 7K and 7L). We live imaged embryos using *L-CasExpress L-Trace* and *CasExpress-Gal4; G-Trace*^{LTO} caspase linage reporters in the *srp-mCherry*[84] background, where *srp-mCherry* marked the embryonic macrophages to find if embryonic macrophages also experienced executioner caspase activation. Remarkably, the embryonic macrophages were positive for caspase reporters like L-*CasExpress*-GFP (Figures 7M and 7M′) and *CasExpress>GFP* (Figures S7C–S7D′; Videos S1 and S2). However, *L-caspase L-Trace* lineage-positive cells, which are abundant in the dorsal closure region (Figures S7E and S7E′; Video S3) at developmental stage 13, when many cells die, served as a control tissue for our experiments. These macrophages express Draper, a single-pass transmembrane receptor involved in phagocytosis.[85] Thus, we monitored Draper expression using a *Draper-GFP* line,[86] which is highly expressed in embryonic-origin larval circulating blood cells (Figure S7F), and Draper antibody staining co-localizes with Draper-GFP in the lymph gland differentiated zone (Figures S7G–S7G″). Finally, we performed a phagocytic assay[87] using fluorescently labeled *E. coli* (RFP) in wandering third-instar circulating macrophages (Hml^{Δ}*-Gal4, UAS-2xEGFP*) and found a significant decrease in their number and phagocytic efficiency of bacteria in caspase mutant ($Drice^{2c8/\Delta1}$) macrophages (Figures 7N–7Q; Video S4). Collectively, our findings indicate that *Drosophila* phagocytic macrophage differentiation also requires sublethal caspase activity.

DISCUSSION

Multifunctional phagocytic macrophages populate most tissue during fetal development and can self-renew.[1,2,4] However, the macrophage differentiation mechanisms remain unknown. Here, we show that during the normal development of *Drosophila*

Figure 6. InR/PI3K/Akt signaling via the Ask1/JNK axis regulates caspase activity

(A–E′) JNK signaling activity using MMP1 staining (red) in *CHIZ>mGFP* (green)-driven JNK^{RNAi} (B and B′), $Ask1^{RNAi}$ (C and C′), Akt^{RNAi} (D and D′), and $PI3K^{DN}$ (E and E′) is severely reduced compared to their respective control sets, *CHIZ>mGFP/+* ($n = 24$, $n = 23$, and $n = 26$).
(F) Quantification of mean fluorescent intensity of MMP1 in (A)–(E).
(G–I′) *CHIZ>mGFP*-driven $Ask1^{RNAi}$ ($n = 38$, H and H′) and JNK^{RNAi} ($n = 35$, I and I′) show that positive cells (red) significantly decrease compared to control, *CHIZ>mGFP/+* ($n = 42$ and $n = 35$, G and G′).
(J) Quantification of Dcp-1-positive cells in (G)–(I′).
(K–M′) *CHIZ>mGFP*-driven $Ask1^{RNAi}$ ($n = 50$, L and L′) and JNK^{RNAi} ($n = 21$, M and M′) show that γH2Av-positive cells (red) drastically decrease compared to respective control sets, *CHIZ>mGFP/+* ($n = 47$ and $n = 22$, K and K′).
(N) Quantification of γH2Av-positive cells in (K)–(M′).
(O and P) Draper staining (red) in *CHIZ>mGFP*-driven $Ask1^{RNAi}$ ($n = 41$, P) is significantly decreased compared to control, *CHIZ>mGFP/+* ($n = 64$, O).
(Q) Quantification of Draper volume in (O)–(P).
(R–U′) Depletion of Akt (*CHIZ>mGFP; UAS-*$PI3K^{CAAX}$*; UAS-* Akt^{RNAi}, $n = 35$, T and T′) and Ask1 (*CHIZ>mGFP; UAS-*$PI3K^{CAAX}$*; UAS-* $Ask1^{RNAi}$, $n = 28$, U and U′) in intermediate progenitors (control *CHIZ>mGFP/+*, $n = 38$, R and R′) rescue the high Dcp-1-positive cells (red) phenotype of $PI3K^{CAAX}$ overexpression (*CHIZ>mGFP*; *UAS-*$PI3K^{CAAX}$, $n = 35$, S and S′).
(V) Quantification of Dcp-1-positive cells in (R)–(U′).
All images are from wandering third-instar lymph gland lobe with maximum-intensity projections of middle third optical sections; Scale bars: 25 μm. DAPI (blue)-stained nuclei. $^{**}p < 0.01$, $^{***}p < 0.001$, and $^{****}p < 0.0001$. Error bars, mean ± SD. All images represent 3 or more independent biological experiments, and n represents lymph gland lobe numbers.
See also Figure S6.

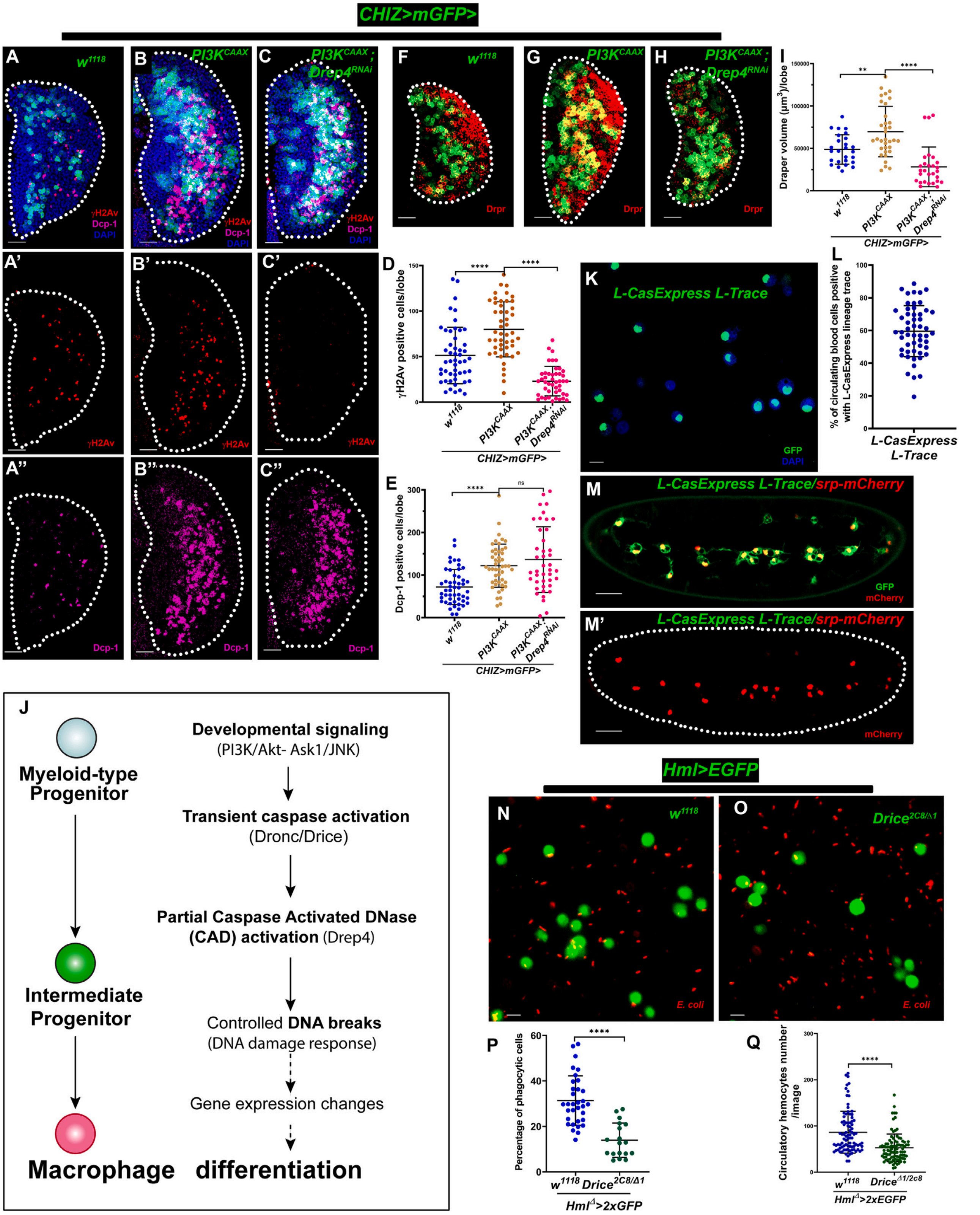

(legend on next page)

CellPress
OPEN ACCESS

macrophages in the larval lymph gland, apoptotic caspases are activated in the differentiating cells. Sublethal executioner caspase activation induces CAD, triggering DNA strand breaks in differentiating macrophages. We find that InR/PI3K/Akt-mediated signaling induces a transient caspase cascade through Ask1/JNK signaling in differentiating macrophages. Furthermore, for efficient phagocytic activity, caspase activation is required in embryonic-origin macrophage development. Therefore, our research using *in vivo* genetic analysis revealed that developmental signal-mediated caspase activation and DDR signals play a role in determining macrophage differentiation during normal development.

In several types of cell differentiation, programmed DNA breaks are reported to coordinate gene expression changes without causing cell death.[88] However, the signals that cause DNA damage in these cases were not addressed. Single-cell transcriptomics on lymph glands revealed a group of cells (1.2%) called cluster X, or GST-rich, with unique genetics and enrichment of DDR, Myb, and cell cycle genes.[81,89] These cells are most likely the CAD-mediated DNA-damaged cells that we report here, as their location and numbers in the lymph gland are comparable. This study revealed that caspase-mediated *Drosophila* CAD causes DNA breaks, which is essential for macrophage differentiation, as depletion of CAD/ICAD in the lymph gland causes loss of phagocytic markers and DNA damage, but caspase activity is still seen.

Many *Drosophila* cells show caspase activation to have nonlethal roles in development and differentiation, as shown by several labs.[16,48,58,90–94] Studies showed that lymph gland progenitors must balance ROS-mediated JNK signaling to maintain and differentiate.[35] ROS in lymph gland progenitors might induce caspase activation in differentiating macrophages, and by the time DDR is seen, ROS becomes lower. Monocyte-to-macrophage differentiation requires CSF1-Akt-mediated caspase activation.[95] We find that InR-mediated PI3K-Akt signaling has a role in autonomous apoptotic activation and caspase activity control, as has been reported.[54] However, a partially redundant role of other signaling (e.g., Pvr,[28] EGFR, GABA-calcium[32,34]) cannot be ruled out at present. Further, the differentiated macrophages require both initiator and effector caspases for Draper expression and phagocytic efficiency. Our data support previous research showing that loss of RHG genes causes low levels of Draper expression in embryonic macrophages.[85] Our lineage trace experiments for caspase-positive cells confirm that differentiated macrophages undergo caspase activation.

How the executioner caspase levels and dynamics predict cell survival vs. cell death remains unclear. A cancer cell line model showed that high caspase activity kills all cells but low levels allow survival.[96] Here, in the differentiating cells, PI3K/Akt signaling through the Ask1/JNK axis regulates caspase and CAD activity at a sublethal level. Also, CAD depletion rescues the PI3K active phenotypes except for caspase activity, suggesting that macrophage differentiation requires InR/PI3K-mediated CAD activation (Figure 7J). Other mechanisms might also help survival after caspase activation.[97,98] For example, caspase-mediated skeletal muscle cell differentiation studies reported that nuclear pore complex trimming alters the intracellular environment,[99] and CAD-mediated DNA damage is repaired by base excision repair protein XRCC1, resulting in gene expression changes.[42,100] Differential accessibility of transient CAD for DNA fragmentations helps cells survive due to their chromatin architecture.[101,102]

Caspase/CAD-mediated DNA breaks for macrophage differentiation may modulate chromatin organization to control macrophage-specific gene expression. CAD-mediated DNA breaks around chromatin modifying CCCTC-binding factor sites (chromatin insulators) induce chromatin landscape change by directly acting on promoter or altering promoter-enhancer interaction, which regulates gene expression.[103–105] A *Drosophila* study showed that DNA damage increases chromatin insulator enrichment at insulator sites by regulating the γH2Av.[106] Interestingly, previous research found that mammalian macrophage functions

Figure 7. Developmental PI3K/Akt signaling regulates caspase/CAD activation for phagocytic macrophage differentiation

(A–C″) CAD depletion (*CHIZ>mGFP; UAS-PI3K^{CAAX}; UAS- Drep4^{RNAi}*, *n* = 45 for γH2Av and *n* = 42 for Dcp-1 staining) in intermediate progenitors (control, *CHIZ>mGFP/+*, *n* = 50, A–A″) rescue high γH2Av-positive cell (red) numbers (B′ and C′), but Dcp-1-positive cell (magenta) numbers (B″ and C″) remain the same as *PI3K^{CAAX}* expression background (*CHIZ>mGFP*; *UAS-PI3K^{CAAX}*, *n* = 48, B–B″).

(D) Quantification of γH2Av-positive cells in (A′)–(C′).

(E) Quantification of Dcp-1-positive cells in (A″)–(C″).

(F–H) CAD depletion (*CHIZ>mGFP; UAS-PI3K^{CAAX}; UAS- Drep4^{RNAi}*, *n* = 28; H) in intermediate progenitors (control, *CHIZ>mGFP/+*, *n* = 26, F) rescue high Draper staining (red) in *PI3K^{CAAX}* expression background (*CHIZ>mGFP*; *UAS-PI3K^{CAAX}*, *n* = 34, G).

(I) Quantification of Draper staining volume in (F)–(H).

(J) Schematic shows that a mechanism of myeloid-type progenitor-to-macrophage differentiation through intermediate progenitor requires transient caspase activation and CAD-mediated DNA breaks.

(K) Third-instar larvae *L-CasExpress L-Trace* (*lex-Aop-Flp::Ubi-FRT-STOP-FRT-GFP/+; L-caspase/+*, *n* = 53) circulating blood cells showing caspase lineage activity (GFP).

(L) Quantification of (K) shows 60% of circulating cells are caspase lineage positive.

(M and M′) Embryonic macrophages (stage 13) marked by *srp-mCherry* (red) are caspase lineage-positive GFP (green) (*L-CasExpress L-Trace*).

(N and O) Third-instar larval circulating macrophages phagocytose RFP-tagged *E. coli* in control *Hml$^{\Delta}$-Gal4, UAS-2xEGFP/+* (L), but mutant *Hml$^{\Delta}$-Gal4, UAS-2xEGFP/+; Drice$^{2c8/\Delta1}$*(*n* = 18, M) show less phagocytic efficiency. See also Video S4.

(P) Quantification of phagocytic circulating macrophages in (N) and (O).

(Q) Quantification of circulating macrophage numbers in (N) and (O).

All the lymph gland images shown from the wandering third-instar lymph glands except (M) and (M′), which are from stage 13 embryo. All scale bars represent 25 μm except (K, N, and O) 10 μm. All images are maximum intensity projections of the middle third optical section except (K) and (M)–(O), which are single optical sections. DAPI (blue)-stained nuclei. $^{**}p < 0.01$ and $^{****}p < 0.0001$; ns, not significant. Error bars, mean ± SD. All images represent 3 or more independent biological experiments, and *n* represents lymph gland lobe numbers.

require a set of transcriptional regulators accomplished by the tissue-specific macrophage chromatin landscape.[5,6] Together, we hypothesize that caspase/CAD-mediated DNA breaks in differentiating macrophages may influence the specification of macrophage fate, possibly by regulating the chromatin landscape and the gene expression that prepares the macrophages for trained immunity[20,85,107] and efficient tissue-specific functions.[1,2,4] Further research will determine how caspase/CAD-mediated DNA breaks cause macrophage-specific gene expression in *Drosophila* and whether these are also relevant to macrophages in higher organisms.

Limitations of the study

Our genetic analysis showed that InR/PI3K/Akt signaling through the Ask1/JNK axis activates sublethal caspase and CAD, causing DNA strand breaks during macrophage differentiation. However, present studies do not rule out other redundant signalings. Due to technical and biological difficulties, we could not determine how Ask1 controls transient caspase activity and the exact levels of caspase activity that cause DNA damage without cell death. We do not know the CAD-mediated DNA damage locations in the developing macrophage genome and DNA repair mechanisms. This DNA breakage could be site specific, which needs to be identified, and may involve the altered chromatin landscape and macrophage-specific gene expression.

STAR★METHODS

Detailed methods are provided in the online version of this paper and include the following:

- KEY RESOURCES TABLE
- RESOURCE AVAILABILITY
  - Lead contact
  - Materials availability
  - Data and code availability
- EXPERIMENTAL MODEL AND STUDY PARTICIPANT DETAILS
- METHOD DETAILS
  - Drosophila lymph gland dissection and immunostaining
  - Dihydroethidium (DHE) staining for ROS
  - Nick translation
  - TUNEL staining
  - *Drosophila* larval staging
  - Circulating blood cells counting
  - Circulating hemocytes immunostaining
  - Phagocytic assay of circulating hemocytes
  - Live imaging of circulating macrophages
  - Embryo live imaging
  - Microscopy and image processing
  - Quantification of lymph gland phenotypes
  - RNA isolation, quantitative reverse transcription PCR analysis
- QUANTIFICATION AND STATISTICAL ANALYSIS

SUPPLEMENTAL INFORMATION

Supplemental information can be found online at https://doi.org/10.1016/j.celrep.2024.114251.

ACKNOWLEDGMENTS

We thank U. Banerjee, A. Bergmann, M. Miura, B. Hay, M. Suzanne, E. Wieschaus, M. Inamdar, D. Bohmann, I.K. Hariharan, F. Serras, I. Ando, and T. Mukherjee for reagents; J. Barman and J. Biswas for preliminary data; and S.C. Lakhotia and Cytogenetics laboratory members for valuable input and support. We acknowledge BDSC and VDRC for fly stocks and DSHB for antibodies. This study was funded by the DBT/Wellcome Trust India Alliance Intermediate Fellowship (IA/I/20/1/504931), the DBT-Ramalingaswami Fellowship (BT/RLF/Re-entry/08/2016), and the Institute of Eminence Scheme, BHU to B.C.M. and CSIR fellowships to D. Maurya and D. Mandal.

AUTHOR CONTRIBUTIONS

Conceptualization, B.C.M. and D. Maurya; methodology, B.C.M. and D. Maurya; investigation and analysis, D. Maurya; visualization, D. Maurya, G.R., and D. Mandal; model preparation, G.R., B.C.M., and D. Maurya; writing, B.C.M. and D. Maurya; funding acquisition and supervision, B.C.M.

DECLARATION OF INTERESTS

The authors declare no competing interests.

Received: December 5, 2023
Revised: April 4, 2024
Accepted: May 3, 2024
Published: May 17, 2024

REFERENCES

1. Lazarov, T., Juarez-Carreño, S., Cox, N., and Geissmann, F. (2023). Physiology and diseases of tissue-resident macrophages. Nature *618*, 698–707. https://doi.org/10.1038/s41586-023-06002-x.
2. Park, M.D., Silvin, A., Ginhoux, F., and Merad, M. (2022). Macrophages in health and disease. Cell *185*, 4259–4279. https://doi.org/10.1016/j.cell.2022.10.007.
3. Mantovani, A., Allavena, P., Marchesi, F., and Garlanda, C. (2022). Macrophages as tools and targets in cancer therapy. Nat. Rev. Drug Discov. *21*, 799–820. https://doi.org/10.1038/s41573-022-00520-5.
4. Cox, N., Pokrovskii, M., Vicario, R., and Geissmann, F. (2021). Origins, Biology, and Diseases of Tissue Macrophages. Annu. Rev. Immunol. *39*, 313–344. https://doi.org/10.1146/annurev-immunol-093019-111748.
5. Gosselin, D., Link, V.M., Romanoski, C.E., Fonseca, G.J., Eichenfield, D.Z., Spann, N.J., Stender, J.D., Chun, H.B., Garner, H., Geissmann, F., and Glass, C.K. (2014). Environment drives selection and function of enhancers controlling tissue-specific macrophage identities. Cell *159*, 1327–1340. https://doi.org/10.1016/j.cell.2014.11.023.
6. Lavin, Y., Winter, D., Blecher-Gonen, R., David, E., Keren-Shaul, H., Merad, M., Jung, S., and Amit, I. (2014). Tissue-resident macrophage enhancer landscapes are shaped by the local microenvironment. Cell *159*, 1312–1326. https://doi.org/10.1016/j.cell.2014.11.018.
7. Wang, Z., Wu, Z., Wang, H., Feng, R., Wang, G., Li, M., Wang, S.Y., Chen, X., Su, Y., Wang, J., et al. (2023). An immune cell atlas reveals the dynamics of human macrophage specification during prenatal development. Cell *186*, 4454–4471.e19. https://doi.org/10.1016/j.cell.2023.08.019.
8. Coillard, A., and Segura, E. (2019). In vivo Differentiation of Human Monocytes. Front. Immunol. *10*, 1907. https://doi.org/10.3389/fimmu.2019.01907.
9. Alvarez-Errico, D., Vento-Tormo, R., Sieweke, M., and Ballestar, E. (2015). Epigenetic control of myeloid cell differentiation, identity and function. Nat. Rev. Immunol. *15*, 7–17. https://doi.org/10.1038/nri3777.
10. Tiwari, S.K., Toshniwal, A.G., Mandal, S., and Mandal, L. (2020). Fatty acid beta-oxidation is required for the differentiation of larval hematopoietic progenitors in Drosophila. Elife *9*, e53247. https://doi.org/10.7554/eLife.53247.
11. Wculek, S.K., Dunphy, G., Heras-Murillo, I., Mastrangelo, A., and Sancho, D. (2022). Metabolism of tissue macrophages in homeostasis and pathology. Cell. Mol. Immunol. *19*, 384–408. https://doi.org/10.1038/s41423-021-00791-9.

CellPress
OPEN ACCESS

12. Clarke, A.J., and Simon, A.K. (2019). Autophagy in the renewal, differentiation and homeostasis of immune cells. Nat. Rev. Immunol. *19*, 170–183. https://doi.org/10.1038/s41577-018-0095-2.

13. Solier, S., Fontenay, M., Vainchenker, W., Droin, N., and Solary, E. (2017). Non-apoptotic functions of caspases in myeloid cell differentiation. Cell Death Differ. *24*, 1337–1347. https://doi.org/10.1038/cdd.2017.19.

14. Hoeffel, G., Chen, J., Lavin, Y., Low, D., Almeida, F.F., See, P., Beaudin, A.E., Lum, J., Low, I., Forsberg, E.C., et al. (2015). C-Myb(+) erythro-myeloid progenitor-derived fetal monocytes give rise to adult tissue-resident macrophages. Immunity *42*, 665–678. https://doi.org/10.1016/j.immuni.2015.03.011.

15. Green, D.R. (2019). The Coming Decade of Cell Death Research: Five Riddles. Cell *177*, 1094–1107. https://doi.org/10.1016/j.cell.2019.04.024.

16. Burgon, P.G., and Megeney, L.A. (2018). Caspase signaling, a conserved inductive cue for metazoan cell differentiation. Semin. Cell Dev. Biol. *82*, 96–104. https://doi.org/10.1016/j.semcdb.2017.11.009.

17. Fuchs, Y., and Steller, H. (2011). Programmed cell death in animal development and disease. Cell *147*, 742–758. https://doi.org/10.1016/j.cell.2011.10.033.

18. McArthur, K., and Kile, B.T. (2018). Apoptotic Caspases: Multiple or Mistaken Identities? Trends Cell Biol. *28*, 475–493. https://doi.org/10.1016/j.tcb.2018.02.003.

19. Banerjee, U., Girard, J.R., Goins, L.M., and Spratford, C.M. (2019). Drosophila as a Genetic Model for Hematopoiesis. Genetics *211*, 367–417. https://doi.org/10.1534/genetics.118.300223.

20. Wood, W., and Martin, P. (2017). Macrophage Functions in Tissue Patterning and Disease: New Insights from the Fly. Dev. Cell *40*, 221–233. https://doi.org/10.1016/j.devcel.2017.01.001.

21. Kharrat, B., Csordas, G., and Honti, V. (2022). Peeling Back the Layers of Lymph Gland Structure and Regulation. Int. J. Mol. Sci. *23*. https://doi.org/10.3390/ijms23147767.

22. Mase, A., Augsburger, J., and Brückner, K. (2021). Macrophages and Their Organ Locations Shape Each Other in Development and Homeostasis - A Drosophila Perspective. Front. Cell Dev. Biol. *9*, 630272. https://doi.org/10.3389/fcell.2021.630272.

23. Melcarne, C., Lemaitre, B., and Kurant, E. (2019). Phagocytosis in Drosophila: From molecules and cellular machinery to physiology. Insect Biochem. Mol. Biol. *109*, 1–12. https://doi.org/10.1016/j.ibmb.2019.04.002.

24. Coates, J.A., Brooks, E., Brittle, A.L., Armitage, E.L., Zeidler, M.P., and Evans, I.R. (2021). Identification of functionally distinct macrophage subpopulations in Drosophila. Elife *10*, e58686. https://doi.org/10.7554/eLife.58686.

25. Li, H., Janssens, J., De Waegeneer, M., Kolluru, S.S., Davie, K., Gardeux, V., Saelens, W., David, F.P.A., Brbić, M., Spanier, K., et al. (2022). Fly Cell Atlas: A single-nucleus transcriptomic atlas of the adult fruit fly. Science *375*, eabk2432. https://doi.org/10.1126/science.abk2432.

26. Rodrigues, D., Renaud, Y., VijayRaghavan, K., Waltzer, L., and Inamdar, M.S. (2021). Differential activation of JAK-STAT signaling reveals functional compartmentalization in Drosophila blood progenitors. Elife *10*, e61409. https://doi.org/10.7554/eLife.61409.

27. Mondal, B.C., Shim, J., Evans, C.J., and Banerjee, U. (2014). Pvr expression regulators in equilibrium signal control and maintenance of Drosophila blood progenitors. Elife *3*, e03626. https://doi.org/10.7554/eLife.03626.

28. Mondal, B.C., Mukherjee, T., Mandal, L., Evans, C.J., Sinenko, S.A., Martinez-Agosto, J.A., and Banerjee, U. (2011). Interaction between differentiating cell- and niche-derived signals in hematopoietic progenitor maintenance. Cell *147*, 1589–1600. https://doi.org/10.1016/j.cell.2011.11.041.

29. Morin-Poulard, I., Vincent, A., and Crozatier, M. (2013). The Drosophila JAK-STAT pathway in blood cell formation and immunity. JAK-STAT *2*, e25700. https://doi.org/10.4161/jkst.25700.

30. Goins, L.M.a.G., Juliet, R., Mondal, B.C., Buran, S., Su, C.C., Tang, R., Biswas, T., and Banerjee, U. (2023). Wnt Signaling Couples G2 Phase Control with Differentiation During Hematopoiesis. Cell Press.

31. Cho, B., Shin, M., Chang, E., Son, S., Shin, I., and Shim, J. (2024). S-nitrosylation-triggered unfolded protein response maintains hematopoietic progenitors in Drosophila. Dev. Cell *59*, 1075–1090.e6. https://doi.org/10.1016/j.devcel.2024.02.013.

32. Shim, J., Mukherjee, T., Mondal, B.C., Liu, T., Young, G.C., Wijewarnasuriya, D.P., and Banerjee, U. (2013). Olfactory control of blood progenitor maintenance. Cell *155*, 1141–1153. https://doi.org/10.1016/j.cell.2013.10.032.

33. Shim, J., Mukherjee, T., and Banerjee, U. (2012). Direct sensing of systemic and nutritional signals by haematopoietic progenitors in Drosophila. Nat. Cell Biol. *14*, 394–400. https://doi.org/10.1038/ncb2453.

34. Goyal, M., Tomar, A., Madhwal, S., and Mukherjee, T. (2022). Blood progenitor redox homeostasis through olfaction-derived systemic GABA in hematopoietic growth control in Drosophila. Development *149*, dev199550. https://doi.org/10.1242/dev.199550.

35. Owusu-Ansah, E., and Banerjee, U. (2009). Reactive oxygen species prime Drosophila haematopoietic progenitors for differentiation. Nature *461*, 537–541. https://doi.org/10.1038/nature08313.

36. Kornepati, A.V.R., Rogers, C.M., Sung, P., and Curiel, T.J. (2023). The complementarity of DDR, nucleic acids and anti-tumour immunity. Nature *619*, 475–486. https://doi.org/10.1038/s41586-023-06069-6.

37. Lake, C.M., Holsclaw, J.K., Bellendir, S.P., Sekelsky, J., and Hawley, R.S. (2013). The development of a monoclonal antibody recognizing the Drosophila melanogaster phosphorylated histone H2A variant (gamma-H2AV). G3 (Bethesda) *3*, 1539–1543. https://doi.org/10.1534/g3.113.006833.

38. Grigorian, M., DeBruhl, H., and Lipsick, J.S. (2017). The role of variant histone H2AV in Drosophila melanogaster larval hematopoiesis. Development *144*, 1441–1449. https://doi.org/10.1242/dev.142729.

39. Evans, C.J., Liu, T., and Banerjee, U. (2014). Drosophila hematopoiesis: Markers and methods for molecular genetic analysis. Methods *68*, 242–251. https://doi.org/10.1016/j.ymeth.2014.02.038.

40. Spratford, C.M., Goins, L.M., Chi, F., Girard, J.R., Macias, S.N., Ho, V.W., and Banerjee, U. (2021). Intermediate progenitor cells provide a transition between hematopoietic progenitors and their differentiated descendants. Development *148*, dev200216. https://doi.org/10.1242/dev.200216.

41. Na, H.J., Akan, I., Abramowitz, L.K., and Hanover, J.A. (2020). Nutrient-Driven O-GlcNAcylation Controls DNA Damage Repair Signaling and Stem/Progenitor Cell Homeostasis. Cell Rep. *31*, 107632. https://doi.org/10.1016/j.celrep.2020.107632.

42. Larsen, B.D., Rampalli, S., Burns, L.E., Brunette, S., Dilworth, F.J., and Megeney, L.A. (2010). Caspase 3/caspase-activated DNase promote cell differentiation by inducing DNA strand breaks. Proc. Natl. Acad. Sci. USA *107*, 4230–4235. https://doi.org/10.1073/pnas.0913089107.

43. Gunesdogan, U., Jackle, H., and Herzig, A. (2014). Histone supply regulates S phase timing and cell cycle progression. Elife *3*, e02443. https://doi.org/10.7554/eLife.02443.

44. Xu, Y.J., and Leffak, M. (2010). ATRIP from TopBP1 to ATR–in vitro activation of a DNA damage checkpoint. Proc. Natl. Acad. Sci. USA *107*, 13561–13562. https://doi.org/10.1073/pnas.1008909107.

45. Blythe, S.A., and Wieschaus, E.F. (2015). Zygotic genome activation triggers the DNA replication checkpoint at the midblastula transition. Cell *160*, 1169–1181. https://doi.org/10.1016/j.cell.2015.01.050.

46. Zielke, N., Korzelius, J., van Straaten, M., Bender, K., Schuhknecht, G.F.P., Dutta, D., Xiang, J., and Edgar, B.A. (2014). Fly-FUCCI: A versatile tool for studying cell proliferation in complex tissues. Cell Rep. *7*, 588–598. https://doi.org/10.1016/j.celrep.2014.03.020.

47. Thacker, S.A., Bonnette, P.C., and Duronio, R.J. (2003). The contribution of E2F-regulated transcription to Drosophila PCNA gene function. Curr. Biol. *13*, 53–58. https://doi.org/10.1016/s0960-9822(02)01400-8.

48. Ding, A.X., Sun, G., Argaw, Y.G., Wong, J.O., Easwaran, S., and Montell, D.J. (2016). CasExpress reveals widespread and diverse patterns of cell survival of caspase-3 activation during development in vivo. Elife *5*, e10936. https://doi.org/10.7554/eLife.10936.

49. Li, M., Sun, S., Priest, J., Bi, X., and Fan, Y. (2019). Characterization of TNF-induced cell death in Drosophila reveals caspase- and JNK-dependent necrosis and its role in tumor suppression. Cell Death Dis. *10*, 613. https://doi.org/10.1038/s41419-019-1862-0.

50. Schott, S., Ambrosini, A., Barbaste, A., Benassayag, C., Gracia, M., Proag, A., Rayer, M., Monier, B., and Suzanne, M. (2017). A fluorescent toolkit for spatiotemporal tracking of apoptotic cells in living Drosophila tissues. Development *144*, 3840–3846. https://doi.org/10.1242/dev.149807.

51. Bardet, P.L., Kolahgar, G., Mynett, A., Miguel-Aliaga, I., Briscoe, J., Meier, P., and Vincent, J.P. (2008). A fluorescent reporter of caspase activity for live imaging. Proc. Natl. Acad. Sci. USA *105*, 13901–13905. https://doi.org/10.1073/pnas.0806983105.

52. Denton, D., and Kumar, S. (2015). Terminal Deoxynucleotidyl Transferase (TdT)-Mediated dUTP Nick-End Labeling (TUNEL) for Detection of Apoptotic Cells in Drosophila. Cold Spring Harb. Protoc. *2015*, 568–571. https://doi.org/10.1101/pdb.prot086199.

53. Baena-Lopez, L.A., Arthurton, L., Bischoff, M., Vincent, J.P., Alexandre, C., and McGregor, R. (2018). Novel initiator caspase reporters uncover previously unknown features of caspase-activating cells. Development *145*, dev170811. https://doi.org/10.1242/dev.170811.

54. Sun, G., Ding, X.A., Argaw, Y., Guo, X., and Montell, D.J. (2020). Akt1 and dCIZ1 promote cell survival from apoptotic caspase activation during regeneration and oncogenic overgrowth. Nat. Commun. *11*, 5726. https://doi.org/10.1038/s41467-020-19068-2.

55. Kurucz, E., Márkus, R., Zsámboki, J., Folkl-Medzihradszky, K., Darula, Z., Vilmos, P., Udvardy, A., Krausz, I., Lukacsovich, T., Gateff, E., et al. (2007). Nimrod, a putative phagocytosis receptor with EGF repeats in Drosophila plasmatocytes. Curr. Biol. *17*, 649–654. https://doi.org/10.1016/j.cub.2007.02.041.

56. Csordas, G., Gabor, E., and Honti, V. (2021). There and back again: The mechanisms of differentiation and transdifferentiation in Drosophila blood cells. Dev. Biol. *469*, 135–143. https://doi.org/10.1016/j.ydbio.2020.10.006.

57. Kondo, S., Senoo-Matsuda, N., Hiromi, Y., and Miura, M. (2006). DRONC coordinates cell death and compensatory proliferation. Mol. Cell Biol. *26*, 7258–7268. https://doi.org/10.1128/MCB.00183-06.

58. Muro, I., Berry, D.L., Huh, J.R., Chen, C.H., Huang, H., Yoo, S.J., Guo, M., Baehrecke, E.H., and Hay, B.A. (2006). The Drosophila caspase Ice is important for many apoptotic cell deaths and for spermatid individualization, a nonapoptotic process. Development *133*, 3305–3315. https://doi.org/10.1242/dev.02495.

59. Xu, D., Li, Y., Arcaro, M., Lackey, M., and Bergmann, A. (2005). The CARD-carrying caspase Dronc is essential for most, but not all, developmental cell death in Drosophila. Development *132*, 2125–2134. https://doi.org/10.1242/dev.01790.

60. Musashe, D.T., Purice, M.D., Speese, S.D., Doherty, J., and Logan, M.A. (2016). Insulin-like Signaling Promotes Glial Phagocytic Clearance of Degenerating Axons through Regulation of Draper. Cell Rep. *16*, 1838–1850. https://doi.org/10.1016/j.celrep.2016.07.022.

61. Yang, S.A., Portilla, J.M., Mihailovic, S., Huang, Y.C., and Deng, W.M. (2019). Oncogenic Notch Triggers Neoplastic Tumorigenesis in a Transition-Zone-like Tissue Microenvironment. Dev. Cell *49*, 461–472.e5. https://doi.org/10.1016/j.devcel.2019.03.015.

62. Laundrie, B., Peterson, J.S., Baum, J.S., Chang, J.C., Fileppo, D., Thompson, S.R., and McCall, K. (2003). Germline cell death is inhibited by P-element insertions disrupting the dcp-1/pita nested gene pair in Drosophila. Genetics *165*, 1881–1888. https://doi.org/10.1093/genetics/165.4.1881.

63. Siegrist, S.E., Haque, N.S., Chen, C.H., Hay, B.A., and Hariharan, I.K. (2010). Inactivation of both Foxo and reaper promotes long-term adult neurogenesis in Drosophila. Curr. Biol. *20*, 643–648. https://doi.org/10.1016/j.cub.2010.01.060.

64. Shinoda, N., Hanawa, N., Chihara, T., Koto, A., and Miura, M. (2019). Dronc-independent basal executioner caspase activity sustains Drosophila imaginal tissue growth. Proc. Natl. Acad. Sci. USA *116*, 20539–20544. https://doi.org/10.1073/pnas.1904647116.

65. Larsen, B.D., and Sørensen, C.S. (2017). The caspase-activated DNase: apoptosis and beyond. FEBS J. *284*, 1160–1170. https://doi.org/10.1111/febs.13970.

66. Enari, M., Sakahira, H., Yokoyama, H., Okawa, K., Iwamatsu, A., and Nagata, S. (1998). A caspase-activated DNase that degrades DNA during apoptosis, and its inhibitor ICAD. Nature *391*, 43–50. https://doi.org/10.1038/34112.

67. Ha, H.J., and Park, H.H. (2022). Molecular basis of apoptotic DNA fragmentation by DFF40. Cell Death Dis. *13*, 198. https://doi.org/10.1038/s41419-022-04662-7.

68. Choi, J.Y., Qiao, Q., Hong, S.H., Kim, C.M., Jeong, J.H., Kim, Y.G., Jung, Y.K., Wu, H., and Park, H.H. (2017). CIDE domains form functionally important higher-order assemblies for DNA fragmentation. Proc. Natl. Acad. Sci. USA *114*, 7361–7366. https://doi.org/10.1073/pnas.1705949114.

69. Mukae, N., Yokoyama, H., Yokokura, T., Sakoyama, Y., Sakahira, H., and Nagata, S. (2000). Identification and developmental expression of inhibitor of caspase-activated DNase (ICAD) in Drosophila melanogaster. J. Biol. Chem. *275*, 21402–21408. https://doi.org/10.1074/jbc.M909611199.

70. Lee, P.T., Zirin, J., Kanca, O., Lin, W.W., Schulze, K.L., Li-Kroeger, D., Tao, R., Devereaux, C., Hu, Y., Chung, V., et al. (2018). A gene-specific T2A-GAL4 library for Drosophila. Elife *7*, e35574. https://doi.org/10.7554/eLife.35574.

71. Tarayrah-Ibraheim, L., Maurice, E.C., Hadary, G., Ben-Hur, S., Kolpakova, A., Braun, T., Peleg, Y., Yacobi-Sharon, K., and Arama, E. (2021). DNase II mediates a parthanatos-like developmental cell death pathway in Drosophila primordial germ cells. Nat. Commun. *12*, 2285. https://doi.org/10.1038/s41467-021-22622-1.

72. Yacobi-Sharon, K., Namdar, Y., and Arama, E. (2013). Alternative germ cell death pathway in Drosophila involves HtrA2/Omi, lysosomes, and a caspase-9 counterpart. Dev. Cell *25*, 29–42. https://doi.org/10.1016/j.devcel.2013.02.002.

73. Evans, C.J., Olson, J.M., Mondal, B.C., Kandimalla, P., Abbasi, A., Abdusamad, M.M., Acosta, O., Ainsworth, J.A., Akram, H.M., Albert, R.B., et al. (2021). A Functional Genomics Screen Identifying Blood Cell Development Genes in Drosophila by Undergraduates Participating in a Course-Based Research Experience. G3 (Bethesda) *11*. https://doi.org/10.1093/g3journal/jkaa028.

74. Purice, M.D., Speese, S.D., and Logan, M.A. (2016). Delayed glial clearance of degenerating axons in aged Drosophila is due to reduced PI3K/Draper activity. Nat. Commun. *7*, 12871. https://doi.org/10.1038/ncomms12871.

75. Leevers, S.J., Weinkove, D., MacDougall, L.K., Hafen, E., and Waterfield, M.D. (1996). The Drosophila phosphoinositide 3-kinase Dp110 promotes cell growth. EMBO J. *15*, 6584–6594.

76. Santabarbara-Ruiz, P., Esteban-Collado, J., Perez, L., Viola, G., Abril, J.F., Milan, M., Corominas, M., and Serras, F. (2019). Ask1 and Akt act synergistically to promote ROS-dependent regeneration in Drosophila. PLoS Genet. *15*, e1007926. https://doi.org/10.1371/journal.pgen.1007926.

77. Santabarbara-Ruiz, P., Lopez-Santillan, M., Martinez-Rodriguez, I., Binagui-Casas, A., Perez, L., Milan, M., Corominas, M., and Serras, F. (2015). ROS-Induced JNK and p38 Signaling Is Required for Unpaired Cytokine Activation during Drosophila Regeneration. PLoS Genet. *11*, e1005595. https://doi.org/10.1371/journal.pgen.1005595.

78. Toshniwal, A.G., Gupta, S., Mandal, L., and Mandal, S. (2019). ROS Inhibits Cell Growth by Regulating 4EBP and S6K, Independent of TOR,

during Development. Dev. Cell *49*, 473–489.e9. https://doi.org/10.1016/j.devcel.2019.04.008.

79. Uhlirova, M., and Bohmann, D. (2006). JNK- and Fos-regulated Mmp1 expression cooperates with Ras to induce invasive tumors in Drosophila. EMBO J. *25*, 5294–5304. https://doi.org/10.1038/sj.emboj.7601401.
80. Chatterjee, N., and Bohmann, D. (2012). A versatile PhiC31 based reporter system for measuring AP-1 and Nrf2 signaling in Drosophila and in tissue culture. PLoS One *7*, e34063. https://doi.org/10.1371/journal.pone.0034063.
81. Girard, J.R., Goins, L.M., Vuu, D.M., Sharpley, M.S., Spratford, C.M., Mantri, S.R., and Banerjee, U. (2021). Paths and pathways that generate cell-type heterogeneity and developmental progression in hematopoiesis. Elife *10*, e67516. https://doi.org/10.7554/eLife.67516.
82. Sykiotis, G.P., and Bohmann, D. (2008). Keap1/Nrf2 signaling regulates oxidative stress tolerance and lifespan in Drosophila. Dev. Cell *14*, 76–85. https://doi.org/10.1016/j.devcel.2007.12.002.
83. Tattikota, S.G., Cho, B., Liu, Y., Hu, Y., Barrera, V., Steinbaugh, M.J., Yoon, S.H., Comjean, A., Li, F., Dervis, F., et al. (2020). A single-cell survey of Drosophila blood. Elife *9*, e54818. https://doi.org/10.7554/eLife.54818.
84. Gyoergy, A., Roblek, M., Ratheesh, A., Valoskova, K., Belyaeva, V., Wachner, S., Matsubayashi, Y., Sánchez-Sánchez, B.J., Stramer, B., and Siekhaus, D.E. (2018). Tools Allowing Independent Visualization and Genetic Manipulation of Drosophila melanogaster Macrophages and Surrounding Tissues. G3 (Bethesda) *8*, 845–857. https://doi.org/10.1534/g3.117.300452.
85. Weavers, H., Evans, I.R., Martin, P., and Wood, W. (2016). Corpse Engulfment Generates a Molecular Memory that Primes the Macrophage Inflammatory Response. Cell *165*, 1658–1671. https://doi.org/10.1016/j.cell.2016.04.049.
86. Nagarkar-Jaiswal, S., DeLuca, S.Z., Lee, P.T., Lin, W.W., Pan, H., Zuo, Z., Lv, J., Spradling, A.C., and Bellen, H.J. (2015). A genetic toolkit for tagging intronic MiMIC containing genes. Elife *4*, e08469. https://doi.org/10.7554/eLife.08469.
87. Melcarne, C., Ramond, E., Dudzic, J., Bretscher, A.J., Kurucz, É., Andó, I., and Lemaitre, B. (2019). Two Nimrod receptors, NimC1 and Eater, synergistically contribute to bacterial phagocytosis in Drosophila melanogaster. FEBS J. *286*, 2670–2691. https://doi.org/10.1111/febs.14857.
88. Machour, F.E., and Ayoub, N. (2020). Transcriptional Regulation at DSBs: Mechanisms and Consequences. Trends Genet. *36*, 981–997. https://doi.org/10.1016/j.tig.2020.01.001.
89. Cho, B., Yoon, S.H., Lee, D., Koranteng, F., Tattikota, S.G., Cha, N., Shin, M., Do, H., Hu, Y., Oh, S.Y., et al. (2020). Single-cell transcriptome maps of myeloid blood cell lineages in Drosophila. Nat. Commun. *11*, 4483. https://doi.org/10.1038/s41467-020-18135-y.
90. Arthurton, L., Nahotko, D.A., Alonso, J., Wendler, F., and Baena-Lopez, L.A. (2020). Non-apoptotic caspase activation preserves Drosophila intestinal progenitor cells in quiescence. EMBO Rep. *21*, e48892. https://doi.org/10.15252/embr.201948892.
91. Amcheslavsky, A., Lindblad, J.L., and Bergmann, A. (2020). Transiently "Undead" Enterocytes Mediate Homeostatic Tissue Turnover in the Adult Drosophila Midgut. Cell Rep. *33*, 108408. https://doi.org/10.1016/j.celrep.2020.108408.
92. Fujisawa, Y., Shinoda, N., Chihara, T., and Miura, M. (2020). ROS Regulate Caspase-Dependent Cell Delamination without Apoptosis in the Drosophila Pupal Notum. iScience *23*, 101413. https://doi.org/10.1016/j.isci.2020.101413.
93. White, K., Arama, E., and Hardwick, J.M. (2017). Controlling caspase activity in life and death. PLoS Genet. *13*, e1006545. https://doi.org/10.1371/journal.pgen.1006545.
94. Ojha, S., and Tapadia, M.G. (2022). Nonapoptotic role of caspase-3 in regulating Rho1GTPase-mediated morphogenesis of epithelial tubes of Drosophila renal system. Dev. Dynam. *251*, 777–794. https://doi.org/10.1002/dvdy.437.
95. Jacquel, A., Benikhlef, N., Paggetti, J., Lalaoui, N., Guery, L., Dufour, E.K., Ciudad, M., Racoeur, C., Micheau, O., Delva, L., et al. (2009). Colony-stimulating factor-1-induced oscillations in phosphatidylinositol-3 kinase/AKT are required for caspase activation in monocytes undergoing differentiation into macrophages. Blood *114*, 3633–3641. https://doi.org/10.1182/blood-2009-03-208843.
96. Nano, M., Mondo, J.A., Harwood, J., Balasanyan, V., and Montell, D.J. (2023). Cell survival following direct executioner-caspase activation. Proc. Natl. Acad. Sci. USA *120*, e2216531120. https://doi.org/10.1073/pnas.2216531120.
97. Yang, J.Y., Michod, D., Walicki, J., Murphy, B.M., Kasibhatla, S., Martin, S.J., and Widmann, C. (2004). Partial cleavage of RasGAP by caspases is required for cell survival in mild stress conditions. Mol. Cell Biol. *24*, 10425–10436. https://doi.org/10.1128/MCB.24.23.10425-10436.2004.
98. Shemorry, A., Harnoss, J.M., Guttman, O., Marsters, S.A., Kőműves, L.G., Lawrence, D.A., and Ashkenazi, A. (2019). Caspase-mediated cleavage of IRE1 controls apoptotic cell commitment during endoplasmic reticulum stress. Elife *8*, e47084. https://doi.org/10.7554/eLife.47084.
99. Cho, U.H., and Hetzer, M.W. (2023). Caspase-mediated nuclear pore complex trimming in cell differentiation and endoplasmic reticulum stress. Elife *12*. https://doi.org/10.7554/eLife.89066.
100. Al-Khalaf, M.H., Blake, L.E., Larsen, B.D., Bell, R.A., Brunette, S., Parks, R.J., Rudnicki, M.A., McKinnon, P.J., Jeffrey Dilworth, F., and Megeney, L.A. (2016). Temporal activation of XRCC1-mediated DNA repair is essential for muscle differentiation. Cell Discov. *2*, 15041. https://doi.org/10.1038/celldisc.2015.41.
101. Benada, J., Alsowaida, D., Megeney, L.A., and Sørensen, C.S. (2023). Self-inflicted DNA breaks in cell differentiation and cancer. Trends Cell Biol. *33*, 850–859. https://doi.org/10.1016/j.tcb.2023.03.002.
102. Spencer, S.L., and Sorger, P.K. (2011). Measuring and modeling apoptosis in single cells. Cell *144*, 926–939. https://doi.org/10.1016/j.cell.2011.03.002.
103. Larsen, B.D., Benada, J., Yung, P.Y.K., Bell, R.A.V., Pappas, G., Urban, V., Ahlskog, J.K., Kuo, T.T., Janscak, P., Megeney, L.A., et al. (2022). Cancer cells use self-inflicted DNA breaks to evade growth limits imposed by genotoxic stress. Science *376*, 476–483. https://doi.org/10.1126/science.abi6378.
104. Dehingia, B., Milewska, M., Janowski, M., and Pękowska, A. (2022). CTCF shapes chromatin structure and gene expression in health and disease. EMBO Rep. *23*, e55146. https://doi.org/10.15252/embr.202255146.
105. Puc, J., Aggarwal, A.K., and Rosenfeld, M.G. (2017). Physiological functions of programmed DNA breaks in signal-induced transcription. Nat. Rev. Mol. Cell Biol. *18*, 471–476. https://doi.org/10.1038/nrm.2017.43.
106. Simmons, J.R., Kemp, J.D.J., and Labrador, M. (2023). 10.1101/2023.05.31.543180.
107. Bekkering, S., Domínguez-Andrés, J., Joosten, L.A.B., Riksen, N.P., and Netea, M.G. (2021). Trained Immunity: Reprogramming Innate Immunity in Health and Disease. Annu. Rev. Immunol. *39*, 667–693. https://doi.org/10.1146/annurev-immunol-102119-073855.
108. McPhee, C.K., Logan, M.A., Freeman, M.R., and Baehrecke, E.H. (2010). Activation of autophagy during cell death requires the engulfment receptor Draper. Nature *465*, 1093–1096. https://doi.org/10.1038/nature09127.
109. Kamiyama, R., Banzai, K., Liu, P., Marar, A., Tamura, R., Jiang, F., Fitch, M.A., Xie, J., and Kamiyama, D. (2021). Cell-type-specific, multicolor labeling of endogenous proteins with split fluorescent protein tags in Drosophila. Proc. Natl. Acad. Sci. USA *118*, e2024690118. https://doi.org/10.1073/pnas.2024690118.

STAR★METHODS

KEY RESOURCES TABLE

REAGENT or RESOURCE	SOURCE	IDENTIFIER
**Antibodies**		
Mouse-histone 2A gamma variant, phosphorylated (γH2Av)	DSHB	Cat# UNC93–5.2.1-s; RRID: AB_2618077
Mouse-hindsight	DSHB	Cat# 1G9-c; RRID: AB_528278
Mouse-Mmp1 catalytic domain	DSHB	Cat# 3A6B4; RRID: AB_579780
Mouse-Mmp1 catalytic domain	DSHB	Cat# 3B8D12; RRID: AB_579781
Mouse-Mmp1 catalytic domain	DSHB	Cat# 5H7B11; RRID: AB_579779
Mouse-Draper	DSHB	Cat# 5D14-s; RRID: AB_2618105
Rabbit-Cleaved Drosophila Dcp-1 (Asp215)	CST	Cat# 9578S; RRID: AB_2721060
Rabbit-Phospho-Akt (Ser473)	CST	Cat# 9271; RRID: AB_329825
Rabbit-Phospho-ATM/ATR Substrate Motif [(pS/pT) QG] MultiMab™	CST	Cat# 6966S; RRID: AB_10949894
Rabbit-pChk1	Abcam	Cat# Ab47318; RRID: AB_869137
Rabbit-Histone H2AvD phosphoS137 (γH2Av)	Rockland	Cat# 600-401-914; RRID: AB_828383
Rabbit-Anti-GFP	Invitrogen	Cat# A11122; RRID: AB_221569
Mouse-P1 (NimC1)	Istavan Ando	N/A
donkey anti-mouse Alexa Fluor 555	Invitrogen	Cat# A31570; RRID: AB_2536180
goat anti-mouse Alexa Fluor 647	Invitrogen	Cat# A21050; RRID: AB_2535718
goat anti-mouse Cy3	Jackson Immuno Research	Cat# 115-165-003; RRID: AB_2338680
donkey anti-rabbit Alexa Fluor 555	Invitrogen	Cat# A31572; RRID: AB_162543
Goat-rabbit Alexa Fluor 647	Invitrogen	Cat# A32733; RRID: AB_2866492
**Bacterial and virus strains**		
E. coli DH10B containing p70rg plasmid	Addgene	Cat# 17827
**Chemicals, peptides, and recombinant proteins**		
Roche *In Situ* Cell Death Detection Kit, TMR red	Sigma	Cat# 12156792910
DAPI (4′,6-Diamidino-2 Phenylindole, Dihydrochloride)	Invitrogen	Cat# D1306
Dihydroethidium (Hydroethidine)	Invitrogen	Cat# D11347
4% paraformaldehyde (PFA)	Thermo Fisher Scientific	Cat# 28908
DMSO	Sigma	Cat# D12345
L-Arabinose	Sigma	Cat# A81906
Ampicillin	Sigma	Cat# A5354
Insulin solution	Sigma	Cat# I0516
TO-PRO-3 Iodide (642/661)-1mM solution in DMSO	Thermo Fisher Scientific	Cat# T3605
Digoxigenin-11-dUTP, alkali-labile	Sigma	Cat# 11573152910
Anti-digoxigenin-Rhodamine, Fab fragments	Sigma	Cat# 11207750910
Schneider's *Drosophila* medium (1X)	Gibco	Cat# 21720024
Deoxynucleotide Set, 100mM	Sigma	Cat# DNTP100-KT
DABCO (1,4-diazabicyclo [2.2. 2]octane)	Sigma	Cat# D27802
DNA polymerase I	New England Biolabs	Cat# M0209S
Fetal Bovine Serum	Himedia	Cat# RM9955
Luria Bertani broth	Himedia	Cat# M1245
Triton X-100	Sigma	Cat# T8787
Bovine Serum Albumin	SRL	Cat# 83803
Thiomersal	SRL	Cat# 85090

(*Continued on next page*)

CellPress
OPEN ACCESS

Continued

REAGENT or RESOURCE	SOURCE	IDENTIFIER
Deoxycholic Acid	Sigma	Cat# D-6750
Halocarbon oil	Sigma	Cat# H8898
TRI Reagent	Sigma	Cat#T9424
DNase I Solution	Thermo Fisher Scientific	Cat# 89836
Reaction Buffer with $MgCl_2$ for DNase I (10X)	Thermo Fisher Scientific	Cat#B43
Random Hexamer Primer	Thermo Fisher Scientific	Cat#SO142
Ethylenediaminetetraacetic acid (EDTA) (0.5 M), pH 8.0	Thermo Fisher Scientific	Cat# R1021
SYBR Green qPCR Master Mix	Genetix	Cat#PKG025-A
RevertAid Reverse transcriptase	Thermo Fisher Scientific	Cat#EP0442
Deposited data		
Raw and analyzed graph data	This paper	https://data.mendeley.com/preview/kfr247v7sn?a=241e7adf-3b8d-4ab4-8e86-43a0dcfc058a
Experimental models: Organisms/strains		
D. melanogaster. dome^{MESO}-Gal4, UAS-2xEGFP	Utpal Banerjee	N/A
D. melanogaster. Hml$^{\Delta}$-Gal4, UAS-2xEGFP	Utpal Banerjee	N/A
D. melanogaster. CHIZ-Gal4, UAS-mGFP	Utpal Banerjee	N/A
D. melanogaster. GTRACE^{LTO}	Utpal Banerjee	N/A
D. melanogaster. w^{1118}	Utpal Banerjee	N/A
D. melanogaster. Nup98-GFP	Utpal Banerjee	N/A
D. melanogaster. e33c-Gal4	Maneesha Inamdar	N/A
D. melanogaster. gstD-GFP	Dirk Bohmann	N/A
D. melanogaster. RPA70-GFP	Eric Wieschaus	N/A
D. melanogaster. UAS-VC3Ai	Magali Suzanne	N/A
D. melanogaster. UAS-GC3Ai	Magali Suzanne	RRID: BDSC_84346
D. melanogaster. Dronc^{I24}	Andreas Bergmann	N/A
D. melanogaster. Dronc^{I29}	Andreas Bergmann	N/A
D. melanogaster. Drice$^{\Delta 1}$	Bruce A. Hay	N/A
D. melanogaster. Drice^{2c8}	Masayuki Miura	N/A
D. melanogaster. UAS-Drice ^{RNAi}; UAS-Dcp-1^{RNAi}	Masayuki Miura	N/A
D. melanogaster. UAS-Dronc ^{RNAi}	Masayuki Miura	N/A
D. melanogaster. UAS-miRHG	Iswar K. Hariharan	N/A
D. melanogaster. UAS-Ask1^{S83A}	Florenci Serras	N/A
D. melanogaster. Dcp-1^{Prev1}	BDSC	RRID: BDSC_63814
D. melanogaster. UAS-Drep4 ^{RNAi}	BDSC	RRID: BDSC_67883
D. melanogaster. UAS-Drep1 ^{RNAi}	BDSC	RRID: BDSC_65944
D. melanogaster. UAS-Drep1 ^{RNAi}	VDRC	RRID: FlyBase_ FBgn0027578; v8357
D. melanogaster. Drep4-Gal4	BDSC	RRID: BDSC_80624
D. melanogaster. Drpr-GFP	BDSC	RRID: BDSC_63184
D. melanogaster. UAS-mCD8::RFP	BDSC	RRID: BDSC_27398
D. melanogaster. CasExpress	BDSC	RRID: BDSC_65419
D. melanogaster. CasExpress^{mutant}	BDSC	RRID: BDSC_65420
D. melanogaster. UAS- RedStinger	BDSC	RRID: BDSC_8546
D. melanogaster. UAS-Apoliner	BDSC	RRID: BDSC_32121
D. melanogaster. UAS-Apoliner	BDSC	RRID: BDSC_32123
D. melanogaster. L-Caspase	BDSC	RRID: BDSC_92353
D. melanogaster. UAS-lexAop 2xmRFP	BDSC	RRID: BDSC_29956

(*Continued on next page*)

Continued

REAGENT or RESOURCE	SOURCE	IDENTIFIER
D. melanogaster. Ubi FRT-STOP-FRT GFP	BDSC	RRID: BDSC_32251
D. melanogaster. Lex-Aop-Flp	BDSC	RRID: BDSC_55819
D. melanogaster. Dronc-DBS	BDSC	RRID: BDSC_83129
D. melanogaster. PCNA-GFP	BDSC	RRID: BDSC_25749
D. melanogaster. UAS-p35	BDSC	*RRID: BDSC_5072*
D. melanogaster. UAS-Ras^{DN}	BDSC	RRID: BDSC_4845
D. melanogaster. UAS-Pnt ^{RNAi}	BDSC	RRID: BDSC_31936
D. melanogaster. UAS-Hh ^{RNAi}	BDSC	RRID: BDSC_25794
D. melanogaster. UAS-Pvr ^{RNAi}	BDSC	RRID: BDSC_37520
D. melanogaster. UAS-wg ^{RNAi}	BDSC	RRID: BDSC_31310
D. melanogaster. UAS-Stat92E ^{RNAi}	BDSC	RRID: BDSC_33637
D. melanogaster. UAS-Egfr ^{RNAi}	BDSC	RRID: BDSC_60012
D. melanogaster. UAS-Akt ^{RNAi}	BDSC	RRID: BDSC_33615
D. melanogaster. UAS-Akt ^{RNAi}	BDSC	RRID: BDSC_31701
D. melanogaster. UAS-PI3K^{DN}	BDSC	RRID: BDSC_8288
D. melanogaster. UAS-PI3K^{CAAX}	BDSC	RRID: BDSC_25908
D. melanogaster. UAS-InR ^{RNAi}	BDSC	RRID: BDSC_31037
D. melanogaster. UAS-InR ^{RNAi}	VDRC	992; RRID: FlyBase_FBgn0051607
D. melanogaster. UAS-Ask1 ^{RNAi}	BDSC	RRID: BDSC_35331
D. melanogaster. UAS-Ask1 ^{RNAi}	BDSC	RRID: BDSC_32464
D. melanogaster. DNaseII^{lo}	BDSC	RRID: BDSC_1042
D. melanogaster. EndoG^{MB07150}	BDSC	RRID: BDSC_26072
D. melanogaster. puc^{E69}	BDSC	RRID: BDSC_98329
D. melanogaster. UAS-JNK^{RNAi}	BDSC	RRID: BDSC_31323
D. melanogaster. TRE-DsRed	BDSC	RRID: BDSC_59012
D. melanogaster. srp-mCherry	BDSC	RRID: BDSC_78361
D. melanogaster. UAS-FUCCI	BDSC	RRID: BDSC_55121
D. melanogaster. GMR-rpr	BDSC	RRID: BDSC_5773
Oligonucleotides		
Drep1 (Forward): 5′-AAACAAAGCCATG GAGACTGCAG-3′	This paper	N/A
Drep1 (Reverse): 5′AGACAGCCTTCTTA ATGTTGCGTG-3′	This paper	N/A
Drep4 (Forward): 5′-CCTGCTCATCGGTTGCGAC-3′	This paper	N/A
Drep4 (Reverse): 5′-GTTTCCTCGTCGCCCAAGTG-3′	This paper	N/A
Rp49 (Forward): 5′-TTGAGAACGCAGGCGACC GT-3′	This paper	N/A
Rp49 (Reverse): 5′-CGTCTCCTCCAAGAAGCGCAAG-3′	This paper	N/A
Software and algorithms		
ImageJ	NIH	https://imagej.nih.gov/ij/
Prism 9	GraphPad	https://www.graphpad.com/scientific-software/prism/
Zen Software Version 3.4	Zeiss	https://www.zeiss.com/microscopy/us/products/microscope-software/zen.html
Adobe Photoshop 2021	Adobe	version 22.4.2
Adobe Illustrator cc 2018	Adobe	version 22.1
Microsoft Word, Excel, PowerPoint	Microsoft 2019	Microsoft 2019

CellPress
OPEN ACCESS

RESOURCE AVAILABILITY

Lead contact

Further information and requests for resources and reagents should be directed to and will be fulfilled by the lead contact, Bama Charan Mondal (bamacharan@bhu.ac.in).

Materials availability

This study did not generate new reagents.

Data and code availability

- Raw and analyzed graph data generated in this work have been deposited at Mendeley Data Repository and are publicly available as of the date of publication. Accession numbers are listed in the key resources table.
- This paper does not report the original code.
- Any additional information required to reanalyze the data reported in this paper is available from the lead contact upon request.

EXPERIMENTAL MODEL AND STUDY PARTICIPANT DETAILS

Drosophila stocks were cultured using standard fly medium comprising 46 g/L cornmeal, 45 g/L sucrose, 18 g/L yeast extract, 7 g/L agar, supplemented with 3 mL/L propionic acid, and 3 g/L *p*-hydroxybenzoic acid methyl ester. All stocks were maintained at room temperature or 18°C, and genetic crosses using the GAL4/UAS system were maintained at 29°C on a 12 h light/12 h dark cycle. The following *Drosophila* stocks were used for this study: *CHIZ-GAL4 UAS-mCD8::GFP*[40], *Hml*$^{\Delta}$*-Gal4 UAS-2xEGFP, dome*^{MESO}*-Gal4 UAS-2xEGFP, Nup98-GFP, w*^{1118}*, UAS-RedStinger* (BL8546), and *UAS-GTRACE*^{LTO} (BL28282) were from Utpal Banerjee's lab. The following fly lines were obtained from Bloomington *Drosophila* Stock Center (BDSC): *UAS-wg*^{RNAi} (BL31310), *UAS-Hh*^{RNAi} (BL25794), *UAS-Ras*^{DN} (BL4845), *UAS-Pvr*^{RNAi} (BL37520), *UAS-Pnt*^{RNAi} (BL31936), *UAS-stat92E*^{RNAi} (BL33637), *UAS-Egfr*^{RNAi} (BL60012), *GMR-rpr* (BL5773), *UAS-Akt*^{RNAi} (BL33615 and BL31701), *UAS-Ask1*^{RNAi} (BL35331 and BL32464), *UAS-Drep1*^{RNAi} (BL65944), *UAS-Drep4*^{RNAi} (BL67883), *UAS-InR*^{RNAi} (BL31037), *UAS-GC3Ai* (BL84346), *srp-mCherry* (BL78361),[84] *DNaseII*^{lo} (BL1042), *Dronc-DBS* (BL83129), *Ubi-p63-(FRT-STOP-FRT-Stinger*) (BL32250), *L-Caspase* (BL92353),[54] *LexAop-Flp* (BL55820), *PCNA-GFP* (BL25749), *UAS-Apoliner* (BL32121 and BL32123), *EndoG*^{MB07150} (BL26072)*, Drpr-GFP* (BL63184), *UAS-PI3K*^{DN} (BL8288), *UAS-PI3K*^{CAAX} (BL25908), *Dcp-1*^{Prev1} (BL63814)*, puc[E69]* (BL98329)*, UAS-JNK*^{RNAi} (BL31323), *TRE-DsRed* (BL59012), *UAS-lexAop-2xmRFP* (BL29956), *UAS-p35* (BL5072), *CasExpress*^{mutant} (BL65419), *CasExpress* (BL65420),[48] *UAS-mCD8::RFP* (BL27398), *Drep4-Gal4* (BL80624),[70] *UAS-FUCCI* (BL55121). Flies from Vienna Drosophila Stock Center: *UAS-Drep1*^{RNAi} (v8357) and *UAS-InR* ^{RNAi} (v992). The following stocks were kind gifts from different labs: *Dronc*^{I29}, *Dronc*^{I24} (Andreas Bergmann),[59] *Drice*$^{\Delta 1}$ (Bruce A Hay)[58], *Drice*^{2c8}*, UAS-Drice*^{RNAi}*; UAS-Dcp-1*^{RNAi} and *UAS-Dronc*^{RNAi} (Masayuki Miura)[57,64]; *UAS-miRHG* (Iswar K. Hariharan)[63]; *UAS-Ask1*^{S83A} (Florenci Serras)[76]; *RPA70-GFP* (Eric Wieschaus)[45]; *e33c-Gal4* (Maneesha Inamdar),[26] *gstD-GFP* (Dirk Bohmann),[82] *UAS-GC3Ai, UAS-VC3Ai* (Magali Suzanne).[50]

The lymph glands of *Drosophila melanogaster* at wandering third-instar larval stage were used in most of the experiments. In some of the specific experiments, early stages of lymph glands and embryos were used, and their exact age were mentioned. The lymph glands and embryos of both sexes were used and our study cannot differentiate between the two. For the genetic crosses, one-day-old virgin females and males after eclosion were used.

METHOD DETAILS

Drosophila lymph gland dissection and immunostaining

Lymph glands were dissected from wandering third-instar larvae on a silicon dissecting plate. The head complex, comprising the lymph gland, brain, eye-antennal disc, and mouth hook, was isolated in chilled 1X PBS (phosphate buffer saline). The tissues were then immersed in a fixative solution, 4% paraformaldehyde (PFA, Thermo Fisher Scientific, Cat# 28908) in 1X PBS for 30 min and washed 3 times for 10 min each with wash buffer (0.3% Triton X-100 in 1X PBS). Samples were incubated with blocking solution (0.1% Triton X-100, 0.1% BSA, 10% FBS, 0.1% deoxycholate, 0.02% thiomersal) for 2 h at room temperature (or in the case of Draper staining 24 h at 4°C) and then incubated with primary antibody overnight at 4°C. Samples were washed with a wash buffer thrice, then incubated with a blocking solution for 2 h at RT and incubated with a secondary antibody for 2 h at RT. Following the incubation with the secondary antibody, the tissues were subjected to three washes in 0.3% PBST. Subsequently, counterstaining was performed using DAPI (4′,6-Diamidino-2-Phenylindole, Dihydrochloride, Thermo Fisher Scientific, Cat# D1306) (1 μg/mL) and To-Pro-3 to visualize the nuclei of tissues. Samples were then washed three times and finally immersed in DABCO (1,4-diazabicyclo [2.2.2] octane, Sigma, Cat# D27802, 2.5% DABCO in 70% glycerol made in 1X PBS) until they were mounted on glass slides.

All antibodies were diluted in a blocking buffer. The following primary antibodies were used: mouse anti-γH2Av (1:1000, UNC93–5.2.1-s, DSHB),[37] mouse anti-Hnt (1:100, 1G9c, DSHB), mouse anti-MMP1 catalytic domain (a cocktail of three antibodies at dilution 1:10, 3A6B4, 3B8D12, 5H7B11, DSHB), mouse anti-Draper (1:10, 5D14-s, DSHB),[108] rabbit anti-cleaved Dcp-1 (1:100, 9578S, CST), rabbit anti-*p*-Akt (S473) (1:100, 9271, CST), rabbit anti-phospho-ATM/ATR Substrate Motif (1; 100, 6966S, CST), rabbit-pChk1

(1:100, Ab47318, Abcam), rabbit anti-histone H2AvD phosphoS137 (1:100, 600-401-914, Rockland), mouse anti-P1 (1:100, Istvan Ando)[55] and rabbit anti-GFP(1:300, A11122, Invitrogen). The secondary antibodies used for the immunohistochemistry are as follows: donkey anti-mouse Alexa Fluor 555 (A31570), goat anti-mouse Alexa Fluor 647 (A21050), donkey anti-rabbit Alexa Fluor 555 (A31572) and Goat anti-rabbit Alexa Fluor 647 (A32733) from Invitrogen and goat anti-mouse Cy3 (AB_2338680) from Jackson Scientific. All the secondary antibodies were used in 1:200 dilutions.

Dihydroethidium (DHE) staining for ROS

DHE staining Reactive Oxygen Species (ROS) was done as described in Owusu-Ansah and Banerjee, 2009.[35] Briefly, the lymph gland was isolated in Schneider's *Drosophila* medium (Gibco, Cat# 21720024) at room temperature. The DHE (Dihydroethidium) dye (Invitrogen Molecular Probes, Cat# D11347) was prepared by reconstituting it in anhydrous DMSO (Sigma, Cat# D12345). The reconstituted DHE dye was dissolved in Schneider's medium to achieve a final 30 μM concentration. Subsequently, the tissues were incubated in DHE dye for 5 min at room temperature, followed by three washes for 5 min each with Schneider's medium. Finally, the tissues were mounted in DABCO, and images were acquired immediately.

Nick translation

The lymph glands were dissected in chilled 1X PBS, fixed in 4% paraformaldehyde for 30 min, and washed thrice for 10 min each with a wash buffer. Following these washes, tissues were washed with PBS supplemented with magnesium chloride (0.5mM) for 10 min each. The samples were transferred to PCR tubes and placed in a thermocycler at 37°C for 1 h. During this time, they were immersed in a reaction mixture consisting of 40 units/mL of *E. coli* DNA polymerase I (NEB, cat# M0209S), 50μM dATP, 50μM dGTP, 50μM dCTP, 35μM dTTP (Deoxynucleotide Set, 100mM, Sigma, Cat# DNTP100-KT), and 15μM DIG-11-dUTP (Digoxigenin-11-dUTP, alkali-labile, Sigma, Cat# 1157315291) in a 1X DNA polymerase reaction buffer. Following incubation, the samples were washed twice with wash buffer. They were then incubated for 2-h incubation with a blocking solution at room temperature and subsequently incubated with anti-digoxigenin-Rhodamine (Anti-digoxigenin-Rhodamine, Fab fragments, Sigma, Cat# 11207750910) (0.5 μg/mL) in the blocking solution for 2 h at room temperature. After incubation with anti-digoxigenin-rhodamine, the tissues underwent additional washes with wash buffer, and finally, the samples were stained with DAPI and mounted in DABCO mounting medium.[42] For the Nick translation assay, *Drosophila* eye discs harboring the GMR/+ genotype were employed as a wild-type control, GMR-rpr/+ as a positive control, and a second set of the w^{1118} genotype incubated without DNA polymerase I as a negative control.

TUNEL staining

The lymph glands were isolated in cold PBS, fixed in 4% paraformaldehyde at room temperature for 30 min, and washed 3 times with 0.3% PBST. TUNEL (terminal deoxynucleotidyl transferase-mediated deoxyuridine triphosphate nick-end labeling) staining was performed using *In Situ* Cell Death Detection Kit, TMR Red (Sigma, cat# 12156792910) according to the manufacturer's protocol.[28] *Drosophila* eye discs with the w^{1118} genotype were used as a wild-type control, *GMR-rpr/+* as a positive control for cell death, and another set of the same genotype incubated without enzyme used as a negative control was utilized for control TUNEL staining.

Drosophila larval staging

For synchronization, flies were allowed to lay embryos for 12 h on egg-laying plates. After 12 h of egg collection, these embryos were incubated at 25°C for 12 h. Following this incubation, hatched larvae were removed from the plate using a paintbrush, leaving behind unhatched embryos. The remaining unhatched embryos were incubated for 30 min at 25°C. The newly hatched larvae were carefully transferred to fresh vials of normal laboratory food and transferred to a 29°C incubator.[28] Different staged larvae at 38 h after larval hatching (ALH), 48h ALH, and 74h ALH were collected for γH2Av staining. Lymph glands were isolated, and immunostaining was performed as described in the immunostaining section.

Circulating blood cells counting

Third instar (L3) wandering larvae of different genotypes were bled in 20μL PBS on a clean coverslip, and hemocytes were allowed to adhere to the coverslip for 30 min. The PBS was carefully removed, and the cells were fixed with 4% PFA for 30 min. Following fixation, hemocytes were washed twice with PBS, stained with DAPI, and subjected to additional PBS washes.[39] The prepared samples were then mounted on clean slides. Using a Zeiss LSM-900 confocal microscope with 10X and 20× objectives, three random images were captured for each larval bleeding sample, encompassing GFP and DAPI channels. The number of DAPI- and GFP-positive hemocytes from each image was quantified manually using ImageJ.

Circulating hemocytes immunostaining

Third-instar (L3) wandering larvae were bled in 20μL PBS on a coverslip, and hemocytes were allowed to adhere to the coverslip for 30 min. The PBS was removed, and the cells were fixed with 4% PFA for 30 min. After fixation, immunostaining was performed similarly to lymph gland immunostaining, as described earlier.[39]

Phagocytic assay of circulating hemocytes

RFP-expressing *E. coli* (Addgene Cat# 17827) bacterial culture obtained from overnight culture in LB broth (Luria Bertani broth, HIMEDIA, Cat# M1245) supplemented with 0.2% L-Arabinose (Sigma, Cat# A81906) and 100 μg/mL Ampicillin (Sigma, Cat# A5354), was taken in a clean microcentrifuge tube. Bacteria were precipitated using centrifugation, and precipitated bacteria were washed with PBS. After washing, the bacteria precipitate was suspended in 100μL of autoclaved PBS. 1 μL of this suspension was used in each experiment. Phagocytosis assay was conducted using circulatory hemocytes isolated from wandering third-instar larvae.[87] These hemocytes were collected by bleeding the larvae onto a coverslip, where they came into contact with RFP-expressing *E. coli* suspended in autoclaved PBS. After a 10 min incubation in a humid chamber, the solution was removed, and the hemocytes were fixed using a 4% PFA fixative solution for 30 min. Following fixation, cells were washed with PBS two times, 10 min each, and subsequently, they were stained with DAPI (1 μg/mL) for 30 min, washed with PBS twice, and mounted on a clean slide. For each larval bleeding sample, three random images were captured using a fluorescent microscope (Nikon E800) with a 20× objective lens for RFP, GFP, and DAPI channels. The number of hemocytes positive with and without bacteria was quantified by ImageJ manually, and phagocytic efficiency was calculated.

Live imaging of circulating macrophages

Third instar (L3) wandering larvae (*Hml*$^{\Delta}$*-Gal4 UAS-2xEGFP*/+ and *Hml*$^{\Delta}$*-Gal4 UAS-2xEGFP/+; Drice*$^{2c8/\Delta1}$) were bled in 20μL S2 media containing 2.5% insulin (Sigma-Aldrich Cat# I0516), 10% FBS (Sigma-Aldrich Cat#RM9955) and 1μL bacterial (RPF expressing *E. coli*) suspension from overnight culture on a clean bridge slide using coverslips as spacers. Slides were covered with coverslips, so the media containing hemocytes was sandwiched between the coverslip and slide space. Time-lapse imaging was carried out using a confocal microscope, taking pictures of the green and red channels every 30 s.

Embryo live imaging

Drosophila embryos at the desired developmental stage were collected from overnight eggs laying in the embryo collection chamber. Subsequently, these embryos underwent a dechorionation process involving a 5-min treatment with 4% bleach, followed by two rinses with 1X PBS. The dechorionated embryos were carefully positioned on a slide with having a drop of halocarbon oil (Sigma-Aldrich Cat# H8898). The embryos were immersed in oil, and a cover glass was placed over them. Time-lapse imaging was performed using a confocal microscope, capturing images at 30-s intervals for both the green and red channels.[85] Different zoom settings were applied during the imaging process to obtain various magnification levels or fields of view as needed.

Microscopy and image processing

All samples were imaged in a Zeiss LSM-900 confocal microscope using Zen software (version 3.4) under a 20× objective with a zoom of 1.0 and a 40× objective with a zoom of 0.5 and used a 2.0 μm optical section interval in all images otherwise specified in the figure legend. For imaging of samples on different days, an optimal confocal setting was used. On the other hand, daily conditions for experimental and control samples are the same. All images were processed using ImageJ software (NIH, USA) (available at ImageJ.nih.gov/ij), and Adobe Photoshop 2021 (version 22.4.2) was used to make the figure panel. Adobe Illustrator cc 2018 (version 22.1) and pictures from bioicons.com and BioRender were used for the schematic model and graphical abstract preparation. Images of lymph glands are a maximum intensity projection of the stack of the middle third of the samples; it allows for visibility of the inside of the LG, which can be covered by the cortical zone region in a maximum intensity projection of the entire LG, specified in the figure legend. The lymph glands boundary is demarcated by a white dotted line for clarity.

Quantification of lymph gland phenotypes

All quantification was done using ImageJ software (NIH, USA). The number of γH2Av, Hnt, and Dcp-1 positive cells and colocalization of γH2Av with Dcp-1, γH2Av with *GC3Ai*, Hnt with *L-CasExpress L-Trace* was counted manually for both lobes of the primary lymph gland, and analyzed separately. To determine the mean fluorescent intensity of MMP1 staining, the single ROI (an 80 × 80 μm square ROI) of the lymph gland lobes of the maximum intensity projection image was utilized.[109] For volume measurement of multichannel images, first, all channels of images were separated, and one specific threshold was chosen that fit best for the actual staining and kept constant throughout the measurement. The thresholding procedure is used in image processing to select pixels of interest based on the intensity of the pixel values. After that, the "Measure stack" plugin[61] was used to find the fluorescent area (DAPI, GFP, Draper, and P1) of each optical section. Then, the fluorescent area in each optical section was added and multiplied with stack interval (2μm) to determine the volume. For better representation, the primary lobe of the lymph gland has been represented and outlined in white dashed lines.

RNA isolation, quantitative reverse transcription PCR analysis

Total RNA was isolated from one hundred primary lobes of wandering 3rd instar larval lymph glands using Trizol reagent following the manufacturer's recommended protocol (Sigma-Aldrich, Cat# T9424). The RNA pellets were resuspended in 15μL of DEPC-MQ water, and after the pellets were dissolved, their quantitative estimation was done using spectrophotometric analysis. Subsequently, 1μg of each RNA sample was incubated with 1U of RNase-free DNase I (Thermo Fisher Scientific, Cat# 89836) for 30 min at 37°C to eliminate residual DNA. Following the standard cDNA preparation protocol, the first-strand cDNA was synthesized from these

incubated samples. The prepared cDNA was subjected to a real-time PCR machine using forward and reverse primer pairs of the target genes. Real-time PCR was done by using 5 μL of qPCR master mix (SYBR Green, Genetix, Cat# PKG025-A), 2 picomol/μL of each primer per reaction in 10μL of the final volume in ABI 7500 real-time PCR machine (Applied Biosystems). The relative fold change in mRNA expression for different genes was calculated using the comparative C_T method to assess changes in gene expression. Data normalization was done using *Rp49* as an internal control. For each gene, three independent biological replicates were used. The following primers are used for this study:

Drep1 (Forward) 5′-AAACAAAGCCATGGAGACTGCAG-3′
Drep1 (Reverse) 5′-AGACAGCCTTCTTAATGTTGCGTG-3′
Drep4 (Forward) 5′- CCTGCTCATCGGTTGCGAC-3′
Drep4 (Reverse) 5′- GTTTCCTCGTCGCCCAAGTG-3′
Rp49 (Forward) 5′- TTGAGAACGCAGGCGACCGT-3′
Rp49 (Reverse) 5′- CGTCTCCTCCAAGAAGCGCAAG-3′

QUANTIFICATION AND STATISTICAL ANALYSIS

All experiments were repeated at least three times, and one representative image was shown. All images are representative of 3 or more independent biological experiments, and ‘n’ represents the number of lymph gland lobes. In the quantification graphs, control groups are different for their respective experimental sets because experiments are performed on different days. All the statistical tests for the respective experiments were carried out using Microsoft Excel 2019 and GraphPad Prism 9. All the *p*-values represent unpaired two-tailed Student’s t-tests to determine statistical significance. The significance level is indicated by an * for $p \leq 0.05$, ** for $p \leq 0.01$, *** for $p \leq 0.001$, **** for $p \leq 0.0001$, and by ns for not significant, $p > 0.05$.

Cell Reports, Volume *43*

Supplemental information

Transient caspase-mediated activation of caspase-activated DNase causes DNA damage required for phagocytic macrophage differentiation

Deepak Maurya, Gayatri Rai, Debleena Mandal, and Bama Charan Mondal

Figure S1

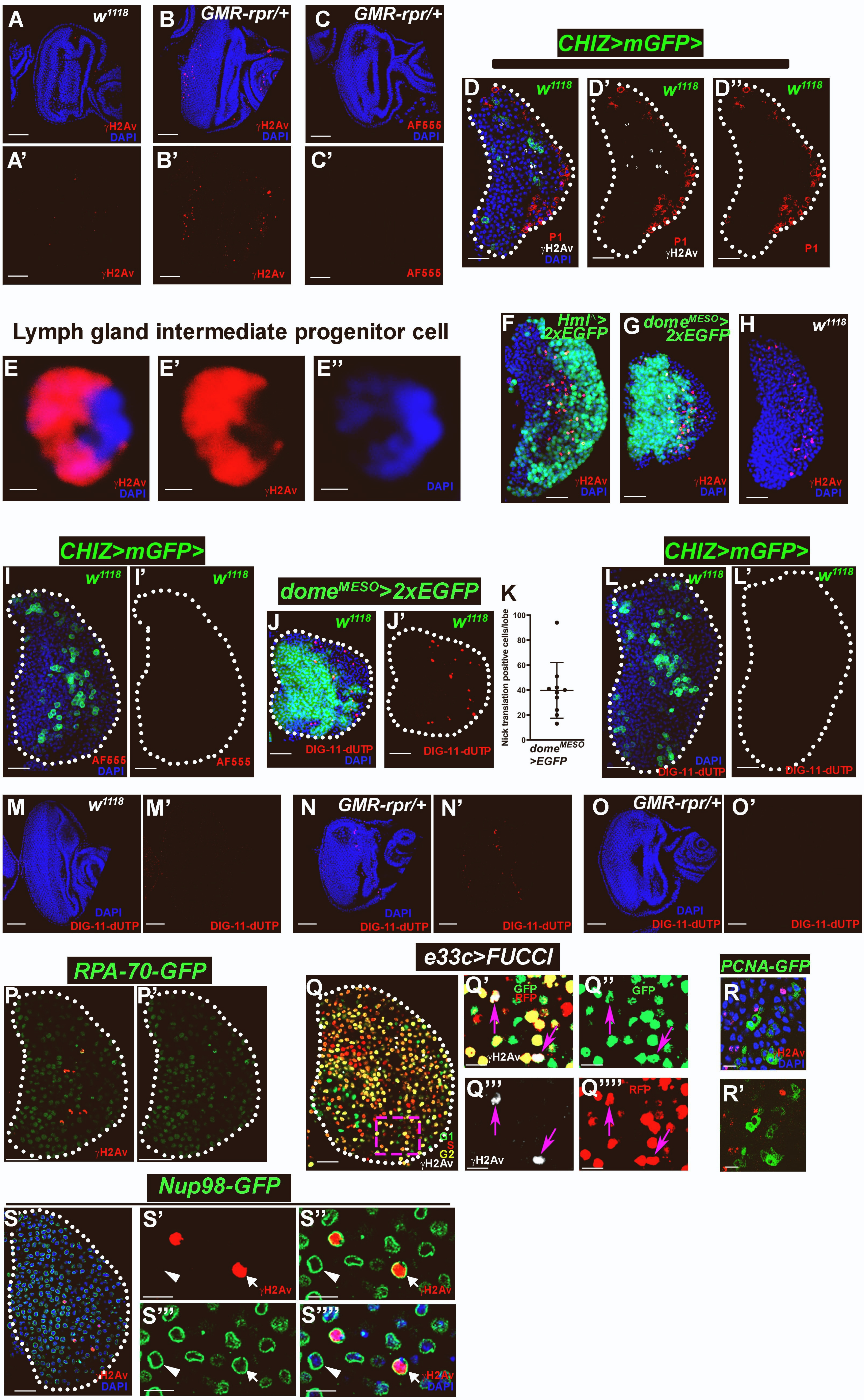

A
w1118
γH2Av
DAPI
B
GMR-rpr/+
C
GMR-rpr/+
AF555
CHIZ>mGFP>
D
D'
D''
P1
Lymph gland intermediate progenitor cell
E
E'
E''
F
HmlΔ> 2xEGFP
G
domeMESO> 2xEGFP
H
I
I'
domeMESO>2xEGFP
J
J'
DIG-11-dUTP
K
Nick translation positive cells/lobe
100
80
60
40
20
0
domeMESO >EGFP
L
L'
M
M'
N
N'
O
O'
RPA-70-GFP
P
P'
e33c>FUCCI
Q
Q'
Q''
Q'''
Q''''
GFP
RFP
G1
S
G2
PCNA-GFP
R
R'
Nup98-GFP
S
S'
S''
S'''
S''''

Figure S1. DNA damage occurs during the differentiation of lymph gland

(A-C') The γH2Av staining (red) increased in the eye disc expressing rpr (*GMR-rpr/+*) (B-B') in comparison to the control eye disc (w^{1118}) (A-A'). Also, negative control (GMR-rpr/+), which is not incubated with primary antibody (mouse-anti-γH2Av, Cat# UNC93-5.2.1) and only incubated with secondary antibody (AF555, Cat# A31570), shows no staining (C-C').

(D-D'') The co-immunostaining of γH2Av (grey) and mature macrophage marker P1 (red) show no colocalization.

(E-E'') The γH2Av staining (red) covers the entire nuclear region, except the DAPI (blue)-bright heterochromatin region in the lymph gland intermediate progenitor.

(F-H) *Hml*^{Δ}*-Gal4, UAS-2xEGFP/+* (green) (F), *dome*^{MESO}*-Gal4, UAS-2xEGFP*/+ (green) (G) and w^{1118} (H) genetic backgrounds show a similar number of γH2Av positive cells (red) in the differentiating zone.

(I-I') Negative control immunostaining (red) for lymph glands that are incubated without primary antibody (mouse-anti-γH2Av) shows no staining.

(J-J') Nick translation (red) shows the incorporation of DIG-11dUTP in the cells of the differentiating zone (low GFP) of the control lymph gland, indicating DNA strand breaks *dome*^{MESO}*-Gal4, UAS-2xEGFP/+* (green) (n=10).

(K) Quantification of nick translation positive cell number represented in (J-J').

(L-L') The negative control for nick translation (red) for the lymph gland not incubated with the enzyme (DNA polymerase I) shows no incorporation of DIG-11dUTP in the cells.

(M-O') The nick translation (red) increased in the eye disc expressing *rpr* (*GMR-rpr/+*) (N-N') in comparison to the control eye disc (w^{1118}) (M-M'). Also, the negative control (GMR-rpr/+), which is incubated without enzyme (DNA polymerase I), shows no incorporation of DIG-11dUTP in the cells (O-O').

(P-P') γH2Av staining (red) colocalized with *RPA70-GFP* (green) in the lymph gland.

(Q-Q'''') Expression of *e33c-Gal4, UAS-FUCCI* shows the cell cycle status of lymph gland G2 (yellow), G1 (green), and S (red) phases (Q) and the colocalization of G2 cells with γH2Av (grey). The insets show the colocalization of γH2Av-positive cells with GFP and RFP, indicated by the arrows in the G2 phase (Q'-Q"").

(R-R') γH2Av staining (red) in *PCNA-GFP* (green) cells as S phase markers do not colocalize in the lymph gland, nuclei stained with DAPI (blue).

(S-S'''') γH2Av staining (red) in *Nup98-GFP* (green) with DAPI (blue) (S) and high magnification image of γH2Av in *Nup-98-GFP* background only γH2Av (S'), γH2Av with *Nup98-GFP* shows intact nuclear membrane (S''), only *Nup98-GFP* (S''') and γH2Av, *Nup98-GFP* with DAPI (S''''). Arrow marks the γH2Av positive nuclei and intact nuclear pore complexes compared with γH2Av negative (marked arrowhead) nuclei in the lymph gland.

All images are from the wandering third instar lymph gland except images A-C' and M-O' from the wandering third instar eye disc. Scale bars: 25µm in all images except 50 µm in (A-C' and M-O'), 10µm in (Q'-R' and S'-S''''), 1µm in (E-E''). All images are single optical sections except images (F-J' and L-L'), which are the maximum intensity projections of the middle third optical sections. Nuclei stained with DAPI (blue). The lymph glands boundary is demarcated by a white dotted line for clarity. Error bars, mean ± SD. All images are representative of 3 or more independent biological experiments, and 'n' represents a number of lymph gland lobes.

Figure S2

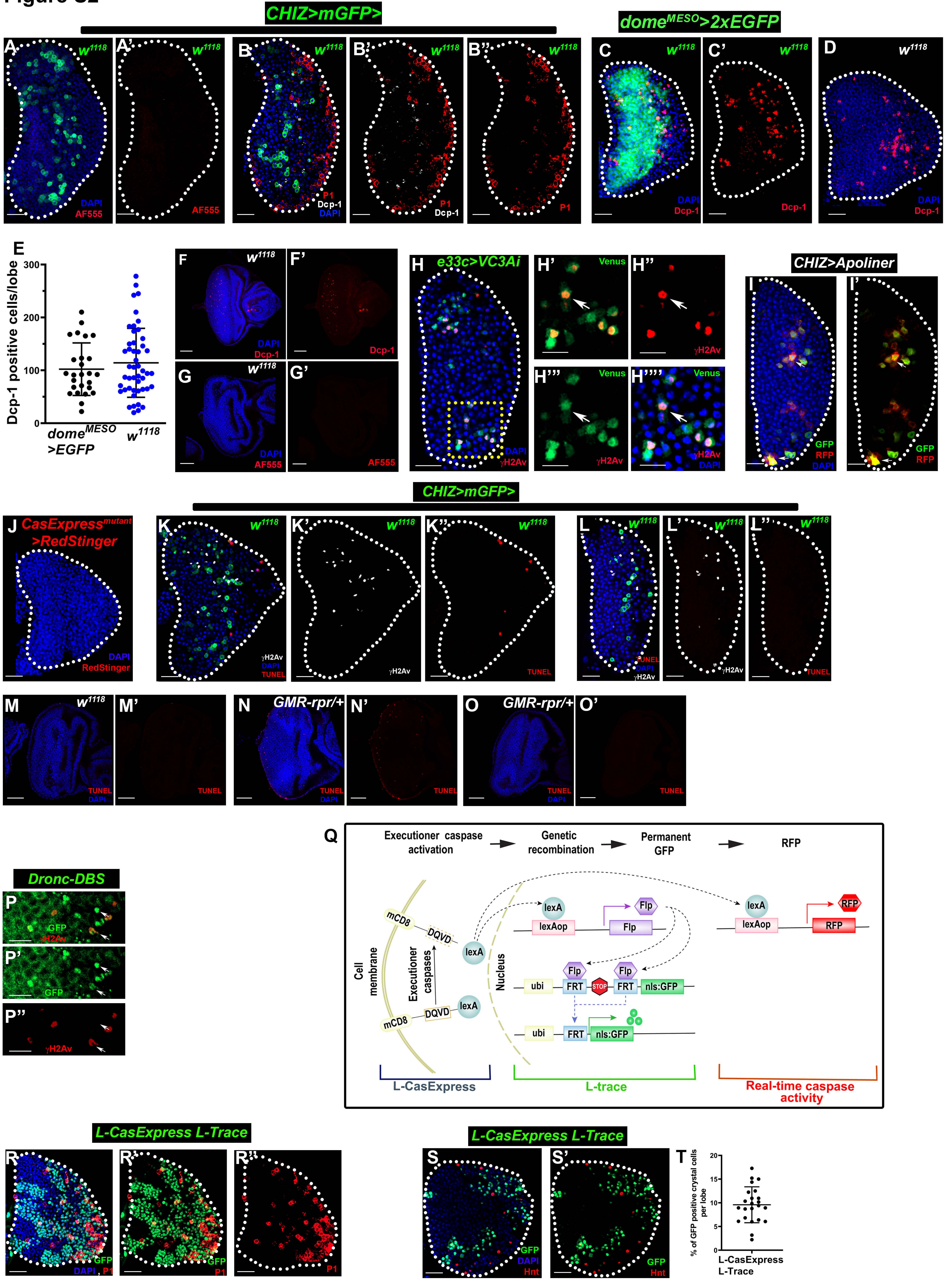
CHIZ>mGFP>
dome^MESO>2xEGFP
Dcp-1 positive cells/lobe
dome^MESO >EGFP
w1118
e33c>VC3Ai
CHIZ>Apoliner
CasExpress^mutant >RedStinger
GMR-rpr/+
Dronc-DBS
Executioner caspase activation
Genetic recombination
Permanent GFP
RFP
Cell membrane
Executioner caspases
Nucleus
L-CasExpress
L-trace
Real-time caspase activity
L-CasExpress L-Trace
% of GFP positive crystal cells per lobe

Figure S2. DNA breaks and active caspase in differentiating progenitor blood cells

(A-A') Negative-control immunostaining (red) shows no staining for lymph glands incubated without primary antibody (anti-Dcp1, CST Cat# 9578S) and only with secondary antibody (AF555, Cat# A31572).

(B-B'') The co-immunostaining of Dcp-1 (grey) and mature macrophage marker P1 (red) show no colocalization.

(C-D) Dcp-1 staining (red) is shown in *dome^{MESO}-Gal4, UAS-2xEGFP/+* progenitors mark green (C-C') (n=26) and *w^{1118}* (D) (n=48) genetic background of the lymph gland in the intermediate zone.

(E) Quantification of Dcp-1 positive cell number in (C-D).

(F-G') The Dcp-1 staining (red) found in wild-type control eye disc (*w^{1118}*) (F-F'); however, negative control, which is incubated without primary antibody (cleaved-Dcp-1) and only incubated with secondary antibody (AF555), shows no staining (G-G').

(H-H'''') Fluorescent reporter (green) of executioner caspase (*e33c-Gal4, UAS-VC3Ai*) are shown with γH2Av staining (red) (H). Insets show venus (green) colocalization with γH2Av (arrow).

(I-I') Expression of *CHIZ-Gal4, UAS-Apoliner* show membrane RFP, membrane GFP, and nuclear GFP (marks caspase-active cell); arrow indicates caspase-active cell.

(J) Expression of *CasExpress^{Mutant}-Gal4, UAS- RedStinger* does not show executioner activity in the lymph gland, which is used as a control for *CasExpress-Gal4* (Figure 2J).

(K-K'') The lymph glands γH2Av-positive cells (grey) are not high-intensity TUNEL-positive cells (red).

(L-L'') Negative control for TUNEL staining (red) for lymph gland which is not incubated with enzyme (terminal deoxynucleotidyl transferase) shows no TUNEL positive cells but γH2Av-positive cells present (grey).

(M-O') The TUNEL staining (red) increased in the eye disc expressing rpr (*GMR-rpr/+*) (N-N') in comparison to the control eye disc (*w^{1118}*) (M-M'). Also, negative control (GMR-rpr/+), which is not incubated with enzyme (terminal deoxynucleotidyl transferase), shows no TUNEL-positive cells (O-O').

(P-P'') High-magnification image of γH2Av (red) staining in *Drice-Based-Sensor-GFP* (*DBS-GFP*; green) (P), only γH2Av (P'') and only GFP (P') shows γH2Av colocalization with low DBS-GFP cells.

(Q) A model showing the mechanistic principle underlying how the *L-CasExpress L-Trace* works (adapted from Sun G *et al.* 2021).

(R-R'') Mature macrophage marker P1 staining (red) in the *L-CasExpress L-Trace* (*lex-Aop-Flp::Ubi-FRT-STOP-FRT-GFP/+; L-caspase/+*) show colocalization with lineage trace GFP suggesting cascade active cells survive and become mature macrophage and only P1 (R'').

(S-S') Crystal cell marker Hnt staining (red) in the *L-CasExpress L-Trace* (*lex-Aop-Flp::Ubi-FRT-STOP-FRT-GFP/+; L-caspase/+*) show significantly less colocalization with lineage trace GFP (S) and only Hnt (S').

(T) Quantification of the percentage of crystal cells in *L-CasExpress L-Trace* (*lex-Aop-Flp::Ubi-FRT-STOP-FRT-GFP/+; L-caspase/+*) colocalized with lineage trace GFP(green) and DAPI (blue) (n=23).

All images are from the wandering third instar lymph gland except images F-G' and M-O' from the wandering third instar eye disc. Scale bars: 25µm in all images except 50 µm in (F-

G’ and M-O’) and 10µm in (H’-H’’’’ and P-P’’). All images are maximum intensity projections of the middle third optical sections except images (B-B’’, F-I’, K-P’’ and S-S’), which are single optical sections. Nuclei stained with DAPI (blue). The lymph glands boundary is demarcated by a white dotted line for clarity. Error bars, mean ± SD. All images are representative of 3 or more independent biological experiments, and ‘n’ represents the -number of lymph gland lobes.

Figure S3

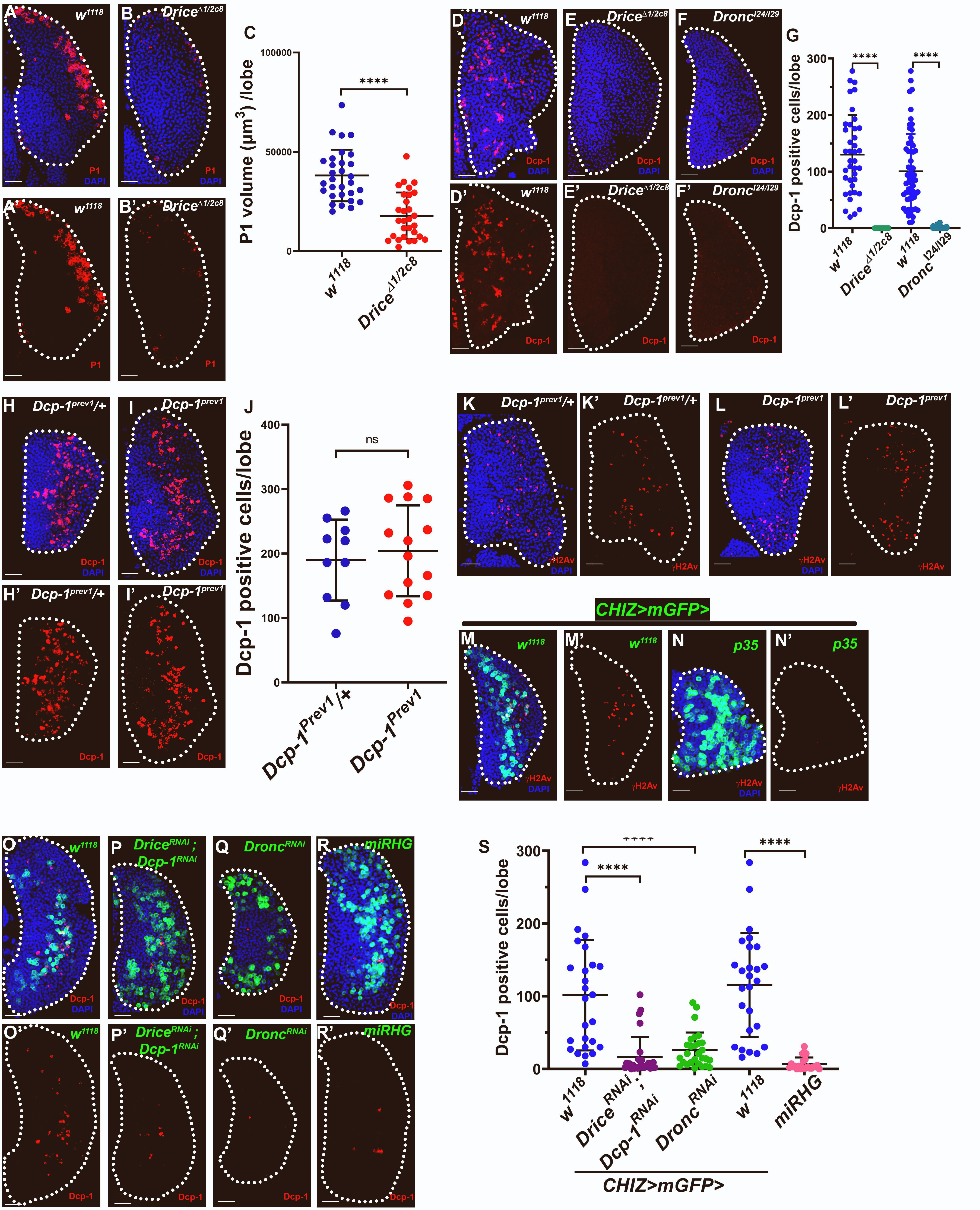

A w1118
B DriceΔ1/2c8
P1
DAPI
A' w1118
B' DriceΔ1/2c8
C
P1 volume (μm3) /lobe
100000
50000
0
****
w1118
DriceΔ1/2c8
D w1118
E DriceΔ1/2c8
F DroncI24/I29
Dcp-1
DAPI
D' w1118
E' DriceΔ1/2c8
F' DroncI24/I29
G
Dcp-1 positive cells/lobe
300
200
100
0
****
****
w1118
DriceΔ1/2c8
w1118
DroncI24/I29
H Dcp-1prev1/+
I Dcp-1prev1
H' Dcp-1prev1/+
I' Dcp-1prev1
J
Dcp-1 positive cells/lobe
400
300
200
100
0
ns
Dcp-1Prev1 /+
Dcp-1Prev1
K Dcp-1prev1/+
K' Dcp-1prev1/+
L Dcp-1prev1
L' Dcp-1prev1
γH2Av
CHIZ>mGFP>
M w1118
M' w1118
N p35
N' p35
O w1118
P DriceRNAi ; Dcp-1RNAi
Q DroncRNAi
R miRHG
O' w1118
P' DriceRNAi ; Dcp-1RNAi
Q' DroncRNAi
R' miRHG
S
Dcp-1 positive cells/lobe
300
200
100
0
****
****
****
w1118
DriceRNAi ; Dcp-1RNAi
DroncRNAi
w1118
miRHG
CHIZ>mGFP>

Figure S3. Caspase-mediated DNA breaks needs for macrophage differentiation

(A-B') Mature macrophage marker P1 staining (red) in control *w*^{1118} (A-A') (n=30), which is significantly reduced in *Drice*^{Δ1}/*Drice*^{2c8} (B-B') (n=29).

(C) Quantification of P1 volume in (A-B').

(D-F') Dcp-1-positive cells (red) in comparison to control (*w*^{1118}) sets (D-D') (n=39 and 59), absent in *Drice*^{Δ1}/*Drice*^{2c8} (E-E') (n=41) and *Dronc*^{I29}/*Dronc*^{I24} (F-F') (n=58) mutants.

(G) Quantification of Dcp-1-positive cell number in (D-F').

(H-I') Dcp-1-positive cells (red) in Dcp-1 heterozygous mutant (*Dcp-1*^{Prev1}/+) (H-H') (n=10) and homozygous mutant (*Dcp-1*^{Prev1}) (I-I') (n=14) remain unchanged.

(J) Quantification of Dcp-1-positive cell number in (H-I')

(K-L') γH2Av -positive cells (red) in Dcp-1 heterozygous mutant (*Dcp-1*^{Prev1}/+) (K-K') and homozygous mutant (*Dcp-1*^{Prev1}) (L-L') remain unchanged.

(M-N') The *CHIZ> mGFP*-driven *UAS-p35* background (N-N') shows very few γH2Av-positive cells (red) compared to the control (M-M').

(O-R') γH2Av-positive cells(red) in comparison to control sets [*CHIZ>mGFP/+* (O-O') (n=26 and n=26), *CHIZ>mGFP* driven *RNAi, UAS-Drice*^{RNAi}*; UAS-Dcp-1*^{RNAi} (P-P') (n=28), *UAS-Dronc*^{RNAi} (Q-Q') (n=28), *UAS-miRHG* (R-R') (n=23) show significantly less γH2Av-positive cells.

(S) Quantification of γH2Av-positive cell number in (O-R').

All images show a 25µm scale bar, with maximum intensity projections of the middle third optical section of the wandering third instar larval lymph gland lobe. Nuclei stained with DAPI (blue). The lymph glands boundary is demarcated by a white dotted line for clarity. ****P < 0.0001 ns- not significant. Error bars, mean ± SD. Control groups are different for their respective experimental sets because experiments are performed on different days. All images are representative of 3 or more independent biological experiments, and 'n' represents a number of lymph gland lobes.

Figure S4

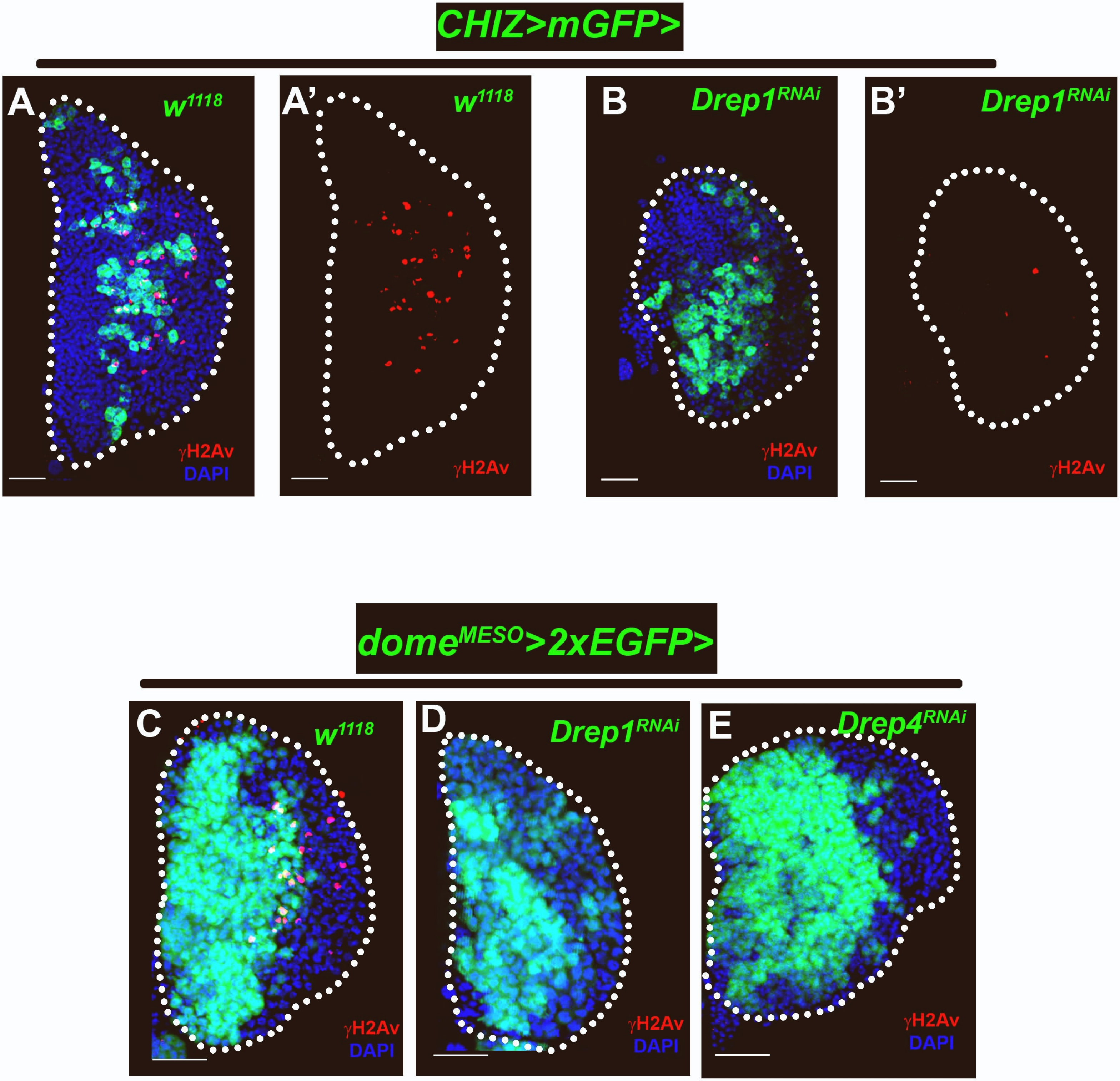
CHIZ>mGFP>
A
w1118
γH2Av
DAPI
A'
w1118
γH2Av
B
Drep1RNAi
γH2Av
DAPI
B'
Drep1RNAi
γH2Av
domeMESO>2xEGFP>
C
w1118
γH2Av
DAPI
D
Drep1RNAi
γH2Av
DAPI
E
Drep4RNAi
γH2Av
DAPI

Figure S4. Caspase Activated DNase induces DNA breaks required for macrophage differentiation

(A-B') Depletion of *Drosophila* ICAD [(*CHIZ>mGFP; UAS-Drep1*^{RNAi} (BL65944 as 2nd RNAi line) (B-B') in the intermediate progenitors causes less γH2Av positive cells (red), GFP cell marks intermediate progenitors (green) compared to control *CHIZ>mGFP/*+ (A-A').

(C-E) Depletion of *Drosophila* ICAD (*dome*^{MESO}*-Gal4, UAS-2xEGFP; UAS-Drep1*^{RNAi} *VDRC8357*) (D) and CAD (*dome*^{MESO}*-Gal4, UAS-2xEGFP*; *UAS-Drep4*^{RNAi}) (E) in the progenitors leads to significantly less γH2Av positive cells (red) in the lymph glands compared to control, *dome*^{MESO}*-Gal4, UAS-2xEGFP*/+ (C). GFP cell marks progenitors (green).

All images are from wandering third instar lymph gland lobes and show a 25µm scale bar and maximum intensity projections of the middle third optical section. Nuclei stained with DAPI (blue). The lymph glands boundary is demarcated by a white dotted line for clarity.

Figure S5

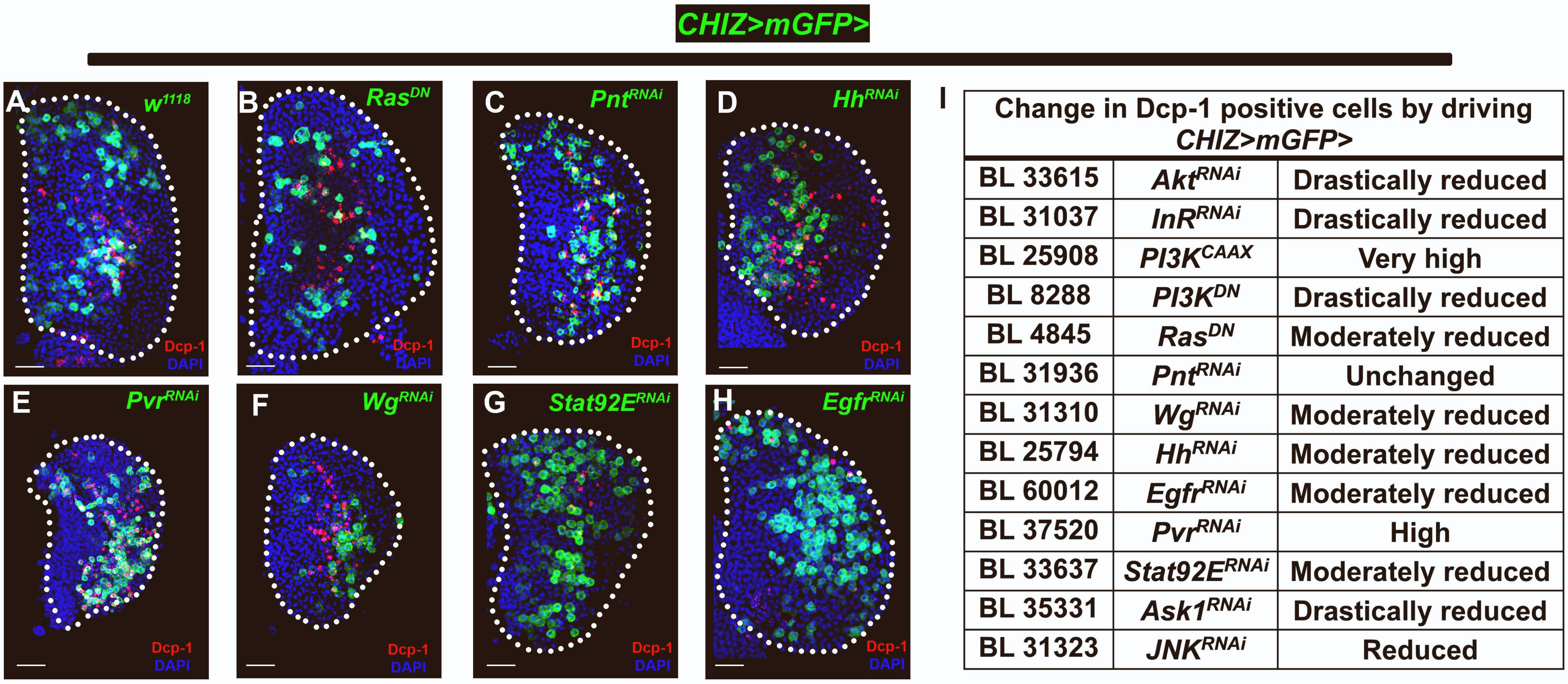

I

Change in Dcp-1 positive cells by driving CHIZ>mGFP>		
BL 33615	*Akt^{RNAi}*	Drastically reduced
BL 31037	*InR^{RNAi}*	Drastically reduced
BL 25908	*PI3K^{CAAX}*	Very high
BL 8288	*PI3K^{DN}*	Drastically reduced
BL 4845	*Ras^{DN}*	Moderately reduced
BL 31936	*Pnt^{RNAi}*	Unchanged
BL 31310	*Wg^{RNAi}*	Moderately reduced
BL 25794	*Hh^{RNAi}*	Moderately reduced
BL 60012	*Egfr^{RNAi}*	Moderately reduced
BL 37520	*Pvr^{RNAi}*	High
BL 33637	*Stat92E^{RNAi}*	Moderately reduced
BL 35331	*Ask1^{RNAi}*	Drastically reduced
BL 31323	*JNK^{RNAi}*	Reduced

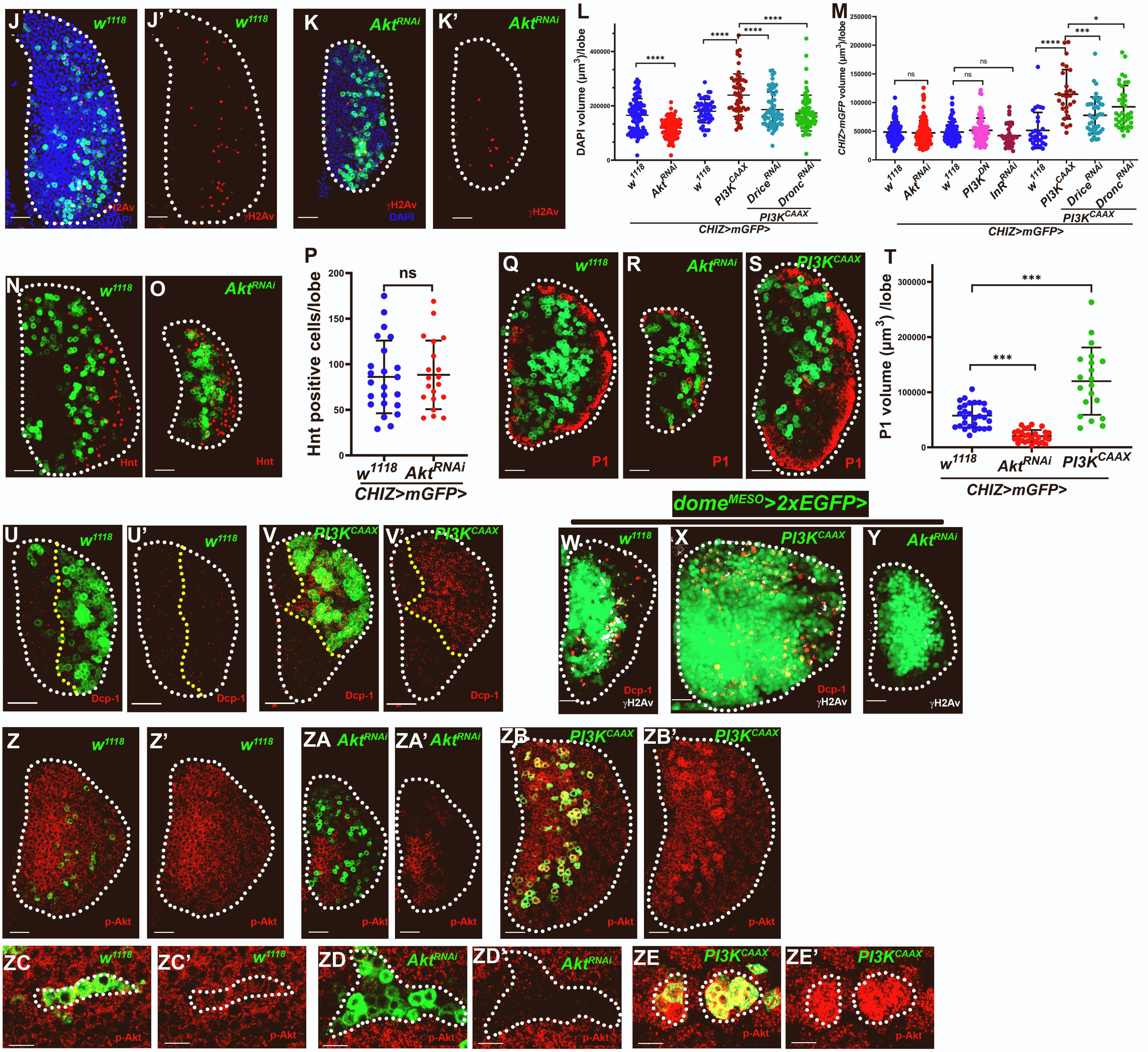

Figure S5. InR/PI3K/Akt signaling regulates caspase activity and DNA breaks in macrophage differentiation

(A-I) Screening of several signaling pathways to check the involvement of caspase regulation in the lymph gland: knockdown of genes in the differentiating progenitors using *CHIZ>mGFP* (green) driver, *UAS-Ras^{DN}* (B), *UAS-Pnt^{RNAi}* (C), *UAS-Hh^{RNAi}* (D)*, UAS-Pvr^{RNAi}* (E)*, UAS-Wg^{RNAi}* (F)*, UAS-stat92E^{RNAi}* (G) *and UAS-Egfr^{RNAi}* (H) show change in the Dcp-1 positive cells mention in (I) as compared to control (*CHIZ>mGF*P/+) (A).

(J-K') *CHIZ>mGFP* (green) driven *Akt^{RNAi}* (BL 31701 as 2nd RNAi line) (K-K') has fewer H2Av-positive cells (red) than the control (J-J').

(L) Quantification of lymph gland size by measuring the DAPI volume in control sets, *CHIZ>mGFP/+* (96) and significantly reduced in *Akt^{RNAi}* (n=102), control *CHIZ>mGFP/+* (n=50) significantly high in *PI3K^{CAAX}* (n=58), and rescued in *PI3K^{CAAX}; Drice^{RNAi}* (n=63) and *PI3K^{CAAX}*; *Dronc^{RNAi}* (n=69).

(M) Quantification of the GFP positive cells volume in several control sets, *CHIZ>mGFP/+* (122) and *CHIZ>mGFP* driven *Akt^{RNAi}* (n=119), control *CHIZ>mGFP/+* (n=83) and *PI3K^{DN}* (n=78), *InR^{RNAi}* (n=34) show no change in GFP+ cells volume. However, *CHIZ>mGFP* driven *PI3K^{CAAX}* (n=28) show high GFP+ cells' volume and partially rescued in *PI3K^{CAAX}; Drice^{RNAi}* (n=41) and *PI3K^{CAAX}*; *Dronc^{RNAi}* (n=38) compared to control *CHIZ>mGFP/+* (n=30) group.

(N-O) *CHIZ>mGFP* driven *Akt^{RNAi}* (n=20) (O) do not show the difference in crystal cells (Hnt staining in red) number compared to in control, *CHIZ>mGFP/+* (n=23) (N).

(P) Quantification of the Hnt positive cell number in (N-O).

(Q-S) *CHIZ>mGFP*-driven *Akt^{RNAi}* (n=28) (R) shows less P1 (red), a macrophage marker, and *CHIZ>mGFP* driven *PI3K^{CAAX}* (n=20) (S) shows high P1 as compared to control, *CHIZ>mGFP/+* (n=22) (Q).

(T) Quantification of the P1 volume in (Q-S).

(U-V') Dcp-1 staining (red) in control, *CHIZ>mGFP/+* (U-U'), and CHIZ>mGFP driven PI3KCAAX (V-V') shows high Dcp-1 staining in early third-instar larvae (48 hours after hatching at 29°C) in CHIZ+ cells.

(W-Y) γH2Av (grey) and Dcp-1 (red) co-staining in control *dome^{MESO}-Gal4, UAS-2xEGFP/+* (W) and *dome^{MESO}-Gal4, UAS-2xEGFP/+* driven *PI3K^{CAAX}* (W) where high number of γH2Av and Dcp-1 positive cells found only in low GFP cells, whereas *dome^{MESO}-Gal4, UAS-2xEGFP/+* driven *Akt^{RNAi}* (Y) show less γH2Av positive cells.

(Z-ZB') Lymph gland exhibits active Akt signaling shown by p-Akt staining (red) in control, *CHIZ>mGFP/+* (Z-Z') (green), but *CHIZ>mGFP* driven *Akt^{RNAi}* (ZA-ZA'), show less p-Akt staining, and *PI3K^{CAAX}* (ZB-ZB') show high p-Akt staining.

(ZC-ZE') High magnification images of p-AKT staining (red) in control, *CHIZ>mGFP/+* (ZC-ZC'), and *CHIZ>mGFP* driven *Akt^{RNAi}* (ZD-ZD') shows less staining in CHIZ^{+} cells, and *PI3K^{CAAX}* (ZE-ZE') shows high staining in CHIZ^{+} cells.

All images are from wandering third instar lymph gland lobes except image U-V', the early third instar lymph gland lobe. Scale bars: 25µm in all images except 10µm in (ZC-ZE') with maximum intensity projections of the middle third optical sections except image Z-ZE', which are single optical sections of the lymph glands. Control groups are different for their respective experimental sets because experiments are performed on different days. Nuclei stained with DAPI (blue). The lymph glands boundary is demarcated by a white dotted line for clarity. *P < 0.05 ***P < 0.001, ****P < 0.0001, ns- not significant. Error bars, mean ± SD. All images are representative of 3 or more independent biological experiments, and 'n' represents the number of lymph gland lobes.

Figure S6

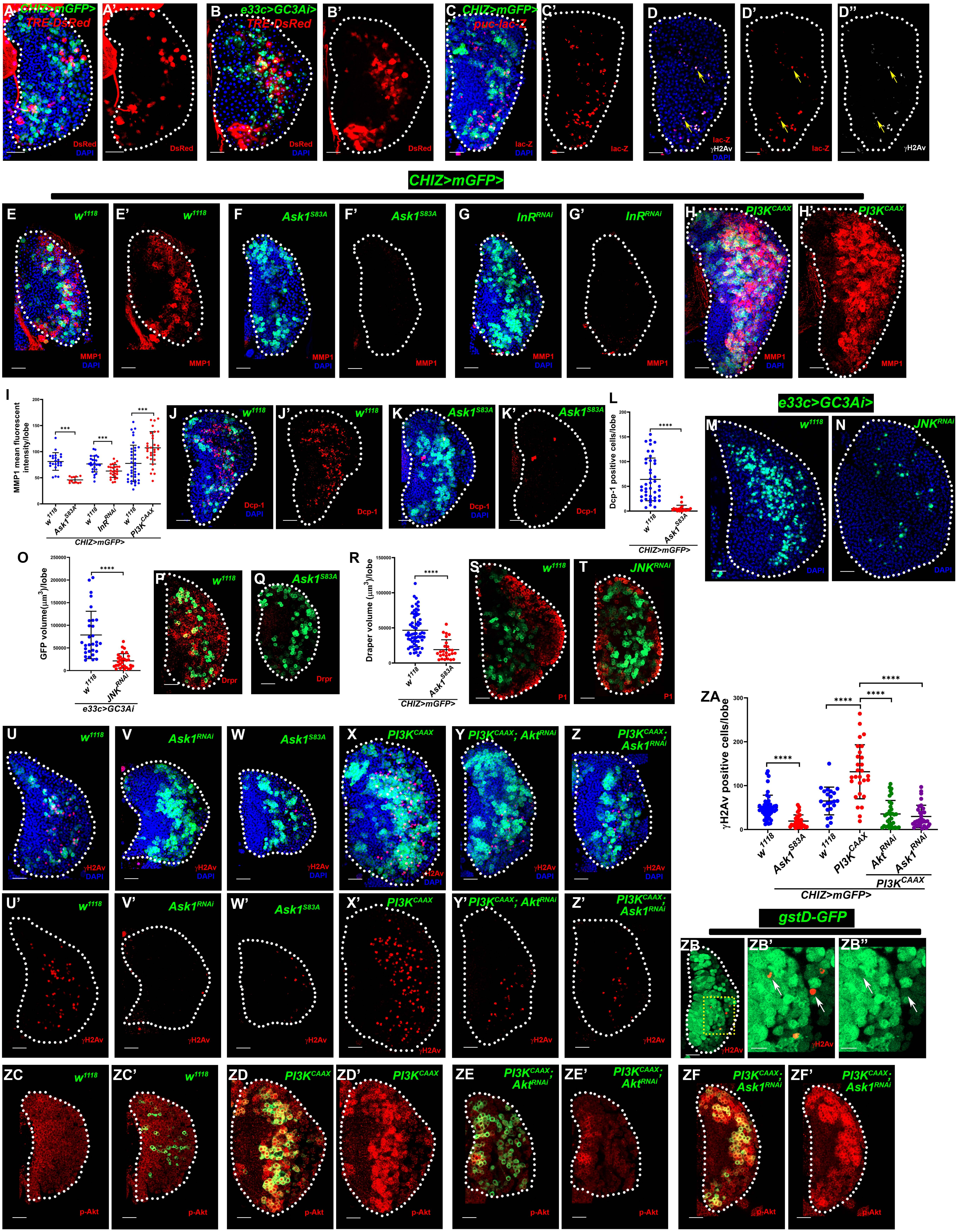

CHIZ>mGFP>
e33c>GC3Ai>
gstD-GFP
MMP1 mean fluorescent intensity/lobe
Dcp-1 positive cells/lobe
GFP volume(μm³)/lobe
Draper volume (μm³)/lobe
γH2Av positive cells/lobe

Figure S6. InR/PI3K/Akt signaling via Ask1/JNK axis regulate caspase activity

(A-A') JNK signaling activity reporter *TRE-DsRed*-positive (red) cells found in the differentiating progenitors (green) (*CHIZ>mGFP/+*; *TRE-DsRed/+*).

(B-B') *TRE-DsRed*-positive cells colocalized with the caspase active cells (GC3Ai) (green) in the intermediate zone of the lymph gland (*UAS-GC3Ai/+; e33c-Gal4/ TRE-DsRed*).

(C-D'') The JNK signaling activity reporter puc-lacZ (pucE69) shows JNK signaling activity (red) in the differentiating progenitors (green) (CHIZ>mGFP/+; pucE69/+), which is colocalized with γH2Av-positive cells(D-D').

(E-H') JNK signaling activity using MMP1 staining (red) in *CHIZ>mGFP* (green) driven *Ask1*^{S83A} (n=12) (F-F') and *InR*^{RNAi} (n=27) (G-G') were severely reduced and drastically high in *PI3K*^{CAAX} (n=30) (H-H') as compared to their respective control sets, *CHIZ>mGFP/+* (n=23, n=26, and n=38) (E-E').

(I) Quantification of mean fluorescent intensity of MMP1 in (E-H').

(J-K') *CHIZ>mGFP* driven *Ask1*^{S83A} (n=38) (K-K') shows Dcp-1-positive cells (red) significantly decrease compared to the control, *CHIZ>mGFP/+* (n=42) (J-J').

(L) Quantification of Dcp-1-positive cells in (J-K').

(M-N) Loss of JNK in the lymph gland (*UAS-GC3Ai/+; e33c-Gal4/UAS-JNK*^{RNAi}) (n=30) (N) shows severely reduced caspase-active (GC3Ai) cells compared to control, (*UAS-GC3Ai/+; e33c-Gal4/+*) (n=28) (M).

(O) Quantification of Caspase-positive cells GC3Ai volume in (M-N).

(P-Q) Draper staining (red) in *CHIZ>mGFP* driven *Ask1*^{S83A} (n=41) (Q) significantly decreased compared to control, *CHIZ>mGFP/+* (n=64) (P).

(R) Quantification of Draper volume in (P-Q).

(S-T) P1 staining (red) in *CHIZ>mGFP* driven *JNK*^{RNAi} (T) significantly decrease compared to control, *CHIZ>mGFP/+* (S).

(U-Z') *CHIZ>mGFP* driven *Ask1*^{RNAi} (BL32646 as 2nd RNAi line) (V-V') and *JNK1*^{RNAi} (n=21) (W-W') show reduced γH2Av-positive cells (red) as compared to respective control, *CHIZ>mGFP/+* (n=47) (U-U'). Depletion of Akt [*CHIZ>mGFP; UAS-PI3K*^{CAAX}*; UAS- Akt*^{RNAi} (n=35)] (Y-Y') and Ask1 [*CHIZ>mGFP; UAS-PI3K*^{CAAX}*; UAS- Ask1*^{RNAi} (n=28) (Z-Z')] in intermediate progenitors [control *CHIZ>mGFP/+* (n=38) (U-U')] rescue the high γH2Av-positive cells (red) phenotype of *PI3K*^{CAAX} overexpression [*CHIZ>mGFP; UAS-PI3K*^{CAAX} (n=35)] (X-X').

(ZB-ZB'') γH2Av (red) positive cells in the lymph gland display low *gstD-GFP/+* (green) (ZB), and high magnification images (ZB'-ZB'') show γH2Av positive cells in low gstD-GFP (ROS marker) cells indicated by an arrow.

(ZC-ZF') Depletion of Akt (*CHIZ>mGFP; UAS-PI3K*^{CAAX}*; UAS- Akt*^{RNAi}) (ZE-ZE') in intermediate progenitors (control *CHIZ>mGFP/+*) (ZC-ZC') reduces the high p-Akt staining (red) phenotype of *PI3K*^{CAAX} overexpression (*CHIZ>mGFP; UAS-PI3K*^{CAAX}) (ZD-ZD') however the depletion of Ask1 (*CHIZ>mGFP; UAS-PI3K*^{CAAX}*; UAS-Ask1*^{RNAi}) (ZF-ZF') fail to reduce the high p-Akt staining (red) phenotype of *PI3K*^{CAAX} overexpression and remain same as *PI3K*^{CAAX} overexpression [*CHIZ>mGFP; UAS-PI3K*^{CAAX}] (ZD-ZD').

All images from the wandering third instar lymph gland lobe show a 25µm scale bar except image (ZB'-ZB''), where the scale bar is 10µm and maximum-intensity projections of the middle third optical sections except images (B-B', D-D'' and ZB-ZF'), which are single optical

sections of the lymph glands. Nuclei stained with DAPI (blue). Control groups are different for their respective experimental sets because experiments are performed on different days. Nuclei stained with DAPI (blue). The lymph glands boundary is demarcated by a white dotted line for clarity. ***P < 0.001, ****P < 0.0001, ns- not significant. Error bars, mean ± SD. All images are representative of 3 or more independent biological experiments, and ‘n’ represents the number of lymph gland lobes.

Figure S7

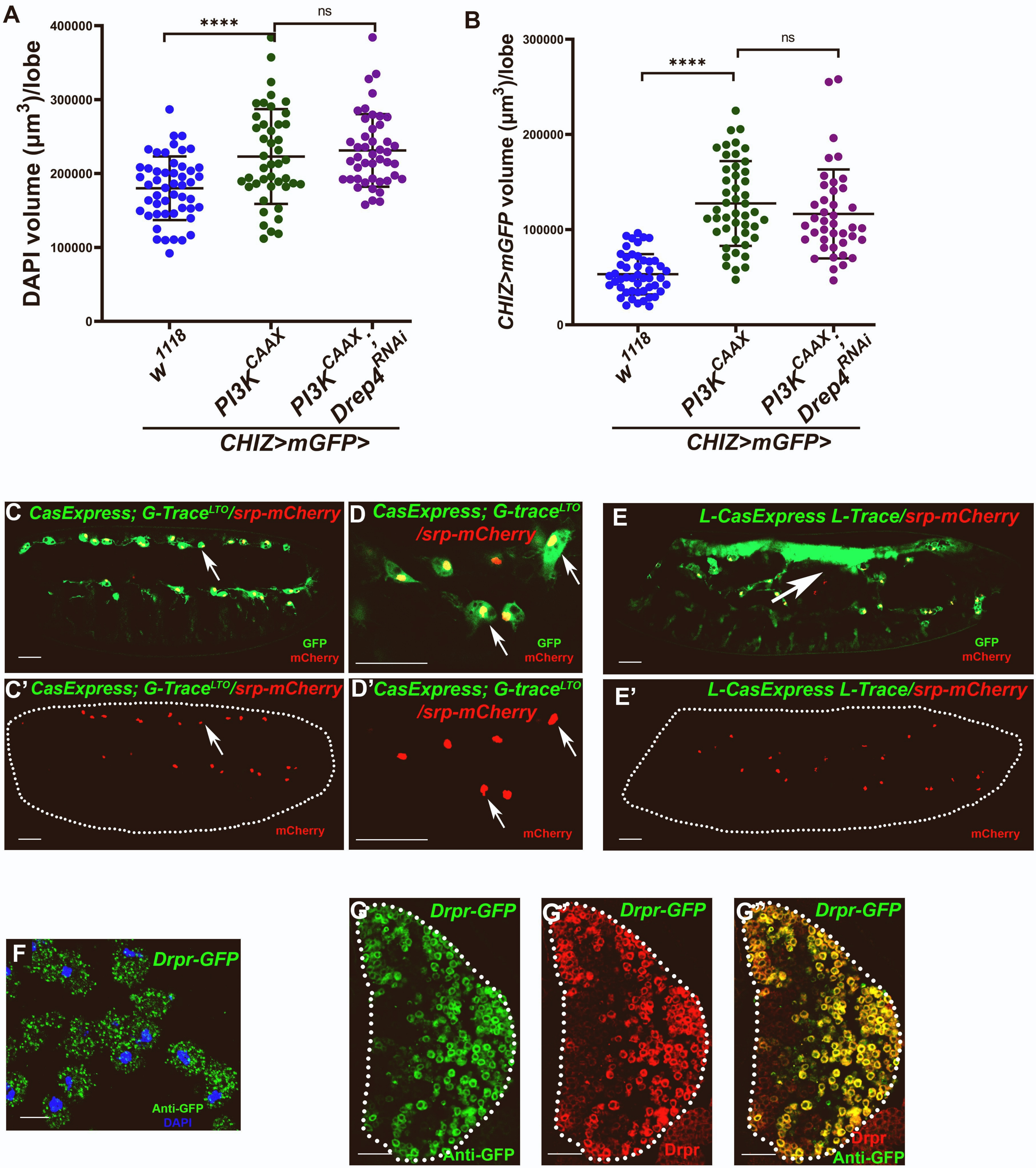

A
DAPI volume (μm³)/lobe
400000
300000
200000
100000
0
****
ns
w1118
PI3KCAAX
PI3KCAAX ;
Drep4RNAi
CHIZ>mGFP>
B
CHIZ>mGFP volume (μm³)/lobe
300000
200000
100000
0
****
ns
w1118
PI3KCAAX
PI3KCAAX ;
Drep4RNAi
CHIZ>mGFP>
C CasExpress; G-TraceLTO/srp-mCherry
GFP
mCherry
D CasExpress; G-traceLTO /srp-mCherry
GFP
mCherry
E L-CasExpress L-Trace/srp-mCherry
GFP
mCherry
C' CasExpress; G-TraceLTO/srp-mCherry
mCherry
D' CasExpress; G-traceLTO /srp-mCherry
mCherry
E' L-CasExpress L-Trace/srp-mCherry
mCherry
F Drpr-GFP
Anti-GFP
DAPI
G Drpr-GFP
Anti-GFP
G' Drpr-GFP
Drpr
G'' Drpr-GFP
Drpr
Anti-GFP

Figure S7. Developmental PI3K/Akt signaling regulates caspase/CAD activation for phagocytic macrophage differentiation.

(A) Quantification of DAPI volume in control, *CHIZ>mGFP/+* (n=50), and *CHIZ>mGFP* driven *UAS-PI3K*^{CAAX} (n=44) have significantly high GFP volumes, which remain high in rescue *UAS-PI3K*^{CAAX}*; UAS-Drep4*^{RNAi} (n=45).

(B) Quantification of GFP volume in control, *CHIZ>mGFP/+* (n=49) and *CHIZ>mGFP* driven *UAS-PI3K*^{CAAX} (n=47) have significantly high GFP volume, which remains high in rescue *UAS-PI3K*^{CAAX}*; UAS-Drep4*^{RNAi} (n=41).

(C-D') Embryonic macrophages (stage 13) marked by *srp-mCherry* show experience of caspase lineage activity (*CasExpress; G-Trace*^{LTO}) (C-C') also shows in high magnification (D-D'), marked by arrows.

(E-E') Embryonic macrophages (stage 13) marked by *srp-mCherry* experience caspase lineage *L-CasExpress L-Trace* (*lex-Aop-Flp::Ubi-FRT-STOP-FRT-GFP/+; L-caspase/+*) GFP (green) dorsal closure (marked by arrow) and macrophages.

(F) Circulating blood cells of third instar *Draper-GFP* larvae show Draper (green) punctate staining.

(G-G'') *Draper-GFP* expression using anti-GFP (green) (G) and staining of anti-Draper (red) antibody (G') and merged image of both (G'') of 3rd instar larval lymph gland.

All images show a 25µm scale bar except image F, where the scale bar is 10µm. All images are single optical sections. Nuclei stained with DAPI (blue). For clarity, the lymph gland and embryo boundary are demarcated by a white dotted line. All images are representative of 3 or more independent biological experiments, and 'n' represents the number of lymph gland lobes. ****P < 0.0001, ns- not significant. Error bars, mean ± SD

Also see Movies S1, S2, and S3.
